# Multi-laboratory validation of the xMAP—Food Allergen Detection Assay: A multiplex, antibody-based assay for the simultaneous detection of food allergens

**DOI:** 10.1371/journal.pone.0234899

**Published:** 2020-07-09

**Authors:** Eric A. E. Garber, Chung Y. Cho, Prasad Rallabhandi, William L. Nowatzke, Kerry G. Oliver, Kodumudi Venkat Venkateswaran, Neeraja Venkateswaran

**Affiliations:** 1 Office of Regulatory Science, Center for Food Safety and Applied Nutrition, Food and Drug Administration, College Park, Maryland, United States of America; 2 Radix BioSolutions, Georgetown, Texas, United States of America; 3 Omni Array Biotechnology LLC, Rockville, Maryland, United States of America; 4 Tetracore, Inc., Rockville, Maryland, United States of America; University of Houston, UNITED STATES

## Abstract

The increasing prevalence of individuals with multiple food allergies and the need to distinguish between foods containing homologous, cross-reactive proteins have made the use of single-analyte antibody-based methods (e.g., ELISAs) sometimes insufficient. These issues have resulted in the need to conduct multiple analyses and sometimes employ orthogonal methods like mass spectrometry or DNA-based methods for confirmatory purposes. The xMAP Food Allergen Detection Assay (xMAP FADA) was developed to solve this problem while also providing increased throughput and a modular design suitable for adapting to changes in analytical needs. The use of built-in redundancy provides the xMAP FADA with built-in confirmatory analytical capability by including complementary antibody bead sets and secondary analytical end points (e.g., ratio analysis and multi-antibody profiling). A measure of a method’s utility is its performance when employed by analysts of varying expertise in multiple laboratory environments. To gauge this aspect, a multi-laboratory validation (MLV) was conducted with 11 participants of different levels of proficiency. The MLV entailed the analysis of incurred food samples in four problematic food matrices, meat sausage, orange juice, baked muffins, and dark chocolate. Except for a couple of instances, involving two confirmatory components in the analysis of baked muffins, the allergenic foods were detected by all participants at concentrations in the analytical samples comparable to ≤ 10 μg/g in the original food sample. In addition, despite high levels of inter-lab variance in the absolute intensities of the responses, the intra-laboratory reproducibility was sufficient to support analyses based on the calibration standards and direct comparison controls (DCCs) analyzed alongside the samples. In contrast, ratio analyses displayed inter-laboratory %CV (RSD_R_) values < 20%; presumably because the ratios are based on inherent properties of the antigenic elements. The excellent performance of the xMAP FADA when performed by analysts of varying proficiency indicates a reliability sufficient to meet analytical needs.

## Introduction

Over 15 million Americans have at least one food allergy [1]. The only way these individuals can avoid an allergic reaction entails not consuming products that contain the allergenic food. In 2004 the Food Allergen Labeling and Consumer Protection Act (FALCPA) was passed to facilitate the consumer’s ability to determine which products to avoid while maintaining a diverse, healthy diet [2]. As part of the enforcement of FALCPA, the FDA must be able to analyze a diverse group of foods for the presence of any of the allergenic foods specified in the act. Complicating the analytical process is the increasing prevalence of people with multiple food allergies [3, 4], as well as the need to detect unknown amounts of allergenic food ranging from trace levels (e.g., micrograms per oral portion) to substantial levels (e.g., >10%). In addition, the complexity of the world market and need to distinguish between related foods containing homologous cross-reactive proteins makes single-analyte methods, such as the commonly available commercial ELISA test kits, insufficient for many circumstances. Alternative, orthogonal methods, such as PCR and mass spectrometry are still under development and not yet universally recognized as suitable for routine regulatory enforcement and as such are not routinely used by contract and governmental laboratories.

In 2014, the FDA with Radix BioSolutions developed a novel xMAP-based multiplex assay for the simultaneous detection of 14 food allergens plus gluten [5], and sesame [6] based on principles associated with ELISA technology. The xMAP Food Allergen Detection Assay (xMAP FADA) entailed two extraction protocols, buffered-detergent (using either Phosphate Buffered Saline with 0.05% Tween^®^-20 or UD Buffer) and reduced-denatured (0.5% SDS/2% β-mercaptoethanol). The buffered-detergent extracts are interrogated using a cocktail consisting of 29 antibodies conjugated to different color-coded bead sets. Specifically, two antibodies (bead set numbers denoted as -x, -y) for almond (-12, -13), Brazil nut (-14, -15), cashew (-18, -19), coconut (-20, -21), egg (-25, -26), gluten (-27, -28), hazelnut (-29, -30), macadamia (-33, -34), milk (-35, -36), peanut (-37, -38), pine nut (-39, -42), pistachio (-43, -44), soy (-45, -46), and walnut (-47, -48) and one antibody for crustacean seafood (-22). The reduced-denatured extracts are analyzed using a cocktail containing one antibody for egg (-65), peanut (-72), and gluten (-73) and two for milk (-66, -67).

The xMAP FADA uses built-in redundancy by having two or more bead sets for each allergen target in a single simultaneous assay, which is not possible in conventional ELISAs. By ensuring concurrence of results for the two or more bead sets per allergen target in a given sample, the probability of false positives and false negatives can be lowered. This is by virtue of its design and incorporation of the AssayChex^™^ bead sets designed by Radix BioSolutions, Ltd., (Georgetown, TX). The AssayChex^™^ bead sets assess four technical aspects of xMAP performance; namely, instrumental performance, ‘non-specific’ binding, detector antibody, and streptavidin-phycoerythrin binding. As such, the need to obtain control material samples, identical to the sample being analyzed but certified as allergen-free, to rule out false positives and false negatives is eliminated. Further, through the use of ratio analysis between complementary bead sets (e.g., almond-12:almond-13) and multi-antibody profiling it is possible to detect and distinguish between homologous, cross-reactive antigenic foods. Several papers describing the performance of the xMAP FADA as both a research tool and for the analysis of regulatory samples have been published [7–10].

The xMAP FADA has also undergone extensive single lab validation (SLV) examining the performance of the assay to detect each of the targeted food allergens individually or as a mixture in the presence of food extracts and when incurred into buffer, orange juice, dark chocolate, pancake batter, and baked muffins [11], and assay robustness study under various experimental conditions [12]. The xMAP FADA successfully detected the analytes in almost all cases with limits of detection (LoDs) considerably less than the lowest calibration standard (S1), indicative of a potential flexibility to extend the dynamic range should greater sensitivity be desired. As observed with commercial ELISA test kits that employ a buffered-detergent extraction protocol, the recoveries for analyte spiked into food extracts varied typically between 50–150% and decreased when incurred into processed foods, but on only three occasions was it impossible to detect analyte. In contrast, less problems were observed in detecting incurred analytes upon extracting the samples using the reduced-denatured protocol; similar to the performance observed with ELISAs based on a reduced-denatured extraction protocol.

To examine inter-laboratory / inter-analyst variability in the performance of the xMAP FADA, an 11 laboratory multi-laboratory validation (MLV) was performed of analytes incurred in meat-sausage, orange juice, baked muffin, and dark chocolate. To gauge inter-analyst performance, the participants were deliberately chosen to reflect a diverse level of experience. Only two participants were proficient in performing the xMAP FADA. The remaining nine participants were either novices or inexperienced with either the xMAP FADA or xMAP technology; specifically, five participants had prior experience running ELISAs and were provided with two days training on the xMAP FADA and two practice samples, two participants were experts on xMAP technology with no experience with food allergen analysis, one participant had prior experience running ELISAs and limited experience on xMAP technology, and one was new to both xMAP and food allergen analysis. All eleven laboratories generated data that was included in the analyses. The only substantive problems were an inability to ship the meat samples to one of the participants in a foreign country and another laboratory inadvertently failed to specify in the data collection program that the instrument should monitor the results for two analytes/four of the 29 bead sets in the buffered-detergent protocol. Otherwise, the MLV was a success with only minimal analytical problems, all involving the baked muffin samples. As expected, variance across the laboratories was considerable when comparing the absolute median fluorescent intensity (MFI) responses. However, ratio analysis, which is based on inherent physical properties of the antigen, displayed inter-laboratory variations for the analytes at 0.5 μg/mL in the analytical samples derived from meat at 15%, orange juice at 15%, baked muffins at 26%, and dark chocolate at 17%. At lower concentrations the %CV values increased with some analytes not being detected with an MFI in excess of the lowest calibration standard (S1).

## Materials and methods

### Reagents

Phosphate buffered saline (PBS, cat# P5368), Tween^®^-20 (cat# P9416), and wheat gluten (cat# G5004) were purchased from Sigma-Aldrich Inc. (St. Louis, MO). BD Difco skim milk powder (cat# DF0032-17-3) and Sodium Dodecyl Sulfate (SDS, cat# 28312) were purchased from Fisher Scientific (Waltham, MA). Whole, raw nuts and legumes were acquired from nuts.com as previously described [5]. All other reagents were of the highest technical grade available. Ingredients (all allergen-free) to prepare the food samples are described below.

### Food samples

All food samples were prepared from ingredients acquired locally and selected based on being allergen-free. The primary focus in preparing the food samples was assuring consistent, accurate allergenic food content while exposing the allergenic foods to processing and conditions comparable to consumed products. Thus, the muffin batter was baked at the manufacturer’s recommended temperature of 350°C to generate an edible product.

Meat samples were prepared from ground pure beef, skinless, filler-free hot dogs. The hot dogs were ground in a Robot Coupe^®^ Blixer^®^ 3 Series D (Robot Coupe U.S.A., Inc., Ridgeland, MS) and aliquoted into one-gram portions into 50 mL conical tubes. The allergenic food mixtures were mixed into the meat and stored at -80°C prior to shipping overnight on dry ice.

Orange juice was pulp-free, not from concentrate, pure orange juice. One mL aliquots were pipetted into 50 mL conical tubes to which the allergenic food mixtures were added, mixed, and stored at -80°C prior to shipping overnight on dry ice.

Dark chocolate was pure dark chocolate pellets that consistently tested milk and allergen-free. One-gram aliquots were placed into 50 mL conical tubes, placed for 15 min into a 60°C water bath to melt the chocolate, the allergenic food mixture added and vortexed for 2–3 seconds before being allowed to cool and solidify at room temperature (approx. 22°C). The samples were stored at room temperature and shipped overnight.

Baked muffin samples were prepared using a using a commercial gluten-free rice flour-based cake mix, soy & dairy-free buttery spread (as a substitute for butter), and canola oil. The ingredients were mixed at the ratios on the package instructions (omitting the addition of an egg) to generate the batter. The batter was aliquoted into one-gram portions on mini-cupcake paper wrappers, the allergenic food mixtures mixed into the batter, and baked on a metal pan at 350°C for nine minutes. The baked muffins were stored at 4°C in sealed 50 mL conical tubes until shipped overnight on ice.

The allergen food mixtures incurred into the foods were derived from nonfat dried milk (NFDM) that was multi-analyst validated with multiple commercial ELISA test kits to perform comparable to NIST SRM 1549; NIST SRM 2387 peanut butter; wheat gluten (cat # G5004, Sigma-Aldrich Co.) that has been previous used in analytical studies [13, 14]; organic, dry soy beans a product of ChoripDong (distributed by Seoul Shik Poom, Inc., Flushing NY, USA). Raw, organic (no shell) almonds, hazelnut, and walnuts, and the organic, shredded coconut; were acquired from Nuts.com. The tree nuts and soybeans were ground using an IKA A11 Basic Analytical Mill (Wilmington, NC). The milk and egg stock solutions were prepared at 10% m/v (100,000 ppm) in PBST using a Potter-Elvehjem Tissue Grinder to achieve homogeneity. The legume and tree nut stock solutions were prepared at 1% w/v (10,000 ppm). The wheat gluten was added as a solid to the most concentrated allergenic food mixture (sub-stock 5, SS-5), vortexed and allowed to sit for 2 hours at room temperature prior to four-fold dilution of an aliquot to prepare sub-stock 4 (SS-4), 2.5-fold of an aliquot of SS-4 to prepare SS-3, four-fold dilution of an aliquot of SS-3 to make SS-2, and 2.5-fold dilution of an aliquot of SS-2 to make SS-1. The SS-5 allergenic food mixture used in preparing the meat samples was prepared by mixing a ratio of 1 mL 1% soy: 0.1 mL 10% egg: 0.1 mL 10% NFDM: 10 mg wheat gluten: 1.3 mL PBST. The SS-5 used to prepare the orange juice samples was prepared at a ratio of 1 mL 1% almond: 0.1 mL NFDM: 1 mL 1% soy: 0.4 mL PBST. The SS-5 used to prepare the baked muffin samples was prepared at a ratio of 1 mL 1% coconut: 1 mL 1% walnut: 0.1 mL 10% egg: 0.1 mL 10% NFDM: 10 mg wheat gluten: 0.3 mL PBST. The SS-5 used to prepare the dark chocolate samples was prepared at a ratio of 1 mL 1% hazelnut: 0.1 mL 10% NFDM: 1 mL 1% peanut: 0.4 mL PBST. The 100, 25, 10, 2.5, and 1 ppm (either μg/g or μg/mL) incurred food samples were prepared using 25 μL of SS-5, SS-4, SS-3, SS-2, and SS-1, respectively. By using only 25 μL per gram (or mL) of food the goal was to minimize any artificial dilution of the food matrix. The above selected allergens are possibly present more commonly in similar types of commercially available foods, and we varied the critical concentrations of the appropriate allergens for multi-laboratory validation of the xMAP FADA platform.

### Food sample shipping and storage

All food samples were coded and supplied in 50 mL sealed conical tubes. The meat and orange juice samples were stored frozen and shipped overnight on dry ice. The baked muffin samples were stored at 4°C and shipped on ice, while the dark chocolate samples were stored and shipped at room temperature. In addition to the coded, incurred food samples, all participants were supplied with analyte-free food samples and reference materials (i.e., NIST SRM 2387 peanut reference material, non-fat dried milk reference material multi-analyst validated as comparable to NIST SRM 1549 using ELISA test kits, and wheat gluten reference material used for ELISA studies [13, 14]) for preparing the DCCs.

### XMAP Food Allergen Detection Assay

The xMAP FADA was performed according to the manufacturer’s instructions, which also provides a detailed description of the antibodies [5, S1 Appendix]. Three of the food samples (meat, orange juice, baked muffins) were extracted according to the PBST-based extraction protocol by adding 20 mL of PBST to 1 g sample, vortex, and extracted for 2 hours at room temperature. The dark chocolate samples were extracted using the 40 mL of UD buffer (105 mM sodium phosphate/75 mM NaCl/ 2.5% Difco skim milk powder/0.05% Tween^®^-20) by following the UD buffer extraction protocol. All samples were extracted in triplicate on the day of analysis. The PBST extracts were diluted 10-fold prior to mixing with the bead cocktail, while the UD buffer extracts were diluted 5-fold with UD buffer; net dilution of all food samples being 200-fold prior to mixing with the bead cocktail. The calibration standards, bead cocktail, detection antibody cocktail, streptavidin-phycoerythrin, and direct comparison controls (see S1 Appendix instruction) were prepared on the day of use.

Calibrations standards S0 (buffer, background), S1, S2, S5, and S7 were included in all analyses alongside the samples. The concentrations of the various allergenic food protein extracts in the calibration standards are tabulated in Table 1 along with the equivalent concentrations necessary in the food samples, which undergo 200-fold dilution prior to mixing with the bead cocktail, to generate the same amount as in the calibration standards. Table 2 lists the protein content of the various allergenic foods used to calculate between protein and allergenic food.

**Table 1:** Calibration standards for buffered-detergent analyses & equivalence in food units^a^.

	**ALMOND**			**BRAZIL NUT**			**CASHEW**			**COCONUT**			**CRUST**		
	**as‐is** ^b^	**dil corr** ^c^	**food corr** ^d^	**as‐is**	**dil corr**	**food corr**	**as‐is**	**dil corr**	**food corr**	**as‐is**	**dil corr**	**food corr**	**as‐is**	**dil corr**	**food corr**
	**ng/mL**	**μg/mL**	**ppm**	**ng/mL**	**μg/mL**	**ppm**	**ng/mL**	**μg/mL**	**ppm**	**ng/mL**	**μg/mL**	**ppm**	**ng/mL**	**μg/mL**	**ppm**
**S1**	2.9	0.6	2.7	2.9	0.6	4.4	0.5	0.1	0.6	0.6	0.1	1.7	15	3	17
**S2**	5.2	1	4.9	5.2	1.0	7.8	0.8	0.2	1	1.1	0.2	3.1	28	5.6	31
**S5**	30	6	28	30.0	6.0	45	5	1	5.8	6.7	1.3	19	162	32	181
**S7**	99	20	93	99	20	149	16	3.2	19	22	4.4	62	525	105	588
	**EGG**			**GLUTEN**			**HAZELNUT**			**MACADAMIA**			**MILK**		
	**as‐is**	**dil corr**	**food corr**	**as‐is**	**dil corr**	**food corr**	**as‐is**	**dil corr**	**food corr**	**as‐is**	**dil corr**	**food corr**	**as‐is**	**dil corr**	**food corr**
	**ng/mL**	**μg/mL**	**ppm**	**ng/mL**	**μg/mL**	**ppm**	**ng/mL**	**μg/mL**	**ppm**	**ng/mL**	**μg/mL**	**ppm**	**ng/mL**	**μg/mL**	**ppm**
**S1**	5.8	1.2	2.4	3.7	0.7	0.9	1.2	0.2	1.8	4.8	1	12	1.5	0.3	0.8
**S2**	10	2	4.2	6.6	1.3	1.7	2.1	0.4	3.2	8.7	1.7	22	2.6	0.5	1.5
**S5**	61	12.2	25	39	7.8	9.8	12.0	2.4	18	51	10	129	15	3	8.5
**S7**	198	40	83	125	25	31	40	8	60	165	33	418	49	9.8	28
	**PEANUT**			**PINE NUT**			**PISTACHIO**			**SOY**			**WALNUT**		
	**as‐is**	**dil corr**	**food corr**	**as‐is**	**dil corr**	**food corr**	**as‐is**	**dil corr**	**food corr**	**as‐is**	**dil corr**	**food corr**	**as‐is**	**dil corr**	**food corr**
	**ng/mL**	**μg/mL**	**ppm**	**ng/mL**	**μg/mL**	**ppm**	**ng/mL**	**μg/mL**	**ppm**	**ng/mL**	**μg/mL**	**ppm**	**ng/mL**	**μg/mL**	**ppm**
**S1**	1.5	0.3	1.2	8.1	1.6	11	2.9	0.6	2.9	9.2	1.8	5.2	9.2	1.8	11
**S2**	2.8	0.6	2.2	15.0	3.0	21	5	1	5.2	17	3.4	9.5	17	3.4	20
**S5**	16	3.2	13	85	17	119	30	6	30	96	19	54	96	19	115
**S7**	53	11	42	274	55	383	99	20	98	313	63	175	313	63	376

^*a*^ Concentration of calibration standards after preparation (200-fold dilution of frozen stock and 1.8-fold serial dilutions. S0 is analyte-free, either PBST buffer or UD buffer. Not included in MLV are S6 (1.8-fold dilution of S7), S4 (1.8-fold dilution of S5), and S3 (1.8-fold dilution of S4).

^*b*^ Calibration standards are supplied as ng extractable protein per mL.

^*c*^ Calibration standards are not extracted nor diluted (net 200-fold), thus equivalent concentration a food sample would have to contain.

^*d*^ Equivalent ppm of allergenic food the food sample would have to contain based on the protein content of the allergenic food (see Protein content table).

**Table 2:** Protein content of allergenic foods used as calibrants.

	% Protein	Reference ^a^
Almond	21	raw almonds UPC:014113210638
Brazil Nut	13	raw Brazil nuts UPC 708820008175
Cashew	17	raw cashews UPC 019061198014
Coconut	7	raw coconut UPC 033674100110
Crustacean	18	raw black tiger UPC 857536005029
Egg	48	NIST RM 8445
Gluten	80	Sigma‐Aldrich G5004
Hazelnut	13	raw hazelnuts UPC 03003491412
Macadamia	8	raw macadamia UPC 072989767519
Milk	36	1093, milk dry nonfat (Carnation 36%)
Peanut	25	16095, raw, Virginia; standardized vs NIST SRM 2387
Pine Nut	14	raw pine nuts UPC 041497132430
Pistachio	20	12151, raw pistachio nuts
Soy	36	soybeans UPC 019061190308
Walnut	17	raw walnuts UPC 070038645740

^*a*^ Legumes, tree nuts, and crustacean referenced to entries in USDA, ARS FoodData Central, formerly National Nutrient Database (accessed November 20, 2019; rest as delineated.

### Instrumentation

Inasmuch as the results obtained from xMAP FADA analysis using Luminex^®^ 100/200^™^ (Luminex Corp., Austin, TX), Bio-Plex 200 (Bio-Rad Laboratories, Inc., Hercules, CA), and MagPix^®^ (Luminex Corp., Austin, TX) instrumentation are indistinguishable [11], no restriction was placed on the instruments used in the validation. Thus, nine of the participants employed MagPix^®^ instrumentation and two employed Luminex^®^ 100/200^™^ instrumentation.

### Validation design

The multi-laboratory validation was conducted as a Level IV: Full Collaborative Study according to the Guidelines for the Validation of Chemical Methods in Food, Feed, Cosmetics, and Veterinary Products, 3^nd^ Edition; U.S. Food and Drug Administration Foods Program. October 2019 as delineated in Table 3 [15]. The validation exceeded the requirements in that 11 laboratories participated in this quantitative validation, 4 food matrices of differing physical and chemical properties were used (meat, orange juice, baked muffin, and dark chocolate), allergens were incurred at 5 levels plus one blank, and everything was done in triplicate. To ensure that the MLV reflected inter-laboratory variability in the preparation, extraction, and analysis of the samples, each sample was individually incurred with the appropriate allergens as one gram, the size of an analytical portion, prior to processing. As such, changes in mass associated with processing (e.g., loss of water during muffin baking) did not result in variability of allergen content available for extraction. Specifically, muffin batter samples incurred with 0, 1, 2.5, 10, 25, or 100 μg analyte resulted in 0, 1, 2.5, 10, 25, or 100 μg of potentially extractable analyte in the encoded samples analyzed by the participants. The focus on increased reliability in the amount (μg) of allergenic food present in the test portion is of importance since the dose (μg) ingested, rather than the concentration per unit volume, determines any potential health risk.

**Table 3:** Key validation parameter requirements for chemical methods.

	Level One: Emergency/ Limited Use	Level Two: Single Laboratory Validation	Level Three: Multi-Laboratory Validation	Level Four: Full Collaborative
Number participating labs	1	1	*≥ 2*	8 (quantitative) 10 (qualitative)
Number of matrix sources per matrix*	≥1	≥3 recommended where available	≥3 recommended where available	≥3 recommended where available
Number of analyte(s) spike levels for at least one matrix source**	≥2 spike levels + 1 matrix blank	≥3 spike levels + 1 matrix blank	≥3 spike levels +1 matrix blank	≥3 spike levels +1 matrix blank
Replicates required per matrix source at each level tested/lab	≥2 (quantitative) ≥2 (qualitative)	≥2 (quantitative) ≥3 (qualitative)	≥2 (quantitative) ≥3 (qualitative)	≥2 (quantitative) ≥3 (qualitative)
Replicates required at each level tested per laboratory if only one matrix source used	≥4 (quantitative) ≥6 (qualitative)	≥6 (quantitative) ≥9 (qualitative)	≥3 (quantitative) ≥6 (qualitative)	≥2 (quantitative) ≥6 (qualitative)

*If a variety of food matrices with differing physical and chemical properties are selected, the number of sources for each food sample matrix may be one or more, but if only one food matrix is studied then ≥3 sources are recommended where available. The number of matrix sources may be reduced, particularly if it is difficult to obtain blank matrix sources, as long as the total number of spike levels and matrix combinations are adequate (e.g., 6 replicates or greater at each spike level for quantitative methods and 9 replicates or greater for qualitative methods).

** Number of spike levels is recommended for at least one source of matrix. Other similar sources of matrix (*e*.*g*., within the same category; see Appendix 4) may be studied at one or two spike levels (*e*.*g*., at an action/guidance or tolerance level or close to the lower limit of quantitation/detection).

The eleven laboratories that participated in the validation were chosen to represent a variety of expertise. Thus, no individual laboratory was excluded from inclusion in the data analyses despite an analysis of potential outliers using probabilistic models might support dropping the data from up to three lower performing laboratories (the least proficient). Instead, only on rare occasions were specific data points not included in the processed data based on Extreme Value Analysis. Thus, data was only omitted on the rarest of occasions, in which it displayed characteristics of catastrophic failure that would have been easily spotted by an analyst and by definition could not be used (e.g., generation of negative MFI when adjusted for background or the lack of a dynamic response).

To assist the analysts and re-enforce uniformity, the participants were supplied with the same written instructions (see S1 Appendix). This included a copy of the product insert, instructions on how to configure the instrumentation (the SLV demonstrated that both the Luminex/BioPlex and MagPix instruments generated comparable results [11]), and instructions regarding which coded samples were to be extracted using the PBST Buffered-detergent extraction protocol (meat, orange juice, baked muffins) and which using the UD Buffered-detergent extraction protocol (dark chocolate). The analysts were also instructed to perform a (normally optional) 10-fold dilution with PBST of the PBST-based extracts and a 5-fold dilution with UD Buffer of the UD Buffer-based extracts; the difference reflecting that the PBST extractions entailed a 20-fold dilution of the samples, while the UD Buffer extractions entailed a 40-fold dilution. Included in the analyses, the participants were asked to prepare direct comparison control samples (DCC) which entailed spiking one-gram portions of analyte-free foods with either 50 μg of milk, 10 μg of peanut, or 20 μg of wheat gluten; all done according to detailed procedures provided to the participants. In order to accommodate triplicate direct comparison control samples (DCCs), only the S0, S1, S2, S5, and S7 calibration standards were employed. Lastly, the analysts were instructed to use a microtiter plate as a template to facilitate pipetting and place the diluted extracts into alternating columns to reduce the possibility of inadvertent cross contact. Ten of the eleven laboratories were shipped (overnight) at the same time the food samples and xMAP FADA reagents; the eleventh joined the validation approximately two months later. The FADA reagents were from the same production run (Lot 5) and provided in sufficient quantity for 240 analyses (wells of microtiter plates). Problems were only encountered for international shipping of the meat samples.

## Results and discussion

### Calibration standards

Calibration standards serve a minimum of two essential roles. The one obvious purpose is for determining analyte concentration from standard curve interpolation. Table 1 lists the ng extractable soluble protein per mL for the calibration standards (S3, S4, and S6 were not included in the MLV) and the equivalents of allergenic food (ppm) based on conversion factors taken from the USDA FoodData Central database [16], assuming a 200-fold dilution prior to mixing with the bead set cocktail. Of possibly greater importance is the role calibration standards play as a check on assay performance. Since, the calibration standards are a constant between all production lots and preparation entails just dilution of a stock sample that is stored frozen until use, any changes in performance reflect either changes in the reagents or the ability of the analyst to perform the assay. As such, the performance of the calibration standards in three lots produced over a five-year period provided information on lot-to-lot variability. Further, the calibration standards in the MLV provided an opportunity to examine variability in analyst proficiency without the complexity associated with food sample preparation-extraction and any complications associated with analyzing complex, processed food samples.

### Lot-to-lot variability

The performance of the calibration standards by three lots produced during a five-year period as analyzed by a highly proficient analyst, the same for Lots 3 and 5, is presented in Tables 4 and 5. Tabulated in Table 4 for each lot are the average background MFI for each bead set; MFI generated by the calibration standards after subtracting background (S0); and the MFI generated by DCCs containing 20 ppm gluten (G20), 10 ppm peanut (P10), and 50 ppm non-fat dried milk (50M). The three lots are from the first production run (Lot 1), the third production run used for the SLV, and the fifth production run which was prepared for the MLV; all analyses conducted within six months of production. The Lot 1 and Lot 3 data were derived from triplicate samples while the Lot 5 data represents the average of triplicate samples from each of four separate experiments (n = 3 x 4). Calibration standards S3, S4, and S6 were not included in the Lot 5 analyses. Also included purely for informational purposes in the tables, in orange font, are the averages across the three lots; though such should not be used to analyze data. As expected, all bead sets displayed excellent dynamic responses.

**Table 4:** Lot‐to‐lot variability of calibration standards over five years^a^.

		**ALMOND** ^b^		**BRAZIL NUT**		**CASHEW**		**COCONUT**		**CRUST**	**EGG**		**GLUTEN**		**HAZELNUT**	
		**12**	**13**	**14**	**15**	**18**	**19**	**20**	**21**	**22**	**25**	**26**	**27**	**28**	**29**	**30**
**AVERAGE BACKGROUND MFI**																
**S0**	**Lot 1**	**394**	**193**	**199**	**128**	**243**	**192**	**107**	**133**	**207**			**242**	**245**	**223**	**141**
**S0**	**Lot 3**	**34**	**41**	**58**	**39**	**88**	**50**	**77**	**37**	**56**	**101**	**1111**	**83**	**179**	**88**	**59**
**S0**	**Lot 5**	**65**	**53**	**81**	**51**	**130**	**36**	**122**	**48**	**105**	**398**	**3634**	**102**	**292**	**207**	**48**
**AVERAGE INTENSITIES (MFI) CALIBRATION STANDARDS ABOVE BACKGROUND (minus average S0)**																
**S1**	**Lot 1**	**2409**	**1168**	**403**	**84**	**2948**	**369**	**1978**	**79**	**367**			**405**	**2225**	**719**	**136**
**S1**	**Lot 3**	**2146**	**801**	**1363**	**611**	**2160**	**706**	**1757**	**70**	**492**	**1318**	**2185**	**275**	**775**	**961**	**285**
**S1**	**Lot 5**	**2506**	**990**	**977**	**460**	**2484**	**1200**	**1817**	**68**	**427**	**2294**	**3393**	**243**	**563**	**380**	**329**
**S1**	**av** ^c^	**2354**	**986**	**914**	**385**	**2530**	**758**	**1851**	**72**	**428**	**1806**	**2789**	**308**	**1188**	**686**	**250**
**S2**	**Lot 1**	**3619**	**1639**	**649**	**135**	**4564**	**658**	**3814**	**121**	**632**			**599**	**3264**	**1326**	**253**
**S2**	**Lot 3**	**3595**	**1379**	**2609**	**1112**	**3540**	**1241**	**3374**	**124**	**834**	**2226**	**3916**	**400**	**1163**	**1688**	**473**
**S2**	**Lot 5**	**4526**	**1706**	**1811**	**828**	**4359**	**2047**	**3728**	**119**	**742**	**4215**	**6204**	**290**	**664**	**657**	**570**
**S2**	**av**	**3913**	**1574**	**1690**	**692**	**4154**	**1315**	**3639**	**121**	**736**	**3220**	**5060**	**430**	**1697**	**1224**	**432**
**S3**	**Lot 1**	**5528**	**2862**	**1335**	**267**	**6957**	**1133**	**7663**	**232**	**1261**			**851**	**4284**	**2432**	**452**
**S3**	**Lot 3**	**5533**	**2329**	**4607**	**1957**	**5658**	**2103**	**6151**	**199**	**1434**	**3802**	**6778**	**554**	**1682**	**2845**	**853**
**S3**	**av**	**5531**	**2596**	**2971**	**1112**	**6308**	**1618**	**6907**	**216**	**1348**	**3802**	**6778**	**702**	**2983**	**2638**	**652**
**S4**	**Lot 1**	**7452**	**4431**	**2441**	**481**	**9923**	**1821**	**12234**	**356**	**2228**			**1194**	**6188**	**4355**	**810**
**S4**	**Lot 3**	**7498**	**3525**	**7792**	**3161**	**7806**	**3442**	**9420**	**318**	**2145**	**5791**	**10212**	**752**	**2421**	**4737**	**1411**
**S4**	**av**	**7475**	**3978**	**5117**	**1821**	**8864**	**2632**	**10827**	**337**	**2186**	**5791**	**10212**	**973**	**4305**	**4546**	**1110**
**S5**	**Lot 1**	**9939**	**7042**	**5549**	**1380**	**13761**	**3516**	**17126**	**537**	**4331**			**1832**	**9445**	**6976**	**1497**
**S5**	**Lot 3**	**9420**	**5023**	**11633**	**4935**	**10368**	**5129**	**12917**	**444**	**3343**	**7994**	**13781**	**982**	**3368**	**6822**	**2078**
**S5**	**Lot 5**	**15814**	**7344**	**11895**	**4813**	**12710**	**8768**	**16803**	**466**	**3264**	**13589**	**20555**	**852**	**2425**	**2052**	**2412**
**S5**	**av**	**11724**	**6469**	**9692**	**3709**	**12280**	**5804**	**15615**	**482**	**3646**	**10791**	**17168**	**1222**	**5079**	**5283**	**1996**
**S6**	**Lot 1**	**11980**	**9248**	**10441**	**2635**	**17006**	**5542**	**19627**	**757**	**7603**			**2470**	**13316**	**9643**	**2359**
**S6**	**Lot 3**	**11373**	**6652**	**16208**	**7161**	**13022**	**7311**	**15515**	**616**	**5164**	**10944**	**17899**	**1319**	**4964**	**9654**	**3315**
**S6**	**av**	**11677**	**7950**	**13324**	**4898**	**15014**	**6427**	**17571**	**686**	**6383**	**10944**	**17899**	**1894**	**9140**	**9648**	**2837**
**S7**	**Lot 1**	**14146**	**11823**	**16522**	**4324**	**19358**	**8324**	**21740**	**1021**	**11113**			**2977**	**15646**	**12129**	**3558**
**S7**	**Lot 3**	**12943**	**8191**	**20166**	**9261**	**15302**	**9848**	**17092**	**785**	**7775**	**12234**	**19028**	**1586**	**6151**	**11263**	**4357**
**S7**	**Lot 5**	**21666**	**12109**	**23495**	**10225**	**18867**	**15831**	**21942**	**782**	**7886**	**17578**	**23921**	**1398**	**4778**	**3487**	**4835**
**S7**	**av**	**16251**	**10708**	**20061**	**7937**	**17842**	**11334**	**20258**	**863**	**8925**	**14906**	**21475**	**1987**	**8858**	**8960**	**4250**
**MFI of DIRECT COMPARISON CONTROLS 9DCCs)** ^d^																
**G20**	**Lot 5**	**16**	**5**	**30**	**0**	**5**	**14**	**13**	**1**	**1**	**‐19**	**‐239**	**1893**	**4858**	**4**	**18**
**P10**	**Lot 5**	**8**	**8**	**31**	**6**	**4**	**17**	**13**	**2**	**‐1**	**‐22**	**‐308**	**10**	**28**	**0**	**23**
**M50**	**Lot 5**	**5**	**7**	**34**	**2**	**16**	**18**	**18**	**1**	**‐2**	**‐17**	**‐237**	**9**	**35**	**2**	**23**
		**MACADAMIA**	**MILK**	**PEANUT**	**PINE NUT**	**PISTACHIO**	**SOY**	**WALNUT**								
		**33**	**34**	**35**	**36**	**37**	**38**	**39**	**42**	**43**	**44**	**45**	**46**	**47**	**48**	
**AVERAGE BACKGROUND MFI**																
**S0**	**Lot 1**	**207**	**139**			**203**	**231**	**471**	**128**	**206**	**182**	**171**	**250**	**476**	**182**	
**S0**	**Lot 3**	**116**	**65**	**3176**	**2284**	**42**	**53**	**305**	**42**	**60**	**43**	**47**	**45**	**114**	**51**	
**S0**	**Lot 5**	**131**	**67**	**126**	**293**	**134**	**101**	**511**	**78**	**89**	**59**	**106**	**75**	**411**	**128**	
**AVERAGE INTENSITIES (MFI) CALIBRATION STANDARDS ABOVE BACKGROUND (minus average S0)**																
**S1**	**Lot 1**	**5323**	**234**			**966**	**1197**	**897**	**109**	**1304**	**86**	**3181**	**2293**	**1344**	**887**	
**S1**	**Lot 3**	**4770**	**219**	**1823**	**1118**	**1623**	**1141**	**662**	**72**	**2945**	**228**	**208**	**1662**	**528**	**254**	
**S1**	**Lot 5**	**7968**	**317**	**797**	**655**	**1120**	**749**	**1042**	**116**	**4605**	**313**	**207**	**115**	**1620**	**1080**	
**S1**	**av** ^c^	**6020**	**257**	**1310**	**886**	**1236**	**1029**	**867**	**99**	**2951**	**209**	**1199**	**1357**	**1164**	**740**	
**S2**	**Lot 1**	**8329**	**378**			**1736**	**2224**	**1186**	**158**	**2208**	**167**	**4867**	**3494**	**1958**	**1238**	
**S2**	**Lot 3**	**7435**	**379**	**1887**	**1053**	**3100**	**2249**	**924**	**118**	**4969**	**420**	**368**	**2744**	**922**	**432**	
**S2**	**Lot 5**	**11110**	**558**	**1602**	**1231**	**1908**	**1531**	**1383**	**185**	**8131**	**556**	**347**	**191**	**2939**	**1934**	
**S2**	**av**	**8958**	**438**	**1744**	**1142**	**2248**	**2001**	**1164**	**154**	**5103**	**381**	**1860**	**2143**	**1939**	**1201**	
**S3**	**Lot 1**	**11147**	**727**			**3204**	**4201**	**1520**	**245**	**3441**	**283**	**8594**	**6030**	**3540**	**2469**	
**S3**	**Lot 3**	**9375**	**706**	**2875**	**1669**	**5911**	**4328**	**1220**	**180**	**7885**	**696**	**631**	**4457**	**1667**	**774**	
**S3**	**av**	**10261**	**716**	**2875**	**1669**	**4558**	**4264**	**1370**	**213**	**5663**	**490**	**4613**	**5244**	**2603**	**1622**	
**S4**	**Lot 1**	**13991**	**1284**			**5337**	**7187**	**1858**	**353**	**5067**	**491**	**12159**	**8593**	**5710**	**4536**	
**S4**	**Lot 3**	**11269**	**1195**	**4350**	**2807**	**10396**	**7455**	**1572**	**282**	**11022**	**1182**	**1043**	**6938**	**2841**	**1287**	
**S4**	**av**	**12630**	**1239**	**4350**	**2807**	**7867**	**7321**	**1715**	**318**	**8044**	**837**	**6601**	**7766**	**4276**	**2911**	
**S5**	**Lot 1**	**16309**	**2542**			**8128**	**11365**	**2360**	**663**	**7537**	**1040**	**19470**	**12402**	**7903**	**6583**	
**S5**	**Lot 3**	**12725**	**1821**	**5988**	**3839**	**16014**	**11412**	**1869**	**418**	**13060**	**1875**	**1668**	**10194**	**4702**	**1958**	
**S5**	**Lot 5**	**18774**	**2907**	**8740**	**5726**	**11064**	**11398**	**2509**	**569**	**18861**	**2354**	**1611**	**824**	**13393**	**8914**	
**S5**	**av**	**15936**	**2423**	**7364**	**4782**	**11735**	**11392**	**2246**	**550**	**13153**	**1756**	**7583**	**7807**	**8666**	**5818**	
**S6**	**Lot 1**	**18673**	**3941**			**11163**	**15661**	**2725**	**950**	**10243**	**1633**	**23234**	**15995**	**10028**	**9543**	
**S6**	**Lot 3**	**15277**	**3208**	**8178**	**5629**	**19745**	**14526**	**2174**	**612**	**14816**	**2927**	**2500**	**13766**	**6848**	**2976**	
**S6**	**av**	**16975**	**3575**	**8178**	**5629**	**15454**	**15093**	**2449**	**781**	**12529**	**2280**	**12867**	**14881**	**8438**	**6260**	
**S7**	**Lot 1**	**19713**	**5759**			**12928**	**19007**	**3203**	**1361**	**12210**	**2585**	**23884**	**17320**	**11630**	**11517**	
**S7**	**Lot 3**	**16221**	**4234**	**9176**	**6395**	**21257**	**16561**	**2314**	**781**	**15875**	**4002**	**3396**	**16317**	**8534**	**4017**	
**S7**	**Lot 5**	**22201**	**7068**	**12432**	**8866**	**20573**	**16723**	**2877**	**867**	**21306**	**4935**	**3400**	**1535**	**17805**	**13755**	
**S7**	**av**	**19378**	**5687**	**10804**	**7630**	**18253**	**17430**	**2798**	**1003**	**16464**	**3841**	**10226**	**11724**	**12656**	**9763**	
**MFI of DIRECT COMPARISON CONTROLS 9DCCs)** ^d^																
**G20**	**Lot 5**	**9**	**4**	**20**	**19**	**8**	**10**	**‐48**	**3**	**5**	**2**	**5**	**5**	**32**	**7**	
**P10**	**Lot 5**	**8**	**2**	**108**	**86**	**754**	**369**	**‐47**	**5**	**4**	**4**	**18**	**4**	**24**	**6**	
**M50**	**Lot 5**	**8**	**3**	**13255**	**8645**	**11**	**7**	**‐45**	**2**	**13**	**0**	**4**	**4**	**28**	**4**	

^*a*^ Intensities of the average MFI, after subtracting the average background (S0), of the calibration standards analyzed using three lots of reagents produced over a five year period and used within six months of production. Lot 1 ‐ first production run in 2014 (black font); Lot 3 ‐ production run used for SLV (blue font); Lot 5‐ production run used for MLV (green font). Lot 1 and Lot 3 data are the average of triplicate analyses, Lot 5 is the average of data collected in four experiments, each performed in triplicate. All data were generated by proficient analysts, Lots 3 and 5 by the same analyst.

^*b*^ Target food allergen of antibodies conjugated to the specified bead sets. Lot 1 did not include in its repertoire beads sets 25, 26, 35, and 36.

^*c*^ Averages (orange font) across the Lots are presented for informational purposes since variations between production runs may mathematically result in the appearance of inconsistencies, though each lot displayed proper increases in intensities with concentration.

^*d*^ Direct comparison controls (DCCs) containing 20 ppm gluten (G20), 10 ppm peanut (P10), and 50 ppm NFDM (M50). DCC analyses were only performed using reagents from Lot 5 and the MFI are the averages of DCCs prepared by spiking the specified amount of analyte into analyte‐free food samples, each in triplicate. The meat, orange juice, and baked muffin samples were extracted using the PBST‐based Buffered‐detergent protocol followed by 10‐fold dilution with PBST. The dark chocolate samples were extracted using the UD Buffer Buffered‐detergent protocol followed by 5‐fold dilution with UD buffer. Due to the presence of NFDM in the UD Buffer, the M50 results from the dark chocolate samples were not included in the M50 average. The boxes indicate the bead sets expected to generate a positive response for the specific DCC.

**Table 5:** Lot‐to‐lot variation in signal / noise above background (S/N ‐ 1) of calibration standards over five years^a^.

Calib Std	LOT	ALMOND ^b^		BRAZIL NUT		CASHEW		COCONUT		CRUST	EGG		GLUTEN		HAZELNUT	
		12	13	14	15	18	19	20	21	22	25	26	27	28	29	30
**Signal‐to‐Noise Above Bakground (/N‐1)** ^c^																
**S1**	**Lot 1**	**6**	**6**	**2**	**1**	**12**	**2**	**18**	**1**	**2**			**2**	**9**	**3**	**1**
**S1**	**Lot 3**	**63**	**20**	**24**	**16**	**25**	**14**	**23**	**2**	**9**	**13**	**2**	**3**	**4**	**11**	**5**
**S1**	**Lot 5**	**39**	**19**	**12**	**9**	**19**	**34**	**15**	**1**	**4**	**6**	**1**	**2**	**2**	**2**	**7**
**S1**	**av**	**36**	**15**	**13**	**9**	**19**	**17**	**19**	**1**	**5**	**9**	**1**	**2**	**5**	**5**	**4**
**S2**	**Lot 1**	**9**	**8**	**3**	**1**	**19**	**3**	**36**	**1**	**3**			**2**	**13**	**6**	**2**
**S2**	**Lot 3**	**106**	**34**	**45**	**29**	**40**	**25**	**44**	**3**	**15**	**22**	**4**	**5**	**6**	**19**	**8**
**S2**	**Lot 5**	**70**	**32**	**22**	**16**	**34**	**57**	**30**	**2**	**7**	**11**	**2**	**3**	**2**	**3**	**12**
**S2**	**av**	**62**	**25**	**24**	**15**	**31**	**29**	**37**	**2**	**8**	**16**	**3**	**3**	**7**	**9**	**7**
**S3**	**Lot 1**	**14**	**15**	**7**	**2**	**29**	**6**	**72**	**2**	**6**			**4**	**17**	**11**	**3**
**S3**	**Lot 3**	**163**	**57**	**79**	**51**	**64**	**42**	**80**	**5**	**26**	**38**	**6**	**7**	**9**	**32**	**15**
**S3**	**av**	**88**	**36**	**43**	**26**	**46**	**24**	**76**	**4**	**16**	**38**	**6**	**5**	**13**	**22**	**9**
**S4**	**Lot 1**	**19**	**23**	**12**	**4**	**41**	**9**	**114**	**3**	**11**			**5**	**25**	**20**	**6**
**S4**	**Lot 3**	**221**	**86**	**134**	**82**	**89**	**69**	**122**	**9**	**38**	**57**	**9**	**9**	**14**	**54**	**24**
**S4**	**av**	**120**	**54**	**73**	**43**	**65**	**39**	**118**	**6**	**25**	**57**	**9**	**7**	**19**	**37**	**15**
**S5**	**Lot 1**	**25**	**36**	**28**	**11**	**57**	**18**	**160**	**4**	**21**			**8**	**39**	**31**	**11**
**S5**	**Lot 3**	**277**	**123**	**201**	**128**	**118**	**103**	**168**	**12**	**60**	**79**	**12**	**12**	**19**	**78**	**36**
**S5**	**Lot 5**	**243**	**139**	**147**	**94**	**98**	**246**	**137**	**10**	**31**	**34**	**6**	**8**	**8**	**10**	**50**
**S5**	**av**	**182**	**99**	**125**	**78**	**91**	**122**	**155**	**9**	**37**	**57**	**9**	**9**	**22**	**40**	**32**
**S6**	**Lot 1**	**30**	**48**	**52**	**21**	**70**	**29**	**183**	**6**	**37**			**10**	**54**	**43**	**17**
**S6**	**Lot 3**	**335**	**162**	**279**	**186**	**148**	**146**	**201**	**17**	**92**	**108**	**16**	**16**	**28**	**110**	**57**
**S6**	**av**	**182**	**105**	**166**	**103**	**109**	**88**	**192**	**11**	**65**	**108**	**16**	**13**	**41**	**77**	**37**
**S7**	**Lot 1**	**36**	**61**	**83**	**34**	**80**	**43**	**203**	**8**	**54**			**12**	**64**	**55**	**25**
**S7**	**Lot 3**	**381**	**200**	**348**	**241**	**174**	**197**	**222**	**21**	**139**	**121**	**17**	**19**	**34**	**128**	**74**
**S7**	**Lot 5**	**333**	**228**	**291**	**200**	**146**	**444**	**179**	**16**	**75**	**44**	**7**	**14**	**16**	**17**	**100**
**S7**	**av**	**250**	**163**	**240**	**158**	**133**	**228**	**202**	**15**	**89**	**83**	**12**	**15**	**38**	**66**	**67**
**MFI of DIRECT COMPARISON CONTROLS (DCCs) CONTAIING 20 PPM GLUTEN, 10 PPM PEANUT, AND 50 PPM MILK** ^d^																
**G20**	**Lot 5**	**0**	**0**	**0**	**0**	**0**	**0**	**0**	**0**	**0**	**0**	**0**	**19 17**	**17**	**0**	**0**
**P10**	**Lot 5**	**0**	**0**	**0**	**0**	**0**	**0**	**0**	**0**	**0**	**0**	**0**	**0**	**0**	**0**	**0**
**M50**	**Lot 5**	**0**	**0**	**0**	**0**	**0**	**0**	**0**	**0**	**0**	**0**	**0**	**0**	**0**	**0**	**0**
Calib Std	LOT	MACADAMIA		MILK		PEANUT		PINE NUT		PISTACHIO		SOY		WALNUT		
		33	34	35	36	37	38	39	42	43	44	45	46	47	48	
**Signal‐to‐Noise Above Bakground (/N‐1)** ^c^																
**S1**	**Lot 1**	**26**	**2**			**5**	**5**	**2**	**1**	**6**	**0**	**19**	**9**	**3**	**5**	
**S1**	**Lot 3**	**41**	**3**	**1**	**0**	**39**	**22**	**2**	**2**	**49**	**5**	**4**	**37**	**5**	**5**	
**S1**	**Lot 5**	**61**	**5**	**6**	**2**	**8**	**7**	**2**	**1**	**52**	**5**	**2**	**2**	**4**	**8**	
**S1**	**av**	**43**	**3**	**3**	**1**	**17**	**11**	**2**	**1**	**36**	**4**	**8**	**16**	**4**	**6**	
**S2**	**Lot 1**	**40**	**3**			**9**	**10**	**3**	**1**	**11**	**1**	**28**	**14**	**4**	**7**	
**S2**	**Lot 3**	**64**	**6**	**1**	**0**	**74**	**42**	**3**	**3**	**83**	**10**	**8**	**61**	**8**	**8**	
**S2**	**Lot 5**	**85**	**8**	**13**	**4**	**14**	**15**	**3**	**2**	**91**	**9**	**3**	**3**	**7**	**15**	
**S2**	**av**	**63**	**6**	**7**	**2**	**32**	**22**	**3**	**2**	**62**	**7**	**13**	**26**	**6**	**10**	
**S3**	**Lot 1**	**54**	**5**			**16**	**18**	**3**	**2**	**17**	**2**	**50**	**24**	**7**	**14**	
**S3**	**Lot 3**	**81**	**11**	**1**	**1**	**141**	**82**	**4**	**4**	**131**	**16**	**13**	**99**	**15**	**15**	
**S3**	**av**	**67**	**8**	**1**	**1**	**78**	**50**	**4**	**3**	**74**	**9**	**32**	**62**	**11**	**14**	
**S4**	**Lot 1**	**68**	**9**			**26**	**31**	**4**	**3**	**25**	**3**	**71**	**34**	**12**	**25**	
**S4**	**Lot 3**	**97**	**18**	**1**	**1**	**248**	**141**	**5**	**7**	**184**	**27**	**22**	**154**	**25**	**25**	
**S4**	**av**	**82**	**14**	**1**	**1**	**137**	**86**	**5**	**5**	**104**	**15**	**47**	**94**	**19**	**25**	
**S5**	**Lot 1**	**79**	**18**			**40**	**49**	**5**	**5**	**37**	**6**	**114**	**50**	**17**	**36**	
**S5**	**Lot 3**	**110**	**28**	**2**	**2**	**381**	**215**	**6**	**10**	**218**	**44**	**35**	**227**	**41**	**38**	
**S5**	**Lot 5**	**144**	**44**	**70**	**20**	**83**	**112**	**5**	**7**	**212**	**40**	**15**	**11**	**33**	**70**	
**S5**	**av**	**111**	**30**	**36**	**11**	**168**	**126**	**5**	**7**	**155**	**30**	**55**	**96**	**30**	**48**	
**S6**	**Lot 1**	**90**	**28**			**55**	**68**	**6**	**7**	**50**	**9**	**136**	**64**	**21**	**52**	
**S6**	**Lot 3**	**132**	**49**	**3**	**2**	**470**	**274**	**7**	**15**	**247**	**68**	**53**	**306**	**60**	**58**	
**S6**	**av**	**111**	**39**	**3**	**2**	**263**	**171**	**6**	**11**	**148**	**39**	**95**	**185**	**41**	**55**	
**S7**	**Lot 1**	**95**	**42**			**64**	**82**	**7**	**11**	**59**	**14**	**140**	**69**	**24**	**63**	
**S7**	**Lot 3**	**140**	**65**	**3**	**3**	**506**	**312**	**8**	**19**	**265**	**93**	**72**	**363**	**75**	**79**	
**S7**	**Lot 5**	**170**	**106**	**99**	**30**	**154**	**165**	**6**	**11**	**239**	**83**	**32**	**20**	**43**	**107**	
**S7**	**av**	**135**	**71**	**51**	**17**	**241**	**187**	**7**	**13**	**188**	**63**	**81**	**151**	**48**	**83**	
**MFI of DIRECT COMPARISON CONTROLS (DCCs) CONTAIING 20 PPM GLUTEN, 10 PPM PEANUT, AND 50 PPM MILK** ^d^																
**G20**	**Lot 5**	**0**	**0**	**0**	**0**	**0**	**0**	**0**	**0**	**0**	**0**	**0**	**0**	**0**	**0**	
**P10**	**Lot 5**	**0**	**0**	**0**	**0**	**6**	**4**	**0**	**0**	**0**	**0**	**0**	**0**	**0**	**0**	
**M50**	**Lot 5**	**0**	**0**	**105 29**	**29**	**0**	**0**	**0**	**0**	**0**	**0**	**0**	**0**	**0**	**0**	

^*a*^ The Signal / Noise above background (S/N‐1, background equaling S0) for the calibrations standards analyzed using three lots of reagents produced over a five year period and used within six months of production. Lot 1 ‐ first production run in 2014; Lot 3 ‐ production run used for SLV; Lot 5‐ production run used for MLV. Lot 1 and Lot 3 data are the average of triplicate analyses, Lot 5 is the average of data collected in four experiments, each performed in triplicate. All data were generated by proficient analysts, Lots 3 and 5 by the same analyst.

^*b*^ Target food allergen of antibodies conjugated to the specified bead sets. Lot 1 did not include in its repertoire beads sets 25, 26, 35, and 36.

^*c*^ S/N-1 values were rounded to the nearest integer. S/N‐1 < 1 highlighted in pink; 1 to 2 white; 2‐ 5 yellow; 5 ‐ 10 light green; >10 green. Data presented rounded off to the nearest whole number but color highlighting baed on values to the first decimal. Thus a S/N‐1 of 1.9 will appear as '2' but w/o any highlight while 2.1 would appear as a '2' with yellow highligh.

^*d*^ Direct comparison controls (DCCs) containing 20 ppm gluten (G20), 10 ppm peanut (P10), and 50 ppm NFDM (M50). DCC analyses were only performed using reagents from Lot 5 and the MFI are the averages of DCCs prepared by spiking the specified amount of analyte into analyte‐free food samples, each in triplicate. The meat, orange juice, and baked muffin samples were extracted using the PBST‐based Buffered‐detergent protocol followed by 10‐fold dilution with PBST. The dark chocolate samples were extracted using the UD Buffer Buffered‐detergent protocol followed by 5‐fold dilution with UD buffer. Due to the presence of NFDM in the UD Buffer, the M50 results from the dark chocolate samples were not included in the M50 average. The boxes indicate the bead sets expected to generate a positive response for the specific DCC.

Lot-to-lot variances are not surprising considering the complexity of assembling the reagents. Thus, a more appropriate comparison of the performance by the various lots may be to examine the Signal-to-Noise ratios above background (S/N-1). This data is presented in Table 5 in which the S/N-1 values are tabulated, rounded off to whole numbers, and highlighted based on the S/N-1 values (to the first decimal); S/N-1 ratios < 1 in pink, between 1 and 2 no highlight, between 2 and 5 in yellow, between 5 and 10 in light green, and > 10 in green. As depicted, the data from all three lots displayed a strong robust, dynamic response with increasing analyte that is suitable for quantitative measurements and distinguishing between subtle changes in concentration, if such information is required. Further, should it become necessary to extend the dynamic range to lower concentrations of analyte, it is feasible for 24 of the bead sets, but probably not for five bead sets (coconut-21, egg-26, milk-36, pine nut -39 and -42) which consistently displayed S/N-1 values of 2 or less for the first calibration standard (S1).

### Analysis of calibration standards by MLV participants

**Fig 1:**
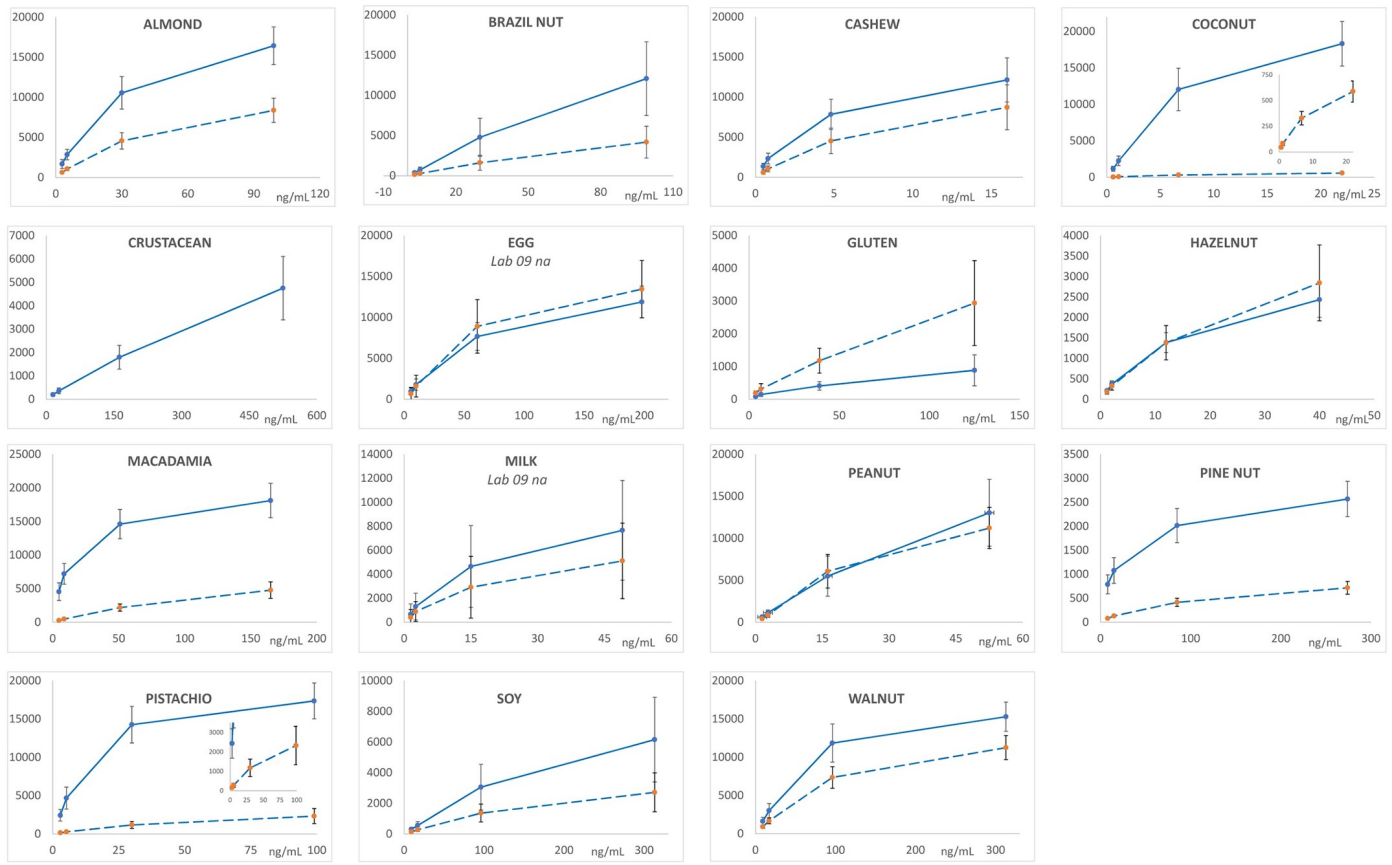
Calibration standards prepared in PBST. Plots of the calibration curves for each bead set, grouped by complementary pairs, for the average across all participating labs from an individual experiment using PBST as the solvent to prepare the calibration standards. The average MFI intensities were plotted as a function of the concentration of analyte in the calibration standards. The first numerical bead set of a complementary pair (i.e., almond-12, Brazil nut-14, cashew-18, coconut-20, egg-25, gluten-27, hazelnut-29, macadamia-33, milk-35, peanut-37, pine nut-39, pistachio-43, soy-45, and walnut-47) represented by solid lines (^_______^) and the complementary bead sets (almond-13, Brazil nut-15, cashew-19, coconut-21, egg-26, gluten-28, hazelnut-30, macadamia-34, milk-36, peanut-38, pine nut-42, pistachio-44, soy-46, and walnut-48) by dashed lines (- - - -). The MFI intensities averaged across the 11 laboratories were the values after subtracting the background (S0) with the error bars representing one standard deviation.

**Fig 2:**
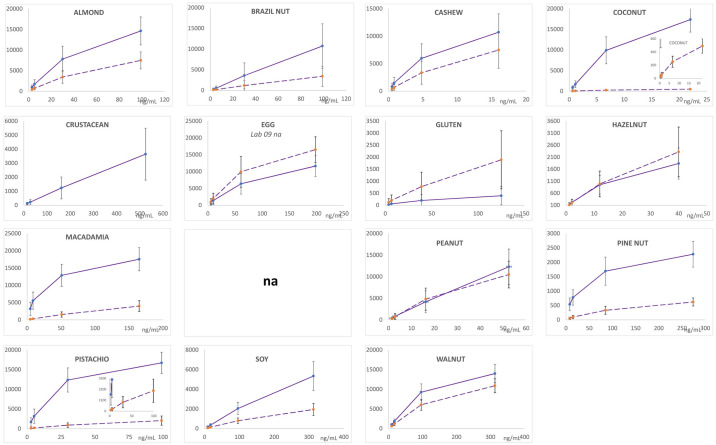
Calibration standards prepared in UD buffer. Similar to Fig 1 except UD buffer used as the solvent.

**Fig 3:**
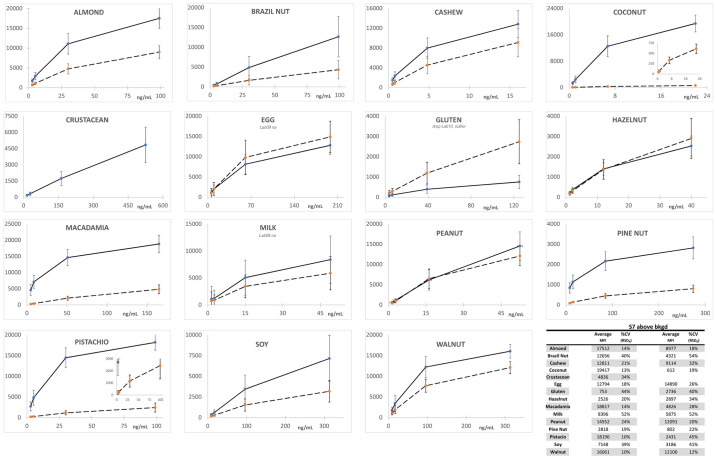
Average of the calibration standards from four experiments, three in PBST and one in UD buffer. Similar to Fig 1 except the average of four sets of calibration standards (3 PBST and 1 UD buffer, n = 11 labs* x 4 exp x triplicate analyses) generated by each participant was used to generate the final data plotted. The lower right corner tabulates the overall average MFI of S7 for each bead set along with the %CV (RSD_R_) values across the 11 labs. *The average data from Lab 05 represented only three (2 PBST and 1 UD buffer) experiments, Lab 09 did not collect any data for the egg (-25, -26) or milk (-35, -36) bead sets and the gluten data generated by Lab 10 were classified as outliers (exceptionally high background resulted in background adjusted MFI <0 for S1 and S2) and not included.

Figs 1 and 2 depict the average MFI intensities across the participating labs plotted versus the concentration of analyte in the calibration standards analyzed in a single experiment using PBST or UD Buffer, respectively, as the solvent milieu. The solid lines represent the first of the complementary bead sets (e.g., almond-12, Brazil nut-14, …) and the dashed lines represent the second (e.g., almond-13, Brazil nut-15, ….). The inserts in the coconut and pistachio plots expend the vertical scale to better illustrate the dynamic properties and MFI of the less intense bead sets (coconut-21 and pistachio-44). As previously observed, the use of UD Buffer did not significantly affect the performance properties of the bead sets with the target analytes. In all cases, the bead sets displayed excellent dynamic responses, with variances across the participating labs, represented by the error bars which equaled one standard deviation, that were acceptable. Fig 3 depicts the overall average MFI intensities across all 11 labs for the data averaged from four experiments (3 PBST and 1 UD buffer), typically performed on separate days, as a function of analyte concentration in the calibration standards. In the lower left corner, the average S7 MFI and associated %CV (RSD_R_) values are tabulated. In terms of MFI intensities, the RSD_R_ values ranged from 10–54%; ten (34%) were less than 20% and twelve (41%) displayed RSD_R_ values greater than 30%.

A better demonstration of within lab-consistency is illustrated in S1 Fig. For each of the 11 laboratories the average MFI intensities (above background), across four experiments, each typically performed on separate days, are plotted along with the error bars representative of the standard deviations across the 12 samples (four sets of triplicates, 9 samples for Lab 05) for each of the bead sets, with complementary antibodies on the same graph. Lab 01 displayed virtually no variance between the replicate samples for all 29 analytes. The other laboratories displayed different degrees of variance with the vast majority comparable. Only three of the 163 graphs (315 titration curves, 29x10 + 25), were calibration curves generated with unacceptable levels of variance, all three for milk-35 and -36 (Labs 06, 07, and 08). Four titration plots for soy, two for gluten, and one for walnut (14 titration curves) displayed greater than desirable levels of variance which might impact the ability to quantitatively distinguish between fine differences in analyte concentration. Interestingly, in these cases both complementary antibodies (e.g., bead sets milk-35 and milk-36) were problematic, never a single antibody bead set out of a complementary pair. This problematic behavior with both complementary antibody bead sets may indicate a problem with the preparation of the detector antibody cocktail, but a problem in the handling and preparation of the bead cocktails cannot be ruled out. Altogether, 94% of all calibration curves generated by the 11 participating laboratories were suitable despite the differences in analyst proficiency.

A major strength of the xMAP FADA is the incorporation of built-in controls that make it possible to detect false positives, false negatives, and even apply a quantitative measure of such as may be useful as in the case of some products that display low levels of ‘non-specific’ binding [7, 10]. The use of such is dependent on the relative reliability of the performance by the various bead sets and how such may fluctuate with analyst proficiency. This is examined in Table 6 in which the inter-bead set variability for each participant as a function increasing calibration standards (S1, S2, S5, S7), is itemized for each of the four experiments. Specifically, tabulated in Table 6 are the average %CV (RSD_r_) values for the triplicate analyses for each calibration standard, averaged across all 29 bead sets with associated standard deviations for each experiment. In addition, the overall averages across all participants are presented in the lower corner (RSD_R_). As expected, with increasing concentration, the %CV values decreased. Further, for each participant the variances across all bead sets and associated standard deviations were acceptable. Of the 86 entries for calibration standards S5 and S7, only 20 displayed RSD_r_ values ≥ 5% of which only five exceeded 10%. Labs 03, 04, and 07 had no RSD_r_ values above 4%, and Labs 01 and 10 had only one entry with an RSD_r_ of 6%, the remainder all ≤ 4%. Indeed, only one data entry displayed an RSD_r_ > 20%, Lab 02, experiment 2, calibration standard S7. As such, the built-in controls based on monitoring the various bead sets in the assay displayed acceptable levels of reliability irrespective of the proficiency of the analyst. In contrast, inter-lab MFI variability exceeded 25% with %CV (RSD_R_) values of ≥ 50%. The inter-lab %CV (RSD_R_) values provides a gauge for possible use when establishing identical standards for analyst performance.

**Table 6:** Average coefficient of variation across bead sets^a^.

		EXP 1 ^b^	EXP 2	EXP 3	EXP 4			EXP 1	EXP 2	EXP 3	EXP 4			EXP 1	EXP 2	EXP 3	EXP 4
**LAB 1** ^c^						**LAB 5**						**LAB 9**					
	**S1**	5 ± 6	2 ± 2	2 ± 2	2 ± 1		**S1**		30 ± 72	47 ± 46	535 ± 2194		**S1**	10 ± 9	15 ± 6	11 ± 10	10 ± 8
	**S2**	2 ± 2	2 ± 1	2 ± 1	2 ± 1		**S2**		14 ± 38	12 ± 114	27 ± 38		**S2**	9 ± 8	10 ± 4	5 ± 3	11 ± 7
	**S5**	2 ± 2	2 ± 1	6 ± 4	2 ± 1		**S5**		7 ± 8	8 ± 21	11 ± 27		**S5**	6 ± 3	4 ± 2	4 ± 3	6 ± 3
	**S7**	1 ± 1	1 ± 1	4 ± 3	1 ± 1		**S7**		7 ± 13	6 ± 15	7 ± 13		**S7**	12 ± 4	8 ± 4	8 ± 4	14 ± 5
**LAB 2**						**LAB 6**						**LAB 10**					
	**S1**	3 ± 3	3 ± 3	11 ± 13	5 ± 7		**S1**	8 ± 9	9 ± 99	4 ± 4	15 ± 36		**S1**	36 ± 155	7 ± 9	11 ± 31	26 ± 86
	**S2**	2 ± 2	2 ± 1	2 ± 1	3 ± 2		**S2**	9 ± 10	0 ± 26	3 ± 4	12 ± 14		**S2**	2 ± 11	5 ± 7	5 ± 7	4 ± 5
	**S5**	1 ± 1	2 ± 1	2 ± 1	1 ± 1		**S5**	5 ± 4	2 ± 23	2 ± 1	6 ± 6		**S5**	3 ± 8	2 ± 1	6 ± 4	2 ± 2
	**S7**	2 ± 3	85 ± 5	1 ± 1	1 ± 0		**S7**	3 ± 2	18 ± 22	2 ± 1	4 ± 4		**S7**	2 ± 2	3 ± 1	1 ± 1	2 ± 1
**LAB 3**						**LAB 7**						**LAB 11**					
	**S1**	3 ± 4	16 ± 113	9 ± 11	6 ± 18		**S1**	6 ± 8	‐31 ± 194	5 ± 9	5 ± 10		**S1**	7 ± 9	7 ± 11	6 ± 9	37 ± 388
	**S2**	5 ± 4	3 ± 2	7 ± 5	4 ± 35		**S2**	5 ± 10	17 ± 23	2 ± 4	5 ± 6		**S2**	4 ± 3	5 ± 5	5 ± 6	33 ± 99
	**S5**	2 ± 2	1 ± 1	2 ± 1	3 ± 4		**S5**	4 ± 7	3 ± 3	1 ± 2	3 ± 4		**S5**	1 ± 2	2 ± 1	2 ± 2	5 ± 15
	**S7**	7 ± 3	1 ± 1	1 ± 1	3 ± 1		**S7**	3 ± 5	3 ± 2	0 ± 4	2 ± 1		**S7**	2 ± 1	1 ± 1	2 ± 1	6 ± 9
**LAB 4**						**LAB 8**						**OVERALL** ^d^, ^e^					
	**S1**	3 ± 4	10 ± 18	5 ± 6	9 ± 18		**S1**	7 ± 9	16 ± 46	9 ± 7	9 ± 295		**S1**	55 ± 44	45 ± 35	59 ± 41	77 ± 38
	**S2**	2 ± 4	7 ± 8	3 ± 5	5 ± 6		**S2**	4 ± 9	12 ± 12	6 ± 9	4 ± 3		**S2**	48 ± 26	38 ± 19	50 ± 32	70 ± 33
	**S5**	3 ± 2	3 ± 1	2 ± 1	4 ± 2		**S5**	6 ± 7	2 ± 17	3 ± 2	2 ± 1		**S5**	35 ± 14	33 ± 17	37 ± 13	50 ± 24
	**S7**	1 ± 1	1 ± 1	1 ± 1	1 ± 1		**S7**	2 ± 1	3 ± 3	2 ± 3	4 ± 6		**S7**	27 ± 12	29 ± 15	30 ± 12	35 ± 19

^*a*^ Average %CV across all bead sets, for each calibration standard ran alongside the food samples. Outlier data for bead sets 27 & 28 not included for Lab 10, neither were data for bead sets 25, 26, 35, 36 not obtained by Lab 09. The Exp 1 data was based on only 10 laboratories due to the inability to ship the meat samples to an international participant (Lab 05).

^*b*^ Experiments 1, 2, 3, 4 refer to the analysis of Meat, Orange Juice, Baked Muffins, and Dark Chocolate samples, respectively.

^*C*^ Lab specific averages of %CV (RSD_r_) values and associated standard deviations for each bead set, determined for each calibration standard and food, each ran in triplicate. Provides a measure of intra‐lab consistency of intensities.

^*d*^ Overall %CV (RSD_R_) values across all 11 Laboratories: calculated from the average of the intensities for each bead set (minus background, S0) and averaged across the bead sets to determine an overall average and associated standard deviation to generate the Overall %CV (RSD_R_) values, for each calibration standard and food matrix. Provides a measure of inter‐lab consistency of intensities.

^*e*^ Averages across Lab Specific %CV values for S1, S2, S5, S7 generated %CV of 13±17, 8±13, 6±10, & 6±8% for Exp 1; 11±18, 10±10, 5±9, & 13±24% for Exp 2; 15±18, 9±13, 6±10, & 5±8% for Exp 3; and 61±151, 15±20, 8±14, & 7±10% for Exp 4, respectively.

Another measure of inter-lab variability is to compare the signal-to-noise above background data relative to the average across all 11 participating laboratories. This data is presented in Table 7. Tabulated are the averages across four experiments for each participant, for each calibration standard and bead set, rounded off to the first decimal place. To facilitate comparisons, values < 0.80 were highlighted in pink, between 0.80 and 1.20 in yellow, and > 1.20 in green. As indicated, Lab 01 had only 8% of the entries below 0.8 (80%) of the study average with most associated with pine nut-39, soy-45, -46, and walnut-47 and the vast majority (96 out of 116) above 1.2 (120% of the average). In contrast, Labs 05, 09, 10, and 11 generated responses consistently below the average. Labs 02, 03, 04, 06, 07, and 08 displayed average or better S/N-1 values. This trend is consistent with the xMAP proficiency of the analysts with seven labs at or above average and 4 labs below average.

**Table 7:** Calibration standards compared based upon the signal / noise, relative to the overall average across all laboratories^a^, ^b^.

	**ALMOND** ^b^		**BRAZIL NUT**		**CASHEW**		**COCONUT**		**CRUST**	**EGG**		**GLUTEN**		**HAZELNUT**	
	**12**	**13**	**14**	**15**	**18**	**19**	**20**	**21**	**22**	**25**	**26**	**27**	**28**	**29**	**30**
**LAB 01 / AV ALL LABS** ^c^															
**S1**	**3.7**	**1.9**	**2.7**	**3.0**	**2.3**	**2.4**	**1.6**	**1.7**	**2.6**	**7.8**	**6.2**	**5.9**	**5.9**	**1.9**	**1.9**
**S2**	**2.6**	**1.9**	**2.9**	**3.0**	**2.4**	**2.4**	**1.8**	**1.7**	**2.5**	**7.9**	**6.0**	**4.8**	**5.1**	**2.0**	**1.9**
**S5**	**2.4**	**1.9**	**2.8**	**3.0**	**2.0**	**2.3**	**1.5**	**1.6**	**2.1**	**7.4**	**4.7**	**4.3**	**4.7**	**1.6**	**1.8**
**S7**	**2.1**	**1.7**	**2.2**	**2.4**	**1.9**	**2.1**	**1.3**	**1.5**	**1.9**	**6.9**	**3.9**	**3.9**	**4.2**	**1.5**	**1.7**
**LAB 02 / AV ALL LABS**															
**S1**	**1.9**	**1.1**	**0.8**	**0.8**	**0.8**	**0.8**	**0.9**	**0.8**	**0.8**	**0.2**	**0.3**	**0.9**	**0.7**	**0.7**	**0.9**
**S2**	**1.2**	**1.1**	**0.9**	**0.8**	**0.8**	**0.8**	**0.9**	**0.8**	**0.8**	**0.2**	**0.3**	**1.0**	**1.0**	**0.7**	**0.9**
**S5**	**1.2**	**1.1**	**0.9**	**0.8**	**0.8**	**0.8**	**0.9**	**0.8**	**0.9**	**0.2**	**0.5**	**1.1**	**1.0**	**0.8**	**0.9**
**S7**	**1.3**	**1.2**	**0.9**	**0.9**	**0.8**	**0.8**	**0.9**	**0.9**	**0.9**	**0.3**	**0.5**	**1.2**	**1.1**	**0.8**	**0.9**
**LAB 03 / AV ALL LABS**															
**S1**	**2.4**	**1.5**	**1.2**	**1.1**	**1.3**	**1.0**	**1.7**	**1.6**	**0.8**	**0.4**	**0.7**	**0.3**	**0.5**	**1.3**	**1.1**
**S2**	**1.6**	**1.5**	**1.2**	**1.1**	**1.2**	**0.9**	**1.7**	**1.5**	**0.9**	**0.4**	**0.8**	**0.4**	**0.6**	**1.3**	**1.1**
**S5**	**1.3**	**1.3**	**1.2**	**1.0**	**1.0**	**0.8**	**1.2**	**1.3**	**1.1**	**0.4**	**0.8**	**0.4**	**0.7**	**1.0**	**1.0**
**S7**	**1.1**	**1.1**	**1.1**	**0.9**	**0.9**	**0.7**	**1.0**	**1.2**	**1.3**	**0.4**	**0.8**	**0.5**	**0.7**	**0.9**	**0.8**
**LAB 04 / AV ALL LABS**															
**S1**	**1.5**	**1.0**	**0.8**	**0.7**	**0.9**	**0.8**	**1.0**	**0.9**	**0.5**	**0.3**	**0.5**	**0.2**	**0.3**	**1.0**	**0.8**
**S2**	**1.0**	**1.0**	**0.8**	**0.7**	**0.9**	**0.8**	**1.0**	**0.9**	**0.5**	**0.3**	**0.4**	**0.2**	**0.3**	**1.0**	**0.8**
**S5**	**1.0**	**1.0**	**0.8**	**0.7**	**0.9**	**0.8**	**1.0**	**0.9**	**0.7**	**0.4**	**0.6**	**0.3**	**0.4**	**1.0**	**0.8**
**S7**	**1.0**	**1.0**	**0.9**	**0.8**	**0.9**	**0.8**	**0.9**	**1.0**	**0.9**	**0.4**	**0.6**	**0.3**	**0.4**	**0.9**	**0.8**
**LAB 05 / AV ALL LABS**															
**S1**	**0.1**	**0.2**	**0.7**	**0.1**	**0.4**	**0.3**	**0.5**	**0.3**	**0.5**	**0.2**	**0.4**	**0.1**	**0.3**	**0.5**	**0.2**
**S2**	**0.1**	**0.2**	**0.3**	**0.2**	**0.2**	**0.1**	**0.5**	**0.4**	**0.6**	**0.3**	**0.4**	**0.1**	**0.1**	**0.5**	**0.2**
**S5**	**0.1**	**0.2**	**0.2**	**0.2**	**0.2**	**0.1**	**0.6**	**0.5**	**0.7**	**0.4**	**0.8**	**0.1**	**0.1**	**0.6**	**0.2**
**S7**	**0.1**	**0.2**	**0.3**	**0.2**	**0.2**	**0.1**	**0.7**	**0.5**	**0.7**	**0.6**	**1.1**	**0.1**	**0.1**	**0.6**	**0.2**
**LAB 06 / AV ALL LABS**															
**S1**	**2.1**	**1.3**	**0.8**	**0.9**	**1.0**	**1.1**	**1.1**	**1.4**	**1.3**	**0.4**	**1.2**	**2.3**	**1.5**	**1.2**	**1.0**
**S2**	**1.4**	**1.3**	**0.9**	**0.9**	**1.1**	**1.1**	**1.1**	**1.3**	**1.3**	**0.3**	**0.8**	**2.4**	**1.7**	**1.2**	**1.0**
**S5**	**1.5**	**1.3**	**0.8**	**0.8**	**1.1**	**1.1**	**1.2**	**1.3**	**1.2**	**0.3**	**0.6**	**2.4**	**1.5**	**1.2**	**1.0**
**S7**	**1.7**	**1.5**	**1.0**	**0.9**	**1.2**	**1.2**	**1.3**	**1.3**	**1.1**	**0.3**	**0.7**	**2.4**	**1.4**	**1.2**	**1.1**
**LAB 07 / AV ALL LABS**															
**S1**	**1.1**	**0.9**	**1.2**	**1.2**	**1.2**	**1.1**	**1.4**	**1.3**	**1.9**	**0.2**	**0.5**	**0.5**	**0.7**	**1.2**	**1.2**
**S2**	**0.7**	**0.8**	**1.2**	**1.2**	**1.3**	**1.1**	**1.4**	**1.3**	**1.9**	**0.2**	**0.6**	**0.6**	**0.8**	**1.2**	**1.2**
**S5**	**0.8**	**0.9**	**1.4**	**1.2**	**1.4**	**1.2**	**1.5**	**1.2**	**1.8**	**0.3**	**0.6**	**0.6**	**0.8**	**1.3**	**1.2**
**S7**	**0.8**	**0.9**	**1.5**	**1.3**	**1.3**	**1.2**	**1.5**	**1.2**	**1.6**	**0.3**	**0.6**	**0.6**	**0.9**	**1.3**	**1.2**
**LAB 08 / AV ALL LABS**															
**S1**	**0.6**	**0.6**	**1.0**	**1.4**	**1.0**	**1.5**	**0.9**	**0.9**	**0.8**	**0.2**	**0.3**	**1.1**	**0.7**	**0.9**	**1.5**
**S2**	**0.4**	**0.6**	**1.1**	**1.4**	**1.1**	**1.6**	**0.8**	**1.0**	**0.8**	**0.1**	**0.2**	**1.3**	**0.8**	**0.9**	**1.6**
**S5**	**0.5**	**0.7**	**1.1**	**1.5 1.2 1.7**	**1.2**	**1.7**	**1.0**	**1.1**	**0.9**	**0.3**	**0.6**	**1.4**	**1.0**	**1.1**	**1.7**
**S7**	**0.6**	**0.8**	**1.2 1.7 1.2 1.7**	**1.7**	**1.2**	**1.7**	**1.0**	**1.1**	**1.0**	**0.4**	**0.7**	**1.4**	**1.2**	**1.2**	**1.8**
**LAB 09 / AV ALL LABS**															
**S1**	**0.7**	**0.7**	**0.4**	**0.5**	**0.6**	**0.7**	**0.5**	**0.7**	**0.6**	**na** ^c^		**0.2**	**0.3**	**0.9**	**0.7**
**S2**	**0.4**	**0.7**	**0.4**	**0.4**	**0.6**	**0.7**	**0.4**	**0.6**	**0.5**			**0.1**	**0.3**	**0.9**	**0.6**
**S5**	**0.5**	**0.7**	**0.4**	**0.4**	**0.8**	**0.7**	**0.6**	**0.7**	**0.5**			**0.2**	**0.4**	**1.0**	**0.7**
**S7**	**0.7**	**0.9**	**0.5**	**0.5**	**1.0**	**0.9**	**0.9**	**0.8**	**0.5**			**0.2**	**0.4**	**1.1**	**0.7**
**LAB 10 / AV ALL LABS**															
**S1**	**1.1**	**0.9**	**0.5**	**0.5**	**0.6**	**0.6**	**0.7**	**0.6**	**0.5**	**0.0**	**‐0.4**	**‐0.4**	**‐0.2**	**0.5**	**0.6**
**S2**	**0.8**	**0.9**	**0.6**	**0.6**	**0.7**	**0.6**	**0.7**	**0.7**	**0.6**	**0.1**	**0.1**	**‐0.1**	**0.0**	**0.7**	**0.7**
**S5**	**0.7**	**0.9**	**0.6**	**0.6**	**0.7**	**0.6**	**0.7**	**0.7**	**0.6**	**0.1**	**0.3**	**0.0**	**0.1**	**0.7**	**0.7**
**S7**	**0.7**	**0.9**	**0.6**	**0.6**	**0.6**	**0.5**	**0.7**	**0.7**	**0.6**	**0.2**	**0.4**	**0.0**	**0.1**	**0.7**	**0.7**
**LAB 11 / AV ALL LABS**															
**S1**	**1.3**	**0.9**	**0.8**	**0.8**	**0.9**	**0.9**	**0.7**	**0.8**	**0.6**	**0.2**	**0.5**	**0.1**	**0.2**	**0.8**	**1.0**
**S2**	**0.8**	**0.9**	**0.8**	**0.8**	**0.8**	**0.8**	**0.7**	**0.8**	**0.5**	**0.2**	**0.4**	**0.1**	**0.3**	**0.7**	**0.9**
**S5**	**0.9**	**1.0**	**0.9**	**0.8**	**0.9**	**0.9**	**0.8**	**0.8**	**0.5**	**0.2**	**0.5**	**0.1**	**0.4**	**0.8**	**1.0**
**S7**	**0.9**	**1.0**	**0.9**	**0.8**	**0.9**	**0.9**	**0.8**	**0.8**	**0.5**	**0.3**	**0.6**	**0.2**	**0.4**	**0.8**	**1.0**
**AVERAGE S/N‐1 ACROSS LABS** ^d^															
**S1**	**16**	**10**	**4**	**3**	**8**	**14**	**9**	**1**	**2**	**1**	**0**	**0**	**0**	**1**	**4**
**S2**	**27**	**17**	**8**	**5**	**14**	**24**	**17**	**1**	**3**	**1**	**0**	**1**	**0**	**2**	**6**
**S5**	**103**	**72**	**52**	**31**	**48**	**106**	**89**	**6**	**15**	**5**	**1**	**2**	**2**	**6**	**27**
**S7**	**161**	**137**	**135**	**83**	**77**	**212**	**139**	**11**	**40**	**6**	**2**	**4**	**4**	**11**	**58**
**STDEV OF S/N‐1 DATA ACROSS ALL LABS** ^d^															
**S1**	**10.3**	**4.4**	**2.8**	**2.3**	**4.2**	**7.9**	**3.8**	**0.4**	**1.1**	**1.8**	**0.3**	**0.7**	**0.5**	**0.4**	**1.6**
**S2**	**18.6**	**7.7**	**5.4**	**4.1**	**7.8**	**14.2**	**7.8**	**0.6**	**1.8**	**3.3**	**0.5**	**0.9**	**0.6**	**0.6**	**2.8**
**S5**	**62.7**	**31.4**	**36.4**	**23.7**	**22.3**	**62.5**	**29.9**	**2.0**	**7.7**	**10.4**	**1.6**	**2.6**	**2.3**	**1.8**	**12.3**
**S7**	**88.9**	**52.5**	**69.1**	**51.3**	**33.4**	**115.9**	**36.8**	**3.2**	**18.1**	**13.3**	**1.7**	**4.3**	**4.5**	**3.2**	**26.8**
**Bkgd** ^e^	**65**	**53**	**81**	**51**	**130**	**36**	**122**	**48**	**105**	**398**	**3634**	**102**	**292**	**207**	**48**
**SD**	357	71	31	18	150	71	41	17	41	2192	3571	470	954	71	20
	**MACADAMIA**		**MILK**		**PEANUT**		**PINE NUT**		**PISTACHIO**		**SOY**		**WALNUT**		
	**33**	**34**	**35**	**36**	**37**	**38**	**39**	**42**	**43**	**44**	**45**	**46**	**47**	**48**
**LAB 01 / AV ALL LABS** ^c^															
**S1**	**1.7**	**1.4**	**3.0**	**1.8**	**2.1**	**1.9**	**0.9**	**1.4**	**2.1**	**1.8**	**0.7**	**0.9**	**0.7**	**1.1**	
**S2**	**1.5**	**1.4**	**3.3**	**2.0**	**2.0**	**1.9**	**0.9**	**1.4**	**2.0**	**2.1**	**0.6**	**0.9**	**0.7**	**1.1**	
**S5**	**1.2**	**1.4**	**3.6**	**2.1**	**2.1**	**1.9**	**0.8**	**1.3**	**1.5**	**2.1**	**0.5**	**0.7**	**0.9**	**1.2**	
**S7**	**1.1**	**1.5**	**3.1**	**1.9**	**1.7**	**1.5**	**0.7**	**1.1**	**1.4**	**2.1**	**0.5**	**0.6**	**0.9**	**1.2**	
**LAB 02 / AV ALL LABS**															
**S1**	**1.2**	**1.0**	**1.5**	**1.5**	**1.2**	**1.0**	**1.0**	**1.0**	**0.9**	**0.7**	**1.4**	**1.2**	**0.9**	**1.0**	
**S2**	**1.2**	**1.0**	**1.7**	**1.8**	**1.2**	**0.9**	**1.0**	**1.0**	**0.9**	**0.8**	**1.4**	**1.1**	**1.0**	**1.0**	
**S5**	**1.2**	**1.0**	**2.0**	**2.3**	**1.2**	**1.0**	**1.0**	**1.0**	**0.9**	**0.8**	**1.3**	**1.0**	**0.9**	**1.0**	
**S7**	**1.1**	**1.1**	**2.0**	**2.2**	**1.2**	**1.0**	**1.1**	**1.1**	**0.9**	**0.8**	**1.2**	**1.1**	**0.8**	**0.9**	
**LAB 03 / AV ALL LABS**															
**S1**	**1.4**	**1.3**	**1.1**	**1.5**	**1.4**	**1.6**	**1.4**	**1.4**	**1.5**	**0.8**	**1.7**	**1.6**	**1.4**	**1.8**	
**S2**	**1.2**	**1.3**	**1.2**	**1.7**	**1.4**	**1.6**	**1.3**	**1.3**	**1.4**	**0.9**	**1.7**	**1.7**	**1.8**	**2.3**	
**S5**	**1.0**	**1.1**	**1.0**	**1.6**	**1.5**	**1.2**	**1.2**	**1.2**	**0.9**	**0.7**	**1.7**	**1.9**	**0.8**	**1.3**	
**S7**	**1.0**	**1.0**	**0.9**	**1.3**	**1.1**	**0.9**	**1.1**	**1.1**	**0.9**	**0.6**	**1.2**	**1.6**	**0.7**	**1.0**	
**LAB 04 / AV ALL LABS**															
**S1**	**0.9**	**1.0**	**1.1**	**1.1**	**0.9**	**1.1**	**1.2**	**1.0**	**0.9**	**0.6**	**1.0**	**1.1**	**0.9**	**1.1**	
**S2**	**0.9**	**1.0**	**1.2**	**1.2**	**0.9**	**1.1**	**1.2**	**0.9**	**0.9**	**0.7**	**1.0**	**1.1**	**0.8**	**1.1**	
**S5**	**0.8**	**1.0**	**1.4**	**1.4**	**0.8**	**1.1**	**1.1**	**1.0**	**1.0**	**0.7**	**1.1**	**1.1**	**0.8**	**1.1**	
**S7**	**0.9**	**0.9**	**1.4**	**1.4**	**0.8**	**1.0**	**1.1**	**1.0**	**1.0**	**0.7**	**1.1**	**1.2**	**0.7**	**1.0**	
**LAB 05 / AV ALL LABS**															
**S1**	**0.6**	**0.5**	**0.0**	**‐0.1**	**0.0**	**0.2**	**0.1**	**0.2**	**0.3**	**1.7**	**0.6**	**0.4**	**0.7**	**0.5**	
**S2**	**0.6**	**0.5**	**0.0**	**0.1**	**0.2**	**0.4**	**0.1**	**0.3**	**0.2**	**0.3**	**0.6**	**0.4**	**0.6**	**0.5**	
**S5**	**0.7**	**0.5**	**0.0**	**0.0**	**0.1**	**0.3**	**0.2**	**0.4**	**0.2**	**0.3**	**0.7**	**0.5**	**0.8**	**0.6**	
**S7**	**0.7**	**0.6**	**0.0**	**0.0**	**0.2**	**0.3**	**0.2**	**0.4**	**0.2**	**0.3**	**0.9**	**0.6**	**0.8**	**0.6**	
**LAB 06 / AV ALL LABS**															
**S1**	**1.1**	**1.3**	**0.7**	**1.2**	**1.3**	**1.2**	**1.2**	**1.6**	**1.2**	**1.1**	**1.8**	**1.5**	**3.6**	**1.0**	
**S2**	**1.2**	**1.3**	**1.0**	**2.0**	**1.3**	**1.2**	**1.2**	**1.6**	**1.3**	**1.3**	**1.8**	**1.5**	**3.3**	**0.9**	
**S5**	**1.3**	**1.3**	**0.5**	**1.2**	**1.2**	**1.2**	**1.2**	**1.6**	**1.5**	**1.2**	**1.7**	**1.5**	**3.7**	**1.0**	
**S7**	**1.4**	**1.3**	**0.9**	**1.8**	**1.6**	**1.5**	**1.2**	**1.7**	**1.6**	**1.2**	**1.9**	**1.5**	**3.8**	**1.1**	
**LAB 07 / AV ALL LABS**															
**S1**	**1.3**	**1.0**	**0.0**	**0.0**	**0.9**	**1.2**	**1.4**	**1.2**	**1.4**	**1.1**	**0.7**	**0.7**	**0.6**	**0.9**	
**S2**	**1.4**	**1.0**	**0.0**	**0.0**	**0.9**	**1.2**	**1.5**	**1.2**	**1.5**	**1.2**	**0.6**	**0.7**	**0.6**	**0.9**	
**S5**	**1.3**	**1.1**	**0.0**	**0.0**	**1.0**	**1.4**	**1.5**	**1.1**	**1.5**	**1.2**	**0.5**	**0.6**	**0.8**	**1.1**	
**S7**	**1.3**	**1.1**	**0.0**	**0.0**	**1.1**	**1.4**	**1.4**	**1.1**	**1.5**	**1.2**	**0.5**	**0.6**	**0.9**	**1.2**	
**LAB 08 / AV ALL LABS**															
**S1**	**0.9**	**1.3**	**‐0.1**	**‐0.2**	**1.4**	**0.8**	**1.3**	**1.2**	**0.8**	**1.3**	**0.9**	**1.4**	**0.7**	**1.2**	
**S2**	**1.0**	**1.3**	**‐0.1**	**‐0.2**	**1.3**	**0.8**	**1.1**	**1.2**	**0.9**	**1.4**	**0.9**	**1.4**	**0.7**	**1.1**	
**S5**	**1.1**	**1.4**	**0.0**	**0.0**	**1.3**	**1.0**	**1.3**	**1.2**	**1.1**	**1.6**	**1.0**	**1.4**	**0.8**	**1.4**	
**S7**	**1.1**	**1.5**	**0.0**	**0.1**	**1.5**	**1.1**	**1.4**	**1.3**	**1.1**	**1.7**	**1.0**	**1.5**	**0.8**	**1.4**	
**LAB 09 / AV ALL LABS**															
**S1**	**0.4**	**0.7**	**na**		**0.5**	**0.5**	**1.0**	**0.8**	**0.5**	**0.6**	**0.6**	**0.5**	**0.3**	**0.5**	
**S2**	**0.5**	**0.7**			**0.5**	**0.4**	**1.0**	**0.7**	**0.5**	**0.7**	**0.6**	**0.6**	**0.3**	**0.4**	
**S5**	**0.7**	**0.6**			**0.4**	**0.5**	**1.1**	**0.7**	**0.9**	**0.8**	**0.6**	**0.6**	**0.5**	**0.6**	
**S7**	**0.7**	**0.6**			**0.5**	**0.8**	**1.1**	**0.8**	**1.1**	**0.9**	**0.7**	**0.7**	**0.7**	**0.8**	
**LAB 10 / AV ALL LABS**															
**S1**	**0.7**	**0.6**	**1.8**	**2.3**	**0.7**	**0.7**	**0.6**	**0.6**	**0.7**	**0.4**	**0.6**	**0.7**	**0.5**	**0.8**	
**S2**	**0.7**	**0.7**	**0.6**	**0.7**	**0.6**	**0.7**	**0.6**	**0.8**	**0.7**	**0.5**	**0.7**	**0.7**	**0.5**	**0.8**	
**S5**	**0.8**	**0.7**	**0.2**	**0.2**	**0.6**	**0.7**	**0.6**	**0.8**	**0.7**	**0.5**	**0.7**	**0.7**	**0.5**	**0.8**	
**S7**	**0.8**	**0.6**	**0.2**	**0.2**	**0.7**	**0.7**	**0.6**	**0.8**	**0.7**	**0.5**	**0.8**	**0.8**	**0.5**	**0.7**	
**LAB 11 / AV ALL LABS**															
**S1**	**0.9**	**0.9**	**1.0**	**0.7**	**0.6**	**0.8**	**1.0**	**0.6**	**0.8**	**0.8**	**1.1**	**0.9**	**0.6**	**1.0**	
**S2**	**0.9**	**0.9**	**1.1**	**0.8**	**0.6**	**0.7**	**1.0**	**0.6**	**0.8**	**0.9**	**1.1**	**0.9**	**0.6**	**1.0**	
**S5**	**0.9**	**0.8**	**1.3**	**1.1**	**0.7**	**0.8**	**1.0**	**0.7**	**0.9**	**1.0**	**1.2**	**0.9**	**0.6**	**1.0**	
**S7**	**0.9**	**0.8**	**1.5**	**1.1**	**0.8**	**0.8**	**1.1**	**0.7**	**0.9**	**1.0**	**1.1**	**0.9**	**0.5**	**1.0**	
**AVERAGE S/N‐1 ACROSS LABS** ^d^															
**S1**	**36**	**3**	**2**	**1**	**4**	**4**	**2**	**1**	**24**	**3**	**3**	**2**	**5**	**7**	
**S2**	**57**	**6**	**4**	**2**	**7**	**8**	**3**	**2**	**45**	**4**	**5**	**3**	**10**	**14**	
**S5**	**117**	**30**	**19**	**9**	**39**	**58**	**6**	**6**	**137**	**19**	**30**	**15**	**37**	**56**	
**S7**	**150**	**70**	**32**	**16**	**93**	**109**	**8**	**10**	**172**	**40**	**63**	**32**	**50**	**89**	
**STDEV OF S/N‐1 DATA ACROSS ALL LABS** ^d^															
**S1**	**13.8**	**1.1**	**2.0**	**1.0**	**2.2**	**1.9**	**0.9**	**0.5**	**12.7**	**1.3**	**1.3**	**0.7**	**4.7**	**2.7**	
**S2**	**18.1**	**1.8**	**3.9**	**1.8**	**3.6**	**3.7**	**1.1**	**0.7**	**23.2**	**2.2**	**2.4**	**1.2**	**8.4**	**6.9**	
**S5**	**28.1**	**9.0**	**22.2**	**8.0**	**21.7**	**26.6**	**2.1**	**2.0**	**55.2**	**9.7**	**12.9**	**6.8**	**33.4**	**14.5**	
**S7**	**35.7**	**23.1**	**32.7**	**13.6**	**42.9**	**39.4**	**2.7**	**3.6**	**66.9**	**21.2**	**24.8**	**12.5**	**47.2**	**20.4**	
**Bkgd** ^e^	**131**	**67**	**126**	**293**	**134**	**101**	**511**	**78**	**89**	**59**	**106**	**75**	**411**	**128**	
**SD**	31	23	3135	2600	215	75	298	34	115	24	33	38	154	36	

^*a*^ Comparisons between Signal /Noise above background (S0, S/N ‐ 1) by each laboratory versus the average S/N‐1 calculated across all 11 participating laboratories. The S/N‐1 ratios were calculated for each laboratory based on the average MFI (above background) for each bead set, for calibration standards S1, S2, S5, S7 as observed in four separate experiments (three for Lab 05); S3, S4, S6 were not included in the experiments.

^*b*^ Comparison ratios < 0.80 highlighted in pink; 0.80 < ratio < 1.20 highlighted in yellow; ratios > 1.20 highlighted in green. Ratios displayed rounded‐off to the first decimal but highlight colors based on the values to the second decimal place; 0.79 and 0.81 would both be displayed as 0.8 but highlighted differently.

^*c*^ Ratio between the S/N‐1 by the specified lab versus the overall average S/N‐1 calculated across all labs. 'na' refers to data not available.

^*d*^ Average S/N‐1 across all labs rounded‐off to whole units. Not included in the overall averages are Gluten ‐27 & ‐28 data by Lab 10 (outlier); Egg ‐25 &‐26 and Milk‐35 & ‐36 data by Lab 09 not available. Lab 05 data based on only 3 experiments.

^*e*^ Average background MFI across all 11 participating laboratories, each an average of the data gathered from four (three for Lab 05) experiments, with each sample analyzed in triplicate. Standard deviation (SD) calculated across the results by each laboratory.

To better evaluate the quality of the data generated by the various participants and its analytical utility entailed comparing the signal-to-noise, above background (S/N-1) across the dynamic range as defined by the calibration standards S1 and S7 for each bead set. This data is presented in Table 8. Included in the table are also the results associated with Lots 1 and 3 along with the average across the participating labs, *sans* outliers indicated by either red or pink highlight (Lab 10 egg-25, -26, gluten -27, -28; Lab 05 milk -35, -36, peanut -37, -38; and Lab 08 milk -35, -36). Otherwise, 18 of the 29 bead sets displayed ≥ 10-fold increases across the dynamic range, with three (Brazil nut-15, macadamia-34, and peanut-38) displaying > 20-fold increases. Only macadamia-33 and pine nut-39 displayed ratios of 3; though not large, still adequate for quantitatively distinguishing changes in analyte concentration.

**Table 8:** Comparison signal / noise, above background, spanning the calibration standards [(S/N‐1)_S7_ / (S/N‐1)_S1_]^a^.

LOT ^b^	Analyst ^c^	ALMOND ^d^		BRAZIL NUT		CASHEW		COCONUT		CRUST	EGG		GLUTEN		HAZELNUT	
		12	13	14	15	18	19	20	21	22	25	26	27	28	29	30
**Lot 1**	**na**	**6**	**10**	**41**	**51**	**7**	**23**	**11**	**13**	**30**	**na**	**na**	**7**	**7**	**17**	**26**
**Lot 3**	**na**	**6**	**10**	**15**	**15**	**7**	**14**	**10**	**11**	**16**	**9**	**9**	**6**	**8**	**12**	**15**
**Lot 5**	***adj av***	**11**	**15**	**32**	**30**	**9**	**15**	**17**	**14**	**28**	**12**	**16**	**11**	**15**	**12**	**17**
	***stdev***	**3**	**3**	**8**	**5**	**2**	**4**	**5**	**4**	**8**	**4**	**9**	**3**	**5**	**2**	**2**
	**%CV**	**24**	**17**	**24**	**18**	**26**	**26**	**32**	**27**	**28**	**36**	**52**	**26**	**36**	**17**	**14**
**Lot 5**	**Lab 01** ^e^	**9**	**12**	**24**	**22**	**8**	**13**	**12**	**11**	**18**	**8**	**7**	**6**	**8**	**9**	**15**
**Lot 5**	**Lab 02**	**11**	**15**	**31**	**30**	**10**	**16**	**14**	**16**	**28**	**14**	**19**	**12**	**18**	**12**	**16**
**Lot 5**	**Lab 03**	**8**	**11**	**28**	**24**	**7**	**12**	**9**	**9**	**40**	**8**	**13**	**14**	**18**	**8**	**11**
**Lot 5**	**Lab 04**	**10**	**14**	**33**	**31**	**9**	**15**	**15**	**15**	**42**	**11**	**15**	**14**	**18**	**11**	**17**
**Lot 5**	**Lab 05**	**8**	**12**	**13**	**42**	**5**	**5**	**20**	**24**	**33**	**20**	**34**	**8**	**4**	**13**	**20**
**Lot 5**	**Lab 06**	**13**	**16**	**35**	**27**	**11**	**17**	**18**	**13**	**21**	**7**	**7**	**9**	**11**	**12**	**17**
**Lot 5**	**Lab 07**	**11**	**15**	**39**	**30**	**10**	**16**	**16**	**12**	**22**	**12**	**14**	**12**	**15**	**13**	**17**
**Lot 5**	**Lab 08**	**14**	**19**	**36**	**34**	**11**	**17**	**19**	**15**	**34**	**16**	**25**	**12**	**19**	**15**	**20**
**Lot 5**	**Lab 09**	**16**	**17**	**37**	**29**	**14**	**20**	**30**	**14**	**23**	**na**	**na**	**14**	**14**	**13**	**18**
**Lot 5**	**Lab 10**	**10**	**14**	**39**	**34**	**9**	**15**	**14**	**15**	**28**	**80**	**-10**	**-1**	**-5**	**15**	**18**
**Lot 5**	**Lab 11**	**11**	**14**	**32**	**28**	**10**	**15**	**16**	**13**	**24**	**11**	**14**	**14**	**21**	**12**	**17**
**LOT**	**Analyst**	**MACADAMIA**		**MILK**		**PEANUT**		**PINE NUT**		**PISTACHIO**		**SOY**		**WALNUT**		
		**33**	**34**	**35**	**36**	**37**	**38**	**39**	**42**	**43**	**44**	**45**	**46**	**47**	**48**	
**Lot 1**	**na**	**4**	**25**	**na**	**na**	**13**	**16**	**4**	**12**	**9**	**30**	**8**	**8**	**9**	**13**	
**Lot 3**	**na**	**3**	**19**	**5**	**6**	**13**	**15**	**3**	**11**	**5**	**18**	**16**	**10**	**16**	**16**	
**Lot 5**	***adj av***	**4**	**21**	**14**	**13**	**23**	**30**	**4**	**10**	**8**	**15**	**22**	**21**	**10**	**13**	
	***stdev***	**1**	**3**	**7**	**7**	**4**	**8**	**1**	**3**	**3**	**5**	**5**	**6**	**4**	**3**	
	**%CV**	**29**	**17**	**53**	**51**	**16**	**27**	**35**	**33**	**43**	**33**	**22**	**27**	**40**	**26**	
**Lot 5**	**Lab 01**	**3**	**22**	**16**	**14**	**18**	**22**	**3**	**7**	**5**	**16**	**16**	**13**	**11**	**13**	
**Lot 5**	**Lab 02**	**4**	**21**	**19**	**19**	**24**	**28**	**4**	**11**	**7**	**15**	**19**	**18**	**8**	**11**	
**Lot 5**	**Lab 03**	**3**	**15**	**12**	**11**	**17**	**16**	**3**	**7**	**4**	**10**	**15**	**19**	**5**	**7**	
**Lot 5**	**Lab 04**	**4**	**18**	**19**	**17**	**22**	**27**	**3**	**9**	**7**	**17**	**23**	**21**	**8**	**11**	
**Lot 5**	**Lab 05**	**5**	**27**	-412	-5	106	55	**7**	**20**	**5**	**2**	**30**	**34**	**11**	**14**	
**Lot 5**	**Lab 06**	**5**	**21**	**20**	**20**	**27**	**35**	**3**	**10**	**9**	**16**	**23**	**20**	**10**	**13**	
**Lot 5**	**Lab 07**	**4**	**23**	**5**	**6**	**28**	**33**	**3**	**8**	**8**	**15**	**16**	**16**	**13**	**16**	
**Lot 5**	**Lab 08**	**5**	**23**	**‐4**	**‐4**	**24**	**37**	**4**	**10**	**9**	**19**	**23**	**21**	**10**	**14**	
**Lot 5**	**Lab 09**	**8**	**18**	**na**	**na**	**23**	**46**	**3**	**9**	**16**	**20**	**27**	**25**	**20**	**20**	
**Lot 5**	**Lab 10**	**4**	**21**	**1**	**1**	**23**	**29**	**3**	**12**	**7**	**17**	**27**	**23**	**9**	**11**	
**Lot 5**	**Lab 11**	**4**	**17**	**21**	**20**	**27**	**30**	**3**	**10**	**8**	**17**	**21**	**19**	**8**	**12**	

^*a*^ Ratio between the Signal/Noise (above background, S/N‐1) spanning the dynamic range of the calibration standards from S1 to S7 [(S/N‐1)_S7_ / (S/N-1)_S1_].

^*b*^ Lot production number, Lot 1 first, Lot 3 used for single lab validation, Lot 5 used for multi‐laboratory validation (MLV). All analyses within 6 months of production.

^*c*^ Analyst information: 'na' not applicable, 'adj av'‐ adjusted average is the average across the laboratories participating in the MLV (Labs 01 ‐ 11, below) omitting outlier data indicative of operational problems (highlighted in red and pink). 'stdev' and %CV' are the standard deviations and percent coefficient of variation (%CV) associated with the data used to calculate the adjusted averages. Lab 01 ‐ 11 refer to the participating laboratories in the MLV.

^*d*^ Target analyte and associated bead set number.

^*e*^ MLV laboratory (Labs 01 ‐ 11) data are the average across 4 experiments (3 for Lab 05) each triplicate analyses of the calibration standards. Outlier data highlighted in orange (negative indicative of a serious error) or pink (indicative of exceptionally high values). Exceptionally low ratios (< 2) highlighted in yellow, but included in the adjusted average. 'na' refers to not applicable, Lab 09 did not collect data for bead sets ‐25, ‐26, ‐35, and ‐36.

Overall, all participants generated calibration curves suitable for analyte quantitation. These results with the calibration standards indicate an acceptable level of proficiency in preparing and handling the reagents, performing the xMAP assay (post sample extraction), and operating the instrumentation.

### Food samples

The food samples provide a measure of the inter-laboratory reliability of the assay upon including additional steps of sample preparation and extraction. To better gauge this variance, four types of food samples were chosen to reflect different physical-chemical properties; specifically, sausage-hot dog meat (high in fat and chemical preservatives), orange juice (acidic and contains a reductant, ascorbic acid), baked muffins (heating in a moist environment that undergoes dehydration), and dark chocolate (high in polyphenols and alkaloids). The concentrations of analyte incurred into the foods were chosen to span the dynamic range of the assay and reflect the lower concentration range of interest when analyzing regulatory samples. The coded food samples were prepared by incurring 25 μL of a mixture of the allergenic foods per gram food to generate final concentrations of 0, 1, 2.5, 10, 25, and 100 ppm of each allergenic food in the food sample. By incorporating an optional dilution step, 10-fold for the PBST extracts and 5-fold for the UD buffer extracts (net dilution of the food 200-fold), the analyses served to both span the dynamic range of the assay and demonstrate the ability to analyze samples containing one tenth the incurred concentrations. The applicability of the MLV data to represent performance at these lower concentrations is partly based on the minimal change in matrix carry-over due to the extensive dilution with buffer during extraction.

The food samples provided to the participating labs were:

Ground pure beef sausage-hot dog containing in equal mass proportion Egg, Gluten, Milk, and Soy.
Orange Juice containing equal mass proportions of Almond, Milk, and Soy.
Baked Muffin incurred with equal mass proportions of Coconut, Egg, Gluten, Milk, and Walnut.
Dark Chocolate incurred in equal mass proportions with Hazelnut, Milk, and Peanut.

These samples were extracted using UD buffer (contains 2.5% m/v NFDM) and therefore not analyzed for milk. The first and most important feature of an analytical method is its ability to qualitatively detect the analyte. Since presumptive false positives undergo a secondary confirmation this is not a significant problem. Secondary confirmation of presumptive positives with the xMAP FADA is derived from concurrence between complementary antibody bead sets, the presence of antibody bead sets that do not recognize the target analyte (e.g., crustacean-22), ratio analysis, and multi-antibody profiling. Thus, the major concern is not false positives but false negatives. The xMAP FADA incorporates multiple controls to prevent false negatives due to technical aspects of the assay (e.g., the AssayChex^™^ bead sets). However, when working with food samples false negatives (and positives) are often due to variances in the data as may be associated with analyst proficiency and/or complexity of the food matrix.

**Fig 4:**
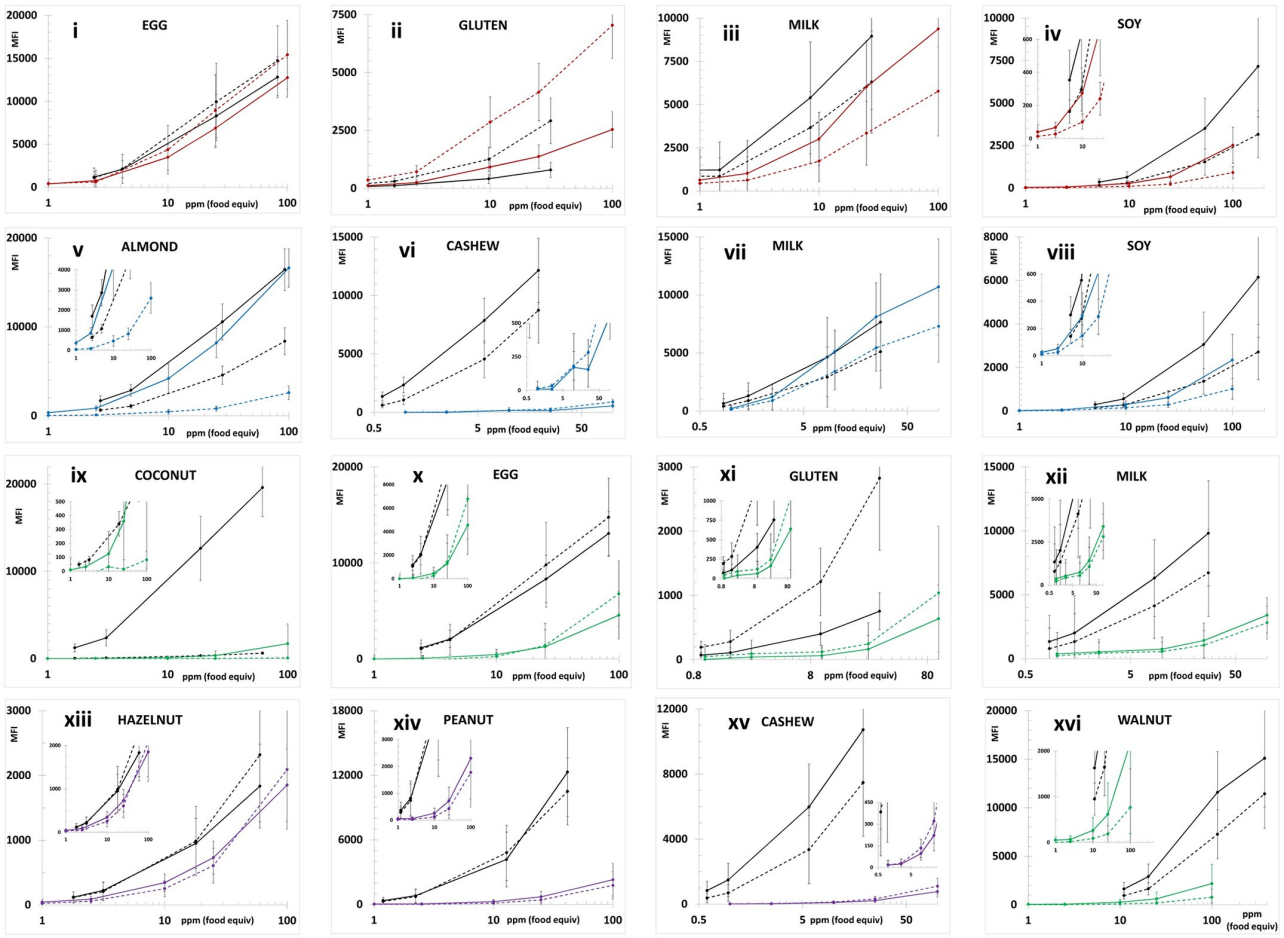
Average MFI across all 11 laboratories of bead sets generating MFI with incurred food samples. Plotted are the average of concurring positive bead set analyses, each performed in triplicate across all 11 laboratories. The colored lines are the data derived from the food samples (**red**—meat, **blue**–orange juice, **green**–baked muffins, and **purple**–dark chocolate) and the **black** lines are the calibration standards ran concurrently. Solid lines indicate the lower numerical bead set (e.g., egg-25) and the dashed lines the higher (e.g., egg-26) of complementary pairs. Graphs i, ii, iii, iv depict the egg, gluten, milk, and soy bead set analyses of the meat samples; Graphs v, vi, vii, viii depict the almond, cashew, milk, and soy bead set analyses of the orange juice samples; Graphs ix, x, xi, xii, xvi depict the coconut, egg, gluten, milk, and walnut bead set analyses of the baked muffin samples; Graphs xiii, xiv, xv depict the hazelnut, peanut, and cashew bead set analyses of dark chocolate. The two cashew results are included despite the responses being ‘classical’ cross-reactivity with very low measurable MFI and the ratios inconsistent with cashew. The meat data entailed only the results from 10 laboratories.

**Fig 5:**
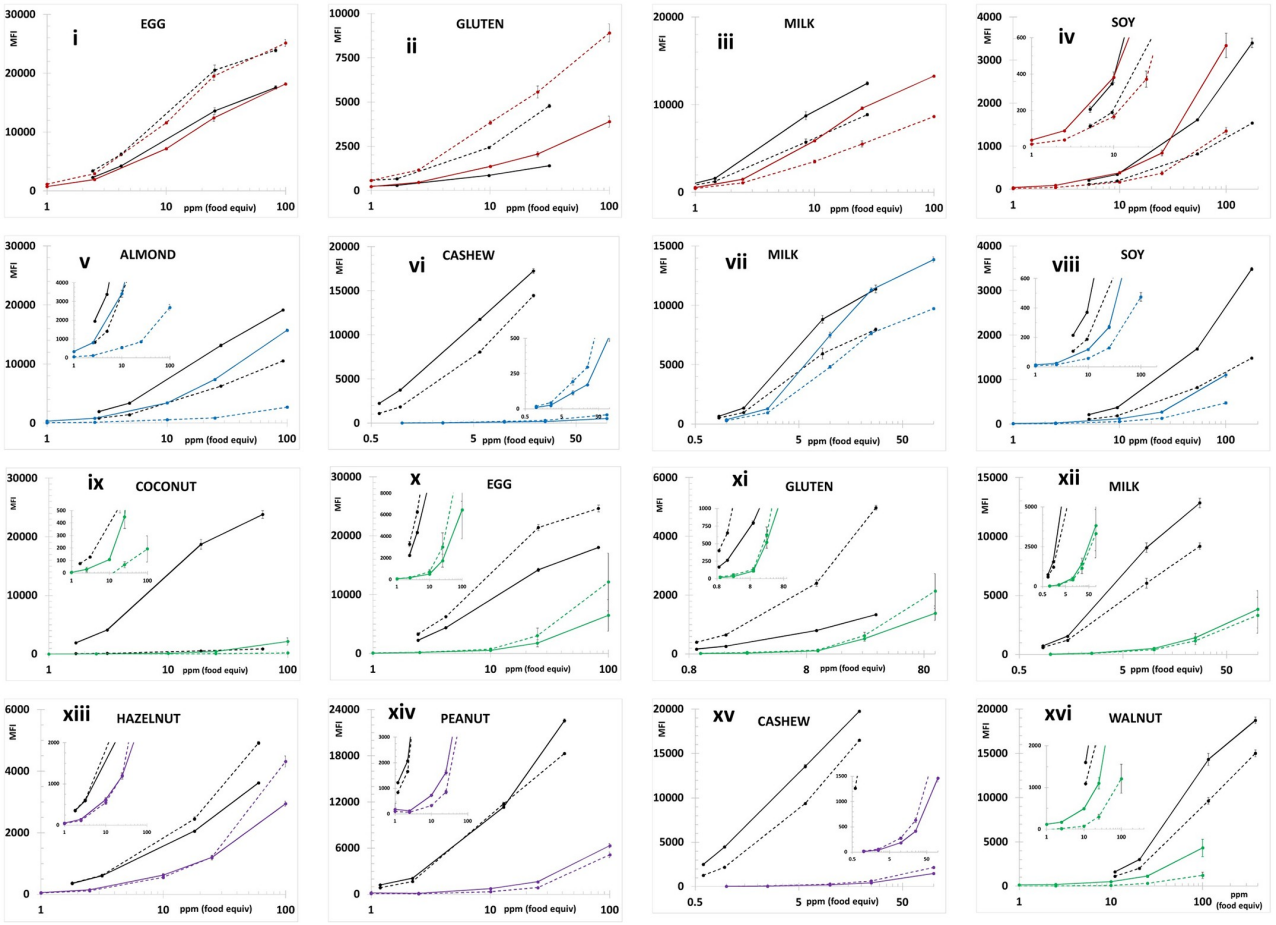
Average MFI of bead sets generating MFI with incurred food samples by Lab 01. Same as Fig 4 except the average of only the triplicate analyses generated by Lab 01.

**Fig 6:**
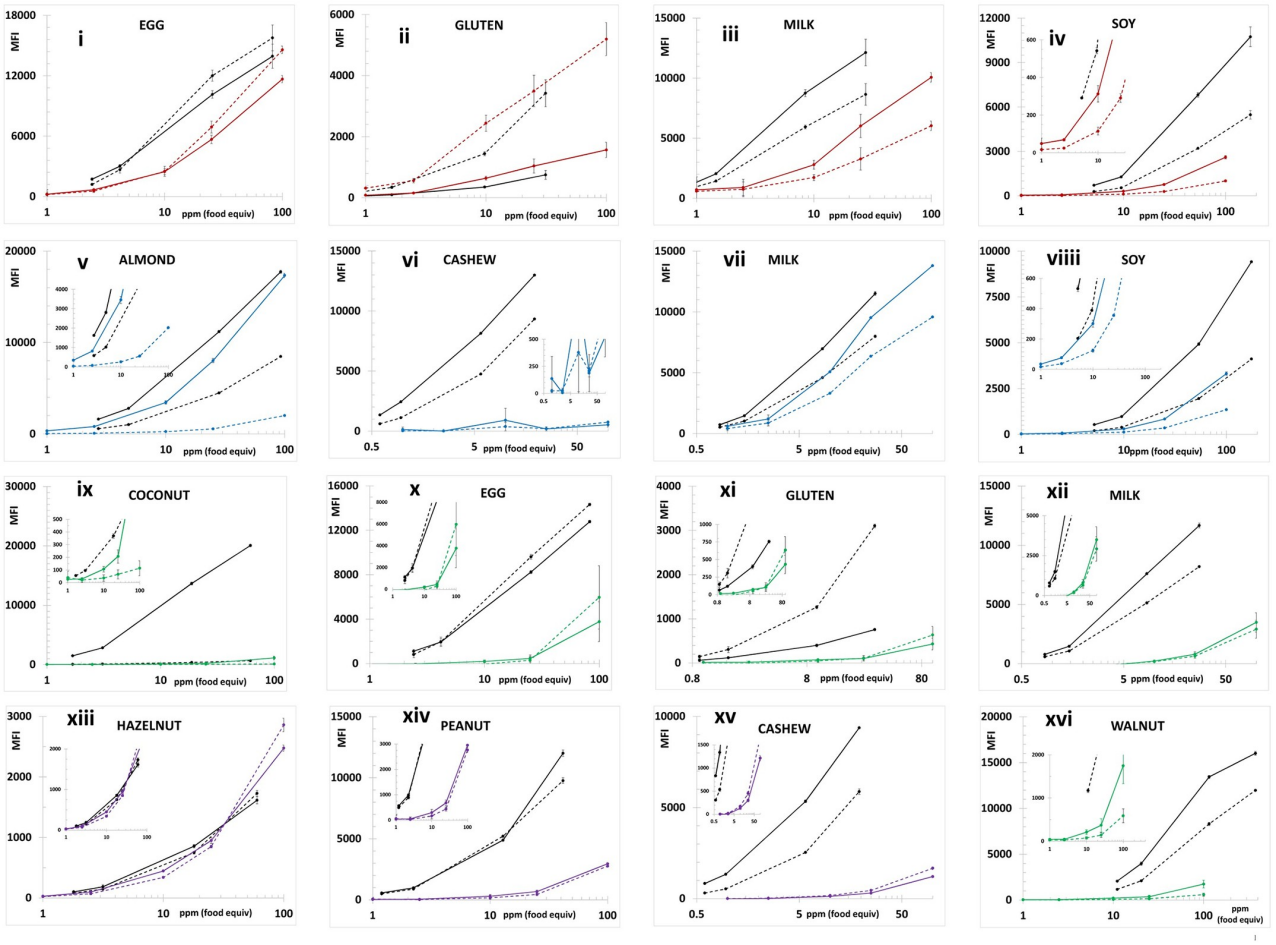
Average MFI of bead sets generating MFI with incurred food samples by Lab 03. Same as Fig 4 except the average of only the triplicate analyses generated by Lab 03.

**Fig 7:**
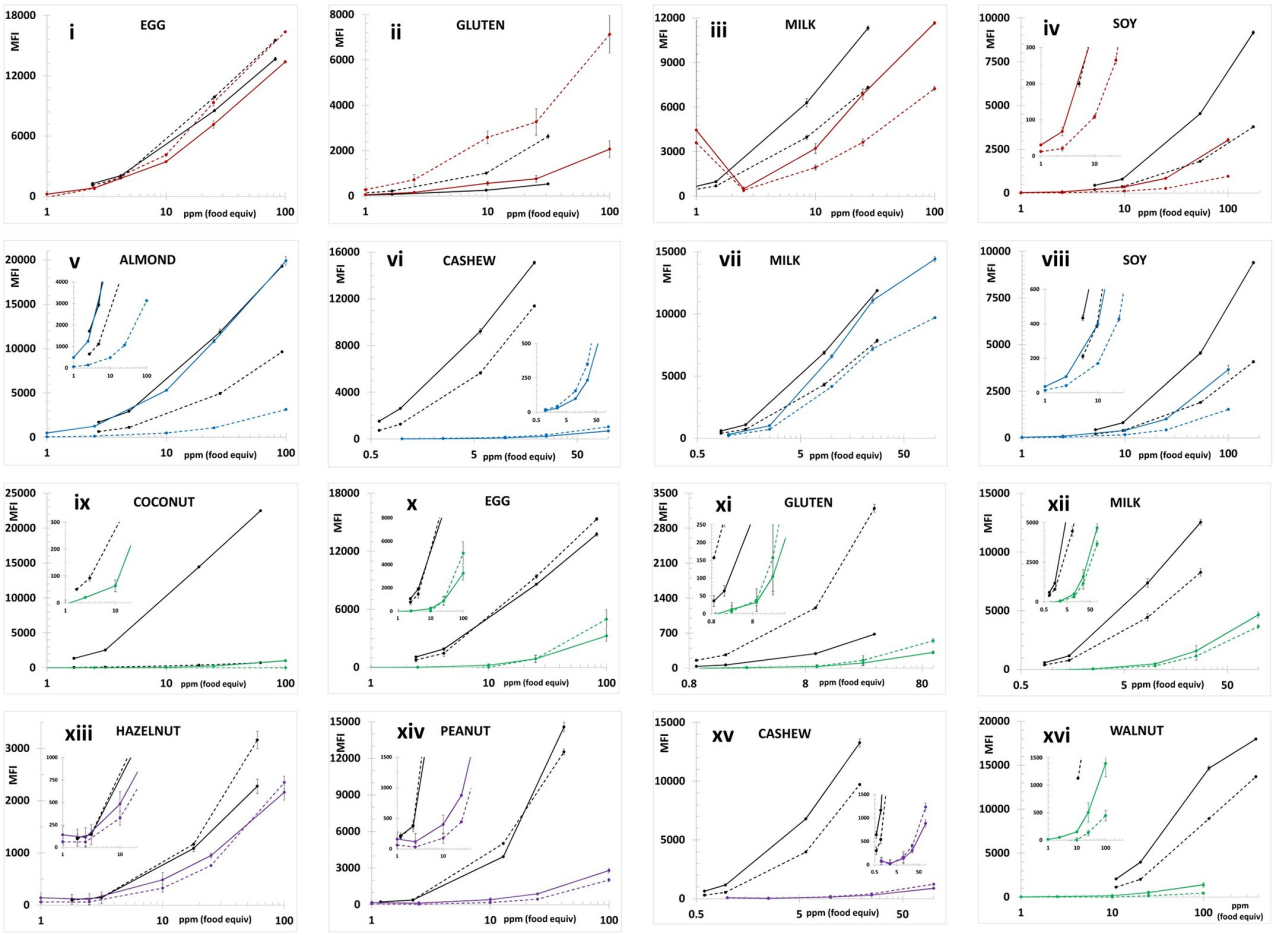
Average MFI of bead sets generating MFI with incurred food samples by Lab 11. Same as Fig 4 except the average of only the triplicate analyses generated by Lab 11.

The results of the MLV indicate that despite relatively high levels of variance between the absolute MFI measured by the participants in different laboratories, failures to correctly qualitatively detect the incurred analytes were rare and limited to the baked muffins. Specifically, two labs (05 and 10) generated poor response curves for gluten and the confirmatory responses with coconut-21 and walnut -48 were problematic for two labs each (10 & 11, 06 & 09, respectively). In all other cases the analytes were detected, though at times the intensities of the MFI were not ideal necessitating looking for positive increases with analyte concentration. Fig 4 depicts the overall average MFI responses across the 11 labs and the positive responses generated by three labs (Figs 5–7). The three labs (01, 03, and 11) were chosen as representatives of different levels of experience and proficiency. The three labs correctly detected the incurred analytes, with the only exception being the inability of Lab 11 to observe confirmatory responses with coconut -21. In addition, low positive responses for cashew (-18 and -19) in orange juice and dark chocolate were observed. Though the standard deviations based on MFI across all labs were large (Fig 4), as was observed with the calibration standards, the data generated within a lab displayed excellent reproducibility (Figs 5–7). The two weak data sets with the cashew bead sets (observed by many analysts) represents cross-reactivity as evidenced by the low intensity, bordering on background, and the ratio between the two bead sets. Specifically, in both instances, the relative magnitude of the MFI generated by the two complementary cashew bead sets changed from cashew-18 > cashew-19 observed with the calibration standards (black solid line > black dashed line) to cashew-18 ≤ cashew-19 for the orange juice and dark chocolate samples (colored solid line ≤ colored dashed line).

### Ratio analysis

A key feature of the xMAP FADA is the use of complementary antibody bead sets and requiring concurrence. Though this feature can be applied on a qualitative basis, a quantitative approach is superior and can be used to distinguish between cross-reactive homologues and the effects of different forms of processing. This quantitative approach is the basis of ratio analysis and multi-antibody profiling; profiling being the ratios between more than just the complementary antibody bead sets. In generating ratios and profiles, the more intense bead set is referred to as the anchor and becomes the denominator when calculating ratios and profiles. Focus on the higher avidity antibody reduces the likelihood of its undergoing saturation by assuring that its intensity is within the dynamic range of the assay. As such, any reduced precision is associated with the lower avidity antibody and the onset of its dynamic response. Thus, the possibility of underestimating allergen content due to the onset of saturation of the higher avidity antibody is reduced and instead the possibility of reduced precision (error) is shifted to the lower avidity antibody and it is not being in the dynamic range. As a result, the error is shifted to having less accuracy at low concentrations where the likelihood of a potential health risk is significantly reduced.

A key premise of ratio analysis is that a positive response that increases with analyte concentration indicates the presence of an antigenic analyte. The antigenic analyte may be the target analyte or a homologous, cross-reactive agent. It is unlikely that two different proteins would contain the same antigenic elements in identical environments (surrounding conformation of the protein) and thus the avidities would be different. As a result, the ratio between the MFI generated by complementary bead sets is unlikely to be the same for two different antigenic agents, provided the concentrations are not saturating. This approach provides a powerful analytical tool provided the target analyte and the reference material have undergone similar modifications (e.g., processing, covalent modification, conformational changes). Thus, ratio analysis provides a reliable secondary endpoint which is enhanced by also comparing the multi-antibody profiles generated with the other bead sets present in the cocktail. If the antigenic element was modified due to processing, it is important to compare the ratio and profile to appropriately, similarly processed standards. While this may complicate the use of such secondary endpoints, it is no different from what is observed with the commonly used ELISAs, with the advantage that it is less likely to generate false negative results due to the built-in redundancy. Further, as research characterizing the effects of processing on antigenicity increases [7], it should ultimately be possible to use these secondary endpoints to determine the type and extent of food processing. This is a potentially important since it is known that various forms of food processing affect allergenicity, and until a biological activity assay is developed, such may provide a useful tool.

Ratio analysis and multi-antibody profiling have been successfully applied to the detection of undeclared food allergens (e.g., raw peanut in garlic) [8], distinguishing between peanut, soy, and other legumes that cross-react with the peanut and soy antibodies at high concentrations [7], detection of allergenic foods (e.g., pecan) without having specific target antibodies [9], and for detecting and distinguishing between botanicals from the same plant families [10].

The calibration standards used in the xMAP FADA are derived from raw reference materials. As such, the ratio analysis data generated with the incurred, processed food samples may differ from the ratios generated by the calibration samples. This difference does not adversely affect the purpose of the MLV, which focuses on the examination of inter-laboratory ability to generate reliable data by analysts of varying proficiency. Indeed, the data gathered from the MLV may add to the database being developed regarding the effects of food processing on complementary bead set ratios and multi-antibody profiles.

Table 9 compares the effects of changing analyte concentration on the average, across all participating labs, of the complementary antibody pair MFI ratios. Of particular interest, are the standard deviations and %CV (RSD_R_) values describing inter-lab variance. As expected, the variances improved with increasing concentration of analyte with %CV (RSD_R_) values ≤47% for S1, S2, S5, and S7 of 18%, 14%, and 12% of the entries, respectively. Indeed, 29% of the complementary bead sets for S7 displayed %CV values of ≤5% and the percentage of ratios with %CV (RSD_R_) values in excess of 20% decreased from 57% for S1 to 7% for S7.

**Table 9:** Ratios between complementary antibody bead sets by calibration standards^a^.

	Complementary	ALMOND	BRAZIL NUT	CASSHEW	COCONUT	EGG ^c^	GLUTEN ^c^	HAZELNUT	MACADAMIA	MILK ^c^	PEANUT	PINE NUT	PISTACHIO	SOY	WALNUT
	Ratios ^b^	13/12	15/14	19/18	21/20	25/26	27/28	30/29	34/33	36/35	38/37	42/39	44/43	46/45	48/47
	**Ratio (brown second antibody/first)**														
**AVERAGE**	**S0**	0.59	$\begin{array}{c} 0.61 \end{array}$	$\begin{array}{c} 0.35 \end{array}$	$\begin{array}{c} 0.44 \end{array}$	**0.58**	**0.39**	$\begin{array}{c} 0.27 \end{array}$	$\begin{array}{c} 0.55 \end{array}$	$\begin{array}{c} 1.22 \end{array}$	$\begin{array}{c} 0.57 \end{array}$	$\begin{array}{c} 0.24 \end{array}$	$\begin{array}{c} 0.58 \end{array}$	$\begin{array}{c} 0.97 \end{array}$	$\begin{array}{c} 0.42 \end{array}$
	**S1**	$\begin{array}{c} 0.39 \end{array}$	$\begin{array}{c} 0.37 \end{array}$	$\begin{array}{c} 0.46 \end{array}$	$\begin{array}{c} 0.04 \end{array}$	0.59 ^d^	**0.37**	$\begin{array}{c} 0.83 \end{array}$	$\begin{array}{c} 0.06 \end{array}$	0.94	$\begin{array}{c} 0.77 \end{array}$	$\begin{array}{c} 0.10 \end{array}$	$\begin{array}{c} 0.08 \end{array}$	$\begin{array}{c} 0.46 \end{array}$	$\begin{array}{c} 0.60 \end{array}$
	**S2**	$\begin{array}{c} 0.38 \end{array}$	$\begin{array}{c} 0.38 \end{array}$	$\begin{array}{c} 0.45 \end{array}$	$\begin{array}{c} 0.04 \end{array}$	**1.12**	**0.38**	$\begin{array}{c} 0.85 \end{array}$	$\begin{array}{c} 0.06 \end{array}$	$\begin{array}{c} 0.74 \end{array}$	$\begin{array}{c} 0.79 \end{array}$	$\begin{array}{c} 0.12 \end{array}$	$\begin{array}{c} 0.06 \end{array}$	$\begin{array}{c} 0.48 \end{array}$	$\begin{array}{c} 0.60 \end{array}$
	**S5**	$\begin{array}{c} 0.43 \end{array}$	$\begin{array}{c} 0.34 \end{array}$	$\begin{array}{c} 0.56 \end{array}$	$\begin{array}{c} 0.03 \end{array}$	**0.82**	**0.33**	$\begin{array}{c} 0.98 \end{array}$	$\begin{array}{c} 0.14 \end{array}$	$\begin{array}{c} 0.66 \end{array}$	$\begin{array}{c} 1.1 \end{array}$	$\begin{array}{c} 0.21 \end{array}$	$\begin{array}{c} 0.08 \end{array}$	$\begin{array}{c} 0.44 \end{array}$	$\begin{array}{c} 0.64 \end{array}$
	**S7**	$\begin{array}{c} 0.51 \end{array}$	$\begin{array}{c} 0.33 \end{array}$	$\begin{array}{c} 0.70 \end{array}$	$\begin{array}{c} 0.03 \end{array}$	**0.84**	**0.28**	$\begin{array}{c} 1.15 \end{array}$	$\begin{array}{c} 0.25 \end{array}$	$\begin{array}{c} 0.69 \end{array}$	$\begin{array}{c} 0.86 \end{array}$	$\begin{array}{c} 0.28 \end{array}$	$\begin{array}{c} 0.13 \end{array}$	$\begin{array}{c} 0.43 \end{array}$	$\begin{array}{c} 0.76 \end{array}$
**STDEV**	**S0**	$\begin{array}{c} 0.15 \end{array}$	$\begin{array}{c} 0.08 \end{array}$	$\begin{array}{c} 0.08 \end{array}$	$\begin{array}{c} 0.06 \end{array}$	**0.17**	**0.10**	$\begin{array}{c} 0.06 \end{array}$	$\begin{array}{c} 0.08 \end{array}$	$\begin{array}{c} 0.47 \end{array}$	$\begin{array}{c} 0.20 \end{array}$	$\begin{array}{c} 0.08 \end{array}$	$\begin{array}{c} 0.14 \end{array}$	$\begin{array}{c} 0.21 \end{array}$	$\begin{array}{c} 0.16 \end{array}$
	**S1**	$\begin{array}{c} 0.03 \end{array}$	$\begin{array}{c} 0.10 \end{array}$	$\begin{array}{c} 0.05 \end{array}$	$\begin{array}{c} 0.01 \end{array}$	1.92	**0.10**	$\begin{array}{c} 0.14 \end{array}$	$\begin{array}{c} 0.01 \end{array}$	0.67	$\begin{array}{c} 0.31 \end{array}$	$\begin{array}{c} 0.02 \end{array}$	$\begin{array}{c} 0.04 \end{array}$	$\begin{array}{c} 0.04 \end{array}$	$\begin{array}{c} 0.08 \end{array}$
	**S2**	$\begin{array}{c} 0.03 \end{array}$	$\begin{array}{c} 0.06 \end{array}$	$\begin{array}{c} 0.05 \end{array}$	$\begin{array}{c} 0.01 \end{array}$	**0.33**	**0.10**	$\begin{array}{c} 0.12 \end{array}$	$\begin{array}{c} 0.01 \end{array}$	$\begin{array}{c} 0.07 \end{array}$	$\begin{array}{c} 0.11 \end{array}$	$\begin{array}{c} 0.02 \end{array}$	$\begin{array}{c} 0.02 \end{array}$	$\begin{array}{c} 0.04 \end{array}$	$\begin{array}{c} 0.09 \end{array}$
	**S5**	$\begin{array}{c} 0.02 \end{array}$	$\begin{array}{c} 0.06 \end{array}$	$\begin{array}{c} 0.06 \end{array}$	$\begin{array}{c} 0.00 \end{array}$	**0.10**	**0.07**	$\begin{array}{c} 0.15 \end{array}$	$\begin{array}{c} 0.02 \end{array}$	$\begin{array}{c} 0.06 \end{array}$	$\begin{array}{c} 0.12 \end{array}$	$\begin{array}{c} 0.03 \end{array}$	$\begin{array}{c} 0.02 \end{array}$	$\begin{array}{c} 0.04 \end{array}$	$\begin{array}{c} 0.04 \end{array}$
	**S7**	$\begin{array}{c} 0.03 \end{array}$	$\begin{array}{c} 0.05 \end{array}$	$\begin{array}{c} 0.06 \end{array}$	$\begin{array}{c} 0.00 \end{array}$	**0.08**	**0.05**	$\begin{array}{c} 0.18 \end{array}$	$\begin{array}{c} 0.04 \end{array}$	$\begin{array}{c} 0.03 \end{array}$	$\begin{array}{c} 0.07 \end{array}$	$\begin{array}{c} 0.04 \end{array}$	$\begin{array}{c} 0.04 \end{array}$	$\begin{array}{c} 0.02 \end{array}$	$\begin{array}{c} 0.02 \end{array}$
**%CV**	**S0**	$\begin{array}{c} 26 \end{array}$	$\begin{array}{c} 13 \end{array}$	$\begin{array}{c} 23 \end{array}$	$\begin{array}{c} 14 \end{array}$	**29**	**27**	$\begin{array}{c} 21 \end{array}$	$\begin{array}{c} 14 \end{array}$	$\begin{array}{c} 39 \end{array}$	$\begin{array}{c} 35 \end{array}$	$\begin{array}{c} 33 \end{array}$	$\begin{array}{c} 24 \end{array}$	$\begin{array}{c} 21 \end{array}$	$\begin{array}{c} 39 \end{array}$
	**S1**	$\begin{array}{c} 8 \end{array}$	$\begin{array}{c} 28 \end{array}$	$\begin{array}{c} 11 \end{array}$	$\begin{array}{c} 24 \end{array}$	323.	**27**	$\begin{array}{c} 17 \end{array}$	$\begin{array}{c} 24 \end{array}$	71	$\begin{array}{c} 40 \end{array}$	$\begin{array}{c} 17 \end{array}$	$\begin{array}{c} 52 \end{array}$	$\begin{array}{c} 8 \end{array}$	$\begin{array}{c} 14 \end{array}$
	**S2**	$\begin{array}{c} 8 \end{array}$	$\begin{array}{c} 16 \end{array}$	$\begin{array}{c} 10 \end{array}$	$\begin{array}{c} 23 \end{array}$	**29**	**26**	$\begin{array}{c} 14 \end{array}$	$\begin{array}{c} 18 \end{array}$	$\begin{array}{c} 10 \end{array}$	$\begin{array}{c} 14 \end{array}$	$\begin{array}{c} 17 \end{array}$	$\begin{array}{c} 36 \end{array}$	$\begin{array}{c} 9 \end{array}$	$\begin{array}{c} 15 \end{array}$
	**S5**	$\begin{array}{c} 5 \end{array}$	$\begin{array}{c} 18 \end{array}$	$\begin{array}{c} 12 \end{array}$	$\begin{array}{c} 16 \end{array}$	**13**	**21**	$\begin{array}{c} 16 \end{array}$	$\begin{array}{c} 16 \end{array}$	$\begin{array}{c} 9 \end{array}$	$\begin{array}{c} 11 \end{array}$	$\begin{array}{c} 14 \end{array}$	$\begin{array}{c} 30 \end{array}$	$\begin{array}{c} 8 \end{array}$	$\begin{array}{c} 7 \end{array}$
	**S7**	$\begin{array}{c} 5 \end{array}$	$\begin{array}{c} 15 \end{array}$	$\begin{array}{c} 9 \end{array}$	$\begin{array}{c} 7 \end{array}$	**9**	**20**	$\begin{array}{c} 16 \end{array}$	$\begin{array}{c} 16 \end{array}$	$\begin{array}{c} 4 \end{array}$	$\begin{array}{c} 9 \end{array}$	$\begin{array}{c} 17 \end{array}$	$\begin{array}{c} 34 \end{array}$	$\begin{array}{c} 5 \end{array}$	$\begin{array}{c} 3 \end{array}$

^*a*^ Ratios between MFI by complementary antibody bead sets as averaged for 4 experiments (3 for Lab 05) and then across the 11 participating laboratories.

^*b*^ Ratio is defined as the MFI by the bead set generating the smaller response divided by the MFI generated by the bead set generating the larger response for the reference material/calibration standards. Ratios in which the numerically first bead set is divided by the second are in black font (e.g., Gluten‐27/‐28), the reverse in brown (Almond‐13/‐12). No Ratio for Crustacean, only a single antibody ‐bead set (‐22).

^*c*^ Did not include Lab 10 gluten (‐27 & ‐28) data; Lab 07 Milk (‐35 & ‐36) data. Lab 09 did not collect egg (‐25 & ‐26) or milk (‐35 & ‐36) data. Lab 05 only performed 3 experiments.

^*d*^ Standard deviations, and %CV, for the average Ratios calculated for S1 of Egg and Milk high (red font); indicates unreliability in the Ratio for Egg and Milk at concentrations equivalent to S1.

Ratio analysis provides a stringent measure of analyte detection and inter-laboratory variance. Table 10 lists the ratios determined for the various analytes detected in the food samples. The ratios are only presented when the average responses (across all 11 labs) of the complementary antibody bead sets when both exceed the MFI of their respective S1 calibration standards, the lower limit of the calibration curves. The participants all correctly detected the presence of walnut in the baked muffins, though two displayed poor confirmatory results with walnut-48. However, the walnut data is not included in the table because the average (across all 11 labs) MFI for one of the walnut bead sets (-47 or -48) did not exceed S1 (equivalent to 11 ppm in the food sample, Table 1); within the dynamic range defined by the calibration standards. Using this stringent requirement, concurrence between complementary antibody bead sets within the dynamic range defined by S1 and S7, yielded highly reproducible limits of detection and quantification.

**Table 10:** Ratio analysis of complementary antibody pairs^a^, ^b^, ^c^, ^d^.

	ng/mL in analytical sample	ALMOND 13/12			COCONUT 21/20			EGG 25/26			GLUTEN 27/28			HAZELNUT 30/29			MILK 36/35			PEANUT 38/37			SOY 46/45			WALNUT 48/47		
		av		stdev	av		stdev	av		stdev	av		stdev	av		stdev	av		stdev	av		stdev	av		stdev	av		stdev
**CALIBRATION STANDARDS**																												
	**S1**	**0.39**	±	**0.03**	**0.038**	±	**0.009**	**0.59**	±	**1.92**	**0.37**	±	**0.10**	**0.83**	±	**0.14**	**0.94**	±	**0.67**	**0.77**	±	**0.31**	**0.46**	±	**0.04**	**0.60**	±	**0.08**
	**S2**	**0.38**	±	**0.03**	**0.036**	±	**0.009**	**1.12**	±	**0.33**	**0.38**	±	**0.10**	**0.85**	±	**0.12**	**0.74**	±	**0.07**	**0.79**	±	**0.11**	**0.48**	±	**0.04**	**0.60**	±	**0.09**
	**S5**	**0.43**	±	**0.02**	**0.027**	±	**0.004**	**0.82**	±	**0.10**	**0.33**	±	**0.07**	**0.98**	±	**0.15**	**0.66**	±	**0.06**	**1.10**	±	**0.12**	**0.44**	±	**0.04**	**0.64**	±	**0.04**
	**S7**	**0.51**	±	**0.03**	**0.031**	±	**0.002**	**0.84**	±	**0.08**	**0.28**	±	**0.05**	**1.15**	±	**0.18**	**0.69**	±	**0.03**	**0.86**	±	**0.07**	**0.43**	±	**0.02**	**0.76**	±	**0.02**
MEAT ^e^	**5**							<dyn ^g^			**$\begin{array}{r} 0.30 \end{array}$**	±	**0.10**										<dyn					
	**12.5**										**$\begin{array}{r} 0.34 \end{array}$**	±	**$\begin{array}{l} 0.08 \end{array}$**										<dyn					
	**50**							**$\begin{array}{c} 0.86 \end{array}$**	±	**$\begin{array}{l} 0.11 \end{array}$**	**$\begin{array}{r} 0.33 \end{array}$**	±	**$\begin{array}{l} 0.05 \end{array}$**				**$\begin{array}{r} 0.52 \end{array}$**	±	**$\begin{array}{l} 0.29 \end{array}$**									
	**125**							**$\begin{array}{c} 0.79 \end{array}$**	±	**$\begin{array}{l} 0.07 \end{array}$**	**$\begin{array}{r} 0.33 \end{array}$**	±	**$\begin{array}{l} 0.04 \end{array}$**				**$\begin{array}{r} 0.46 \end{array}$**	±	**$\begin{array}{l} 0.29 \end{array}$**				**$\begin{array}{r} 0.37 \end{array}$**	±	**$\begin{array}{l} 0.06 \end{array}$**			
	**500**							**$\begin{array}{c} 0.84 \end{array}$**	±	**$\begin{array}{l} 0.09 \end{array}$**	**$\begin{array}{r} 0.36 \end{array}$**	±	**$\begin{array}{l} 0.05 \end{array}$**				**$\begin{array}{r} 0.58 \end{array}$**	±	**$\begin{array}{l} 0.12 \end{array}$**				**$\begin{array}{r} 0.37 \end{array}$**	±	**$\begin{array}{l} 0.05 \end{array}$**			
^***ORANGEJUICE***^ ^e^																												
	**5**	**<dyn**																					<dyn					
	**12.5**	**<dyn**																					<dyn					
	**50**																**$\begin{array}{r} 0.65 \end{array}$**	±	**$\begin{array}{l} 0.12 \end{array}$**									
	**125**	**$\begin{array}{r} 0.10 \end{array}$**	±	**$\begin{array}{l} 0.03 \end{array}$**													**$\begin{array}{r} 0.67 \end{array}$**	±	**$\begin{array}{l} 0.08 \end{array}$**				**$\begin{array}{r} 0.49 \end{array}$**	±	**$\begin{array}{l} 0.09 \end{array}$**			
	**500**	**$\begin{array}{r} 0.15 \end{array}$**	±	**$\begin{array}{l} 0.04 \end{array}$**													**$\begin{array}{r} 0.67 \end{array}$**	±	**$\begin{array}{l} 0.07 \end{array}$**				**$\begin{array}{r} 0.44 \end{array}$**	±	**$\begin{array}{l} 0.05 \end{array}$**			
BAKEDMUFFIN ^e^																												
	**5**				**<dyn**																					**<dyn**		
	**12.5**																									**<dyn**		
	**50**																									**<dyn**		
	**125**							**$\begin{array}{r} 0.67 \end{array}$**	±	**$\begin{array}{l} 1.54 \end{array}$**	**$\begin{array}{r} 0.82 \end{array}$**	±	**$\begin{array}{l} 0.27 \end{array}$**				**$\begin{array}{r} 0.77 \end{array}$**	±	**$\begin{array}{l} 0.03 \end{array}$**							*h*		
	**500**				**$\begin{array}{r} 0.07 \end{array}$**	±	**$\begin{array}{l} 0.05 \end{array}$**	**$\begin{array}{r} 0.68 \end{array}$**	±	**$\begin{array}{l} 0.08 \end{array}$**	**$\begin{array}{r} 0.73 \end{array}$**	±	**$\begin{array}{l} 0.16 \end{array}$**				**$\begin{array}{r} 0.83 \end{array}$**	±	**$\begin{array}{l} 0.03 \end{array}$**									
DARKCHOCOLATE ^f^																												
	**5**													**<dyn**			**n.a**. ^i^			**<dyn**								
	**12.5**													**<dyn**														
	**50**													**$\begin{array}{c} 0.72 \end{array}$**	±	**$\begin{array}{l} 0.11 \end{array}$**												
	**125**													**$\begin{array}{c} 0.81 \end{array}$**	±	**$\begin{array}{l} 0.13 \end{array}$**												
	**500**													**$\begin{array}{c} 1.10 \end{array}$**	±	**$\begin{array}{l} 0.2 \end{array}$**				**$\begin{array}{c} 0.74 \end{array}$**	±	**$\begin{array}{l} 0.13 \end{array}$**						

^*a*^ Average (±stdev) Ratios between complementary antibody pairs calculated across the participating laboratories. The anchor bead sets (larger MFI response bead sets) were used in the denominator to calculate the ratios. The calibration standards entailed averaging each participant's results (MFI) across all four experiments.

^*b*^ All data for which the Overall Average MFI, across all partiipants, exceeded the response generated by S1 with the appropriate bead set (lower limit of the quantitative dynamic range) for both complementary antibodies were used to calculate ratios.

^*c*^ Due to import problems, Lab 05 did not receive meat samples. Lab 09 failed to program the monitoring of egg (25,26) and milk (35,36). Further, Lab 05 egg and Lab 07 milk data for muffins were dropped as outliers. Lastly, for the calibration standards, the gluten data generated by lab‐10 and the Milk data by Lab‐07 were omitted as outliers.

^*d*^ Brackets represent analyte incurred in the food.

^*e*^ Meat, Orange Juice, and Muffin samples contining 1, 2.5, 10, 25, or 100 ppm analyte were extracted using PBST Protocol which entailed a 20‐fold dilution with PBST. A secondary, optional 10‐fold dilution with PBST was applied, thus the results are representative of the analysis of 0.1, 0.25, 1, 2.5, and 10 ppm analyte if the optional 10‐fold dilution was omitted.

^*f*^ Dark Chocolate samples containing 1, 2.5, 10, 25, or 100 μg/g analyte extracted using UD Buffer Protocol which entailed a 1:40 dilution. A secondary, optional 5‐fold dilution with UD Buffer was applied, thus the results represent the analysis of 0.2, 0.5, 2, 5, and 20 μg/g analyte if the optional 5‐fold dilution omitted. Since, UD Buffer contains milk, milk analysis was not applicable (n.a.).

^*g*^ '< dyn' indicates that the concentration of the allergenic food in the analytical sample is insufficient to exceed. S1 and would thereby, even at 100% recovery, not met the criteria for inclusion.

^*h*^ Ten of the 11 labs detected walnut. However, the overall average MFI, across all labs, for both bead sets ‐47 and ‐48 did not exceed the average S1 MFI for each bead set.

^*i*^ 'n.a.' not applicable. The chocolate samples were analyzed using UD buffer (extraction and dilution) which contains NFDM and therfore the milk bead set responses are not listed.

The limits for the various analytes in the analytical samples of the diluted food extracts varied from 5 to 125 ng/mL for meat, 50 to 125 ng/mL for orange juice, 50 to 500 ng/mL for dark chocolate, and 125 to 500 ng/mL for baked muffins (except for walnut). If the optional 10-fold (5-fold for UD buffer) dilution step was omitted this means the limits are 20-times the concentration in the analytical sample; in all but one case ≤ 10 ppm in the original food samples.

Of interest is how the derived ratios of the incurred food samples compare to those of the calibration standards and the associated standard deviations. Not surprisingly, the ratios of the analytes mixed into the ground (uncooked) beef were comparable to the calibration standards. The ratios observed for milk and soy in orange juice were comparable to the calibration standards while almond showed a significant change from 0.4–0.5 to 0.1–0.15. The change in the almond ratio appears to be primarily due to a decrease in the MFI generated by almond bead set-13 (Figs 4–7). It is not clear whether this decrease in the almond-13 MFI was due to the acidic environment (e.g., deamidation), reducing conditions (e.g., ascorbic acid), or other factors that might affect the conformation of the target analyte or result in modifications of an antigenic epitope involved in almond-13 detection of the analyte. As expected, baking caused changes in the ratios for all analytes except for milk, for which the ratios were comparable. No major change was observed for either hazelnut or peanut in dark chocolate. Instead of looking at the standard deviations, a better representation of the variances is presented in Table 11.

**Table 11:** Percent coefficient of variation (%CV) of complementary antibody ratios^a^.

	ng/mL in analytical sample ^b^	Almond ^c^	Coconut	Egg	Gluten	Hazelnut	Milk	Peanut	Soy	Walnut	av
	**S1**	**8**	**24**	**323**	**27**	**17**	**71**	**40**	**8**	**14**	**59**
**CALIBRATION**	**S2**	**8**	**23**	**29**	**26**	**14**	**10**	**14**	**9**	**15**	**16**
**STANDARDS**	**S5**	**5**	**16**	**13**	**21**	**16**	**9**	**11**	**8**	**7**	**12**
	**S7**	**5**	**7**	**9**	**20**	**16**	**4**	**9**	**5**	**3**	**9**
MEAT ^d^											
	**5**				**33**						
	**12.5**				**24**						
	**50**			**13**	**16**		**55**				
	**125**			**9**	**13**		**63**		**15**		
	**500**			**11**	**14**		**21**		**15**		
ORANGEJUICE ^d^											
	**5**										
	**12.5**										
	**50**						**18**				
	**125**	**27**					**12**		**17**		
	**500**	**24**					**10**		**10**		
BAKEDMUFFIN ^d^											
	**5**										
	**12.5**										
	**50**									***f***	
	**125**			**230**	**32**		**4**				
	**500**		**66**	**12**	**21**		**4**				
DARKCHOCOLATE^e^											
	**5**										
	**12.5**										
	**50**					**15**	**na**				
	**125**					**16**					
	**500**					**18**		**17**			

^*a*^ %CV values derived from average MFI and associated standard deviations calculated across all 11 labs for the incurred food samples. Included are only those ratios derived from data in which the overall average MFI for both complementary bead sets exceeded the average MFI of S1 for each bead set. Brackets represent that which alergenic foods were incurred into the food samples.

^*b*^ Concentration of allergenic food in the analytical sample following extraction and subsequent dilution, a net 200‐fold dilution of the original food sample. Ommision of optional dilution of extract provides 10X (5X for UD Buffer extracts) greater sensitivity.

^*c*^ Ratios calcualted as the ratio of Almond ‐13/‐12; Coconut ‐21/‐20; Egg ‐25/‐26; Gluten ‐27/‐28; Hazelnut ‐30/‐29; Milk‐36/‐35; Peanut ‐38/‐37; Soy ‐46/‐45; Walnut ‐48/‐47.

^*d*^ Meat, orange juice, and baked muffin samples prepared containing each analyte at either 0, 1, 2.5, 10, 25, or 100 ppm. These samples were diluted 20‐fold with PBST during extraction and diluted an (optional) aditional 10‐fold prior to analyis.

^*e*^ Dark Chocolate samples prepared containing each analyte at either 0, 1, 2.5, 10, 25, or 100 ppm. These samples were diluted 40‐fold with UD Buffer during extraction and diluted an (optional) aditional 5‐fold prior to analyis.

^*f*^ Ten of the 11 labs detected walnut. However, the overall average MFI, across all labs, forf both bead sets ‐47 and ‐48 did not exceed the average S1 MFI for each bead set.

Tabulated in Table 11 are the %CV (RSD_R_) values for the data presented in Table 10. The average %CV for the meat samples progressively decreases from 33% to 15% with increasing concentration. Similar patterns were observed for the other food samples with the %CV values not significantly changing for hazelnut in chocolate (approx. 16%) or milk in baked muffins which was 4% for both the 125 ng/mL and 500 ng/mL samples. The relatively low variances observed in the ratios, compared to the variances observed in the MFI, reflects how the ratios are based on the binding avidities to the complementary antibody bead sets, an inherent property of the analytes. Further, as the concentration of analyte increases, any contribution by noise becomes a smaller proportion, thereby improving the ratio’s reflection of the binding constants and not inter-laboratory performance.

### Recovery

To gauge a true estimate of the effects of matrix on analyte recovery, the single lab validation measured the recoveries associated with analyte detection from samples prepared by incurring each of the 15 allergenic foods individually and as a mixture of all 15 into five matrices (four food samples and buffer) and extracted versus spiked into extracts prepared from the same five (analyte-free) matrices at comparable concentrations. This approach circumvented the need to rely on the calibration standards to calculate recovery and the variance that might be associated with any slight cross-reactivities that may be associated with using a mixture of the allergenic foods as calibration standards. Further, it distinguished between the effects of food extracts and the ability to mobilize the allergens from the food matrix. Thus, the goal of the recovery measurements of the MLV was not to ascertain the reliability of the xMAP FADA nor its performance when used exclusively by highly proficient analysts but instead the variance that might be observed by a range of analysts, of whom 9-out-of-11 were not proficient with the assay.

Tables 12–14 tabulate the ppm of the allergenic foods detected in the various food samples by each laboratory. Quantitation was performed by a step-wise linear approach using two calibration points bracketing the measured MFI (minus background) of a given sample. The dynamic range applied to calculating the ppm of allergenic food detected was defined as the background (S0) plus 10-times the standard deviation (S0+10D) as the lower limit and the S7 calibration standard for the upper limit. The use of S0+10D, and not S1, was based on xMAP FADA S/N-1 data (Tables 5 and 8) and published data [11] that indicated that the lower end of the dynamic range could be extended. In conducting extrapolation analyses, there are potential disadvantages regarding measurement accuracy and possible distortions in any observed inter-laboratory variances. Average MFI responses by a bead set that were < S0+10D or > S7, were noted in the table as ‘under’ or ‘over’, respectively. Since, the food samples were designed to assess performance across the dynamic range of the xMAP FADA, ‘over’, ‘>‘, responses were expected for food samples incurred with 100 ug/g almond, coconut, egg, or hazelnut if the recovery was 100% and similarly for gluten, milk, and peanut if the recovery exceeded 50% (see Table 1). When performing food sample analyses, it is expected that the analyst might further dilute (or omit the optional dilution step) to optimize quantification, if such is desired.

**Table 12:** Interpolated analyte recovered from incurred food samples: Meat / Sausage^a^.

μg / gram food ^b^	Lowest Level ^c^	Lab number	EGG ^d^			GLUTEN			MILK			SOY		
			‐25	‐26	av % rec ^e^	‐27	‐28	av % rec	‐35	‐36	av % rec	‐45	‐46	av % rec
**1**	**0.1**	**Lab01**	0.79	0.82	81	0.9	1.0	93	0.6	0.6	57	0.9	0.7	84
**1**	**0.1**	**Lab02**	under	under		1.7	2.2	192	0.3	0.4	37	0.3	under	
**1**	**0.1**	**Lab03**	0.33	under		1.4	1.5	146	0.58	0.64	61	0.35	under	
**1**	**0.1**	**Lab04**	under	under		3.2	3.9	354	0.32	under		0.55	under	
**1**	**0.1**	**Lab05** ^f^												
**1**	**0.1**	**Lab06**	under	under		4.2	4.5	433	4.6	2.3	347	2.9	2.0	241
**1**	**0.1**	**Lab07**	under	under		1.3	1.2	128	under	0.9		under	under	
**1**	**0.1**	**Lab08**	under	under		1.4	2.3	184	7.3	7.3	731	0.2	under	
**1**	**0.1**	**Lab09** ^f^				1.4	1.6	152				under	under	
**1**	**0.1**	**Lab10**	2.8	3.8	326	8.4	3.3	584	under	under		0.3	under	
**1**	**0.1**	**Lab11**	under	under		1.4	2.2	180	6.0	7.7	684	0.4	under	
		**av** ^g^	_**<**_ ^h^	**<**		**2.5**	**2.4**	**245**	**2.8**	**2.8**	**320**	**0.7**	**<**	
		**stdev** ^g^				**2.3**	**1.2**	**159**	**3.1**	**3.2**	**322**	**0.9**		
		**%CV** ^g^				**91**	**49**	**65**	**108**	**115**	**101**	**123**		
**2.5**	**0.25**	**Lab01**	2.0	2.1	82	4.1	4.0	160	1.4	1.3	54	2.2	1.8	80
**2.5**	**0.25**	**Lab02**	2.1	under		4.0	4.4	169	1.0	1.1	42	0.8	0.5	25
**2.5**	**0.25**	**Lab03**	0.9	under		3.5	3.2	134	0.7	0.8	31	0.5	0.4	19
**2.5**	**0.25**	**Lab04**	2.0	under		6.6	6.2	256	1.0	1.1	42	1.6	1.1	54
**2.5**	**0.25**	**Lab05**												
**2.5**	**0.25**	**Lab06**	under	under		11.3	11.0	448	OVER	21.9		0.9	0.6	30
**2.5**	**0.25**	**Lab07**	2.3	under		5.5	4.6	203	1.0	1.0	40	under	under	
**2.5**	**0.25**	**Lab08**	under	under		5.5	5.4	219	8.0	8.0	320	1.4	2.3	74
**2.5**	**0.25**	**Lab09**				4.5	4.7	184				under	under	
**2.5**	**0.25**	**Lab10**	4.1	7.6	233	1.6	under		under	under		0.9	under	
**2.5**	**0.25**	**Lab11**	under	under		5.5	6.7	245	0.8	0.9	34	0.8	under	
		**av**	**2.2**	**<**		**5.2**	**5.6**	**224**	**2.0**	**4.5**	**80**	**1.1**	**<**	
		**stdev**	**1.0**			**2.6**	**2.3**	**93**	**2.7**	**7.4**	**106**	**0.6**		
		**%CV**	**45**			**49**	**41**	**41**	**134**	**164**	**132**	**50**		
**10**	**1**	**Lab01**	11	12	115	30	23	261	5.7	5.0	53	11	8.2	95
**10**	**1**	**Lab02**	9	11	100	OVER	OVER		4.6	3.8	42	3	2.3	29
**10**	**1**	**Lab03**	3	4	37	25	21	230	2.2	1.9	21	2	2.0	21
**10**	**1**	**Lab04**	9	11	100	OVER	30		4.0	3.8	39	6	3.9	49
**10**	**1**	**Lab05**												
**10**	**1**	**Lab06**	12	12	117	OVER	OVER		11.8	6.9	93	6	4.0	49
**10**	**1**	**Lab07**	10	10	98	OVER	22		1.1	1.0	11	3	2.8	30
**10**	**1**	**Lab08**	7	8	74	OVER	30		24	24	241	3	2.0	22
**10**	**1**	**Lab09**				30	24	267				3	under	
**10**	**1**	**Lab10**	14	22	179	OVER	24		under	under		4	4.1	39
**10**	**1**	**Lab11**	9	10	95	OVER	31		4.4	4.1	43	4	2.8	35
		**av**	**9**	**11**	**101**	_**>**_ ^h^	**25**		**7.2**	**6.4**	**68**	**4.5**	**3.6**	**41**
		**stdev**	**3**	**5**	**38**		**4**		**7.4**	**7.5**	**74**	**2.5**	**1.9**	**23**
		**%CV**	**32**	**43**	**37**		**16**		**103**	**118**	**109**	**56**	**53**	**55**
**25**	**2.5**	**Lab01**	23	24	93	OVER	OVER		13	8.1	42	27	22	98
**25**	**2.5**	**Lab02**	21	24	90	OVER	OVER		8	6.9	31	9	6	31
**25**	**2.5**	**Lab03**	12	14	52	OVER	OVER		6	4.3	20	6	5	21
**25**	**2.5**	**Lab04**	21	25	91	OVER	OVER		8	6.7	29	13	9	46
**25**	**2.5**	**Lab05**												
**25**	**2.5**	**Lab06**	28	31	119	OVER	OVER		OVER	OVER		14	10	49
**25**	**2.5**	**Lab07**	20	21	83	OVER	OVER		under	under		6	5	22
**25**	**2.5**	**Lab08**	16	16	63	OVER	OVER		OVER	OVER		6	5	22
**25**	**2.5**	**Lab09**				OVER	OVER					9	8	34
**25**	**2.5**	**Lab10**	36	62	197	OVER	OVER		under	under		10	8	37
**25**	**2.5**	**Lab11**	21	24	90	OVER	OVER		11	7.7		10	7	34
		**av**	**22**	**27**	**98**	**>**	**>**		**na** ^h^	**na**		**11**	**9**	**39**
		**stdev**	**7**	**14**	**42**							**6**	**5**	**23**
		**%CV**	**32**	**53**	**43**							**56**	**60**	**57**
**100**	**10**	**Lab01**	OVER	OVER		OVER	OVER		OVER	26		171	144	158
**100**	**10**	**Lab02**	82	OVER		OVER	OVER		23	17	20	34	26	30
**100**	**10**	**Lab03**	48	65	56	OVER	OVER		16	9	13	20	17	19
**100**	**10**	**Lab04**	81	OVER		OVER	OVER		25	21	23	50	37	44
**100**	**10**	**Lab05**												
**100**	**10**	**Lab06**	OVER	OVER		OVER	OVER		OVER	OVER		65	48	56
**100**	**10**	**Lab07**	OVER	OVER		OVER	OVER		6	1	3	29	24	26
**100**	**10**	**Lab08**	56	66	61	OVER	OVER		OVER	OVER		23	20	22
**100**	**10**	**Lab09**				OVER	OVER					37	34	35
**100**	**10**	**Lab10**	OVER	OVER		OVER	OVER		16	6	11	41	35	38
**100**	**10**	**Lab11**	79	OVER		OVER	OVER		OVER	27		36	28	32
		**av**	**>**	**>**		**>**	**>**		**17**	**15**	**14**	**51**	**41**	**46**
		**stdev**							**8**	**10**	**8**	**44**	**37**	**41**
		**%CV**							**45**	**67**	**56**	**88**	**90**	**89**

^*a*^ Interpolated ppm using the calibration standards (S1, S2, S5, S7) in a stepwise, linear analysis of the measured MFI (after subtracting background). S7 defines the upper limit of the dynamic range and 10‐times the standard deviation (10D) of the background (S0) defines the lower limit of quantitation. ppm calculated by converting the interpolated ng extractable protein/mL derived from the calibration standards, to ppm whole allergenic food using the protein content as referenced in Table 1. 'under' and 'OVER' indicate that the measured MFI was either less than or greater than the defined limits of the dynamic range (10D and S7).

^*b*^ μg incurred per gram of food to generate the test portion.

^*c*^ 'Lowest Level' refers to the lowest concentration of analyte that would generate the same analytical sample; by eliminating the optional dilution following extraction.

^*d*^ Incurred analyte and associated bead sets.

^*e*^ 'av % rec' is the average percent recovery by both complementary bead sets; if either is ''under' or 'OVER', no average is reported.

^*f*^ Lab05 did not analyze meat samples due to shipping / import problems. Lab09 failed to monitor bed sets ‐25, ‐26, ‐35, and ‐36 for egg and milk.

^*g*^ 'under' or 'OVER' entries are ignored when calculating averages, standard deviations, and %CV values. No entry indicates data insufficient to support calculation.

^*h*^ '<', '>' indicate MFI responses by more than 3 labs either too low ('under') or too high ('over'). 'na' indicates more than 3 labs generating conflicting, out of range data with at least 2 'under' and 2 'over'.

**Table 13:** Interpolated analyte recovered from incurred food samples: Orange juice and dark chocolate^a^.

**μg / gram food** ^b^	**Lowest Level** ^c^	**Lab number**	**ALMOND** ^d^				**MILK**			**SOY**		
			**-12**	**-13**	**av % rec** ^e^	**-12%** ^*e*^	**-35**	**-36**	**av % rec**	**-45**	**-46**	**av % rec**
**1**	**0.1**	**Lab01**	0.45	0.17	31	45	0.48	0.47	47	under	under	
**1**	**0.1**	**Lab02**	0.79	0.27	53	79	0.56	0.52	54	0.48	0.54	51
**1**	**0.1**	**Lab03**	0.58	under		58	0.70	under		0.34	under	
**1**	**0.1**	**Lab04**	0.66	0.22	44	66	0.05	0.05	5	under	under	
**1**	**0.1**	**Lab05** ^f^	under	under			0.89	0.85	87	0.64	under	
**1**	**0.1**	**Lab06**	0.5	under		46	8.7	16	1224	under	under	
**1**	**0.1**	**Lab07**	0.7	0.3	46	65	0.9	1.1	101	under	under	
**1**	**0.1**	**Lab08**	0.9	0.3	58	86	under	20.2		under	under	
**1**	**0.1**	**Lab09** ^f^	0.8	under		75				under	under	
**1**	**0.1**	**Lab10**	0.7	under		69	under	under		under	under	
**1**	**0.1**	**Lab11**	0.8	0.3	51	78	0.4	0.5	46	under	under	
		**av** ^g^	**0.7**	_**<**_ ^h^		**67**	**1.6**	**4.9**	**223**	**<**	**<**	
		**stdev** ^g^	**0.1**			**14**	**2.9**	**8.1**	**442**			
		**%CV** ^g^	**20**			**20**	**181**	**166**	**198**			
**2.5**	**0.25**	**Lab01**	1.1	0.4	30	44	1.4	1.4	57	0.6	under	
**2.5**	**0.25**	**Lab02**	1.9	0.6	51	76	1.6	1.6	63	1.1	1.1	43
**2.5**	**0.25**	**Lab03**	1.4	under		55	1.2	1.3	50	0.7	0.9	33
**2.5**	**0.25**	**Lab04**	1.7	0.5	43	67	0.1	under		1.0	under	
**2.5**	**0.25**	**Lab05**	under	under			5.7	4.6	205	1.4	under	
**2.5**	**0.25**	**Lab06**	1.2	under		49	15	18	653	under	under	
**2.5**	**0.25**	**Lab07**	1.3	0.4	35	54	0.9	1.0	38	under	under	
**2.5**	**0.25**	**Lab08**	1.8	0.6	48	73	OVER	OVER		under	under	
**2.5**	**0.25**	**Lab09**	1.8	0.6	49	73				under	under	
**2.5**	**0.25**	**Lab10**	1.7	0.5	44	70	0.8	0.9	34	0.9	under	
**2.5**	**0.25**	**Lab11**	2.0	0.6	51	79	1.4	1.5	57	1.1	under	
		**av**	**1.6**	**0.5**	**44**	**64**	**3.1**	**3.8**	**145**	**<**	**<**	
		**stdev**	**0.3**	**0.1**	**8**	**12**	**4.6**	**5.9**	**213**			
		**%CV**	**19**	**18**	**17**	**19**	**149**	**156**	**147**			
**10**	**1**	**Lab01**	4.9	1.8	34	49	7.2	6.9	70	2.8	2.7	28
**10**	**1**	**Lab02**	9.8	2.3	61	98	8.2	8.1	82	4.8	4.7	48
**10**	**1**	**Lab03**	6.7	1.2	39	67	6.0	5.9	60	2.9	3.1	30
**10**	**1**	**Lab04**	9.2	1.7	55	92	1.6	1.4	15	4.6	4.2	44
**10**	**1**	**Lab05**	5.3	under		53	12	12	122	5.7	8.3	70
**10**	**1**	**Lab06**	5.3	0.7	30	53	OVER	OVER		2.3	under	
**10**	**1**	**Lab07**	18	4.1	109	176	25	27	263	25	23	240
**10**	**1**	**Lab08**	8.7	2.3	55	87	OVER	OVER		4.7	5.9	53
**10**	**1**	**Lab09**	9.1	2.4	57	91				4.1	5.1	46
**10**	**1**	**Lab10**	8.4	1.9	51	84	7.2	6.7	70	4.4	4.8	46
**10**	**1**	**Lab11**	11.0	2.0	65	110	8.1	8.1	81	4.7	4.2	45
		**av**	**8.7**	**2.1**	**56**	**87**	**9.5**	**9.6**	**95**	**6.0**	**6.6**	**65**
		**stdev**	**3.6**	**0.9**	**22**	**36**	**7.0**	**7.8**	**74**	**6.4**	**5.9**	**63**
		**%CV**	**41**	**44**	**40**	**41**	**74**	**81**	**78**	**107**	**89**	**96**
**25**	**2.5**	**Lab01**	14	2.8	34	57	27	25	104	6.7	6.4	26
**25**	**2.5**	**Lab02**	23	4.9	56	92	OVER	OVER		12	12	47
**25**	**2.5**	**Lab03**	19	2.6	44	77	19	18	75	8.2	8.7	34
**25**	**2.5**	**Lab04**	22	4.4	53	89	15	11	53	12	11	45
**25**	**2.5**	**Lab05**	20	under		81	OVER	27		14	11	50
**25**	**2.5**	**Lab06**	18	1.5	39	73	OVER	OVER		6.6	7.7	29
**25**	**2.5**	**Lab07**	21	4.1	51	85	25	25	98	9.2	8.3	35
**25**	**2.5**	**Lab08**	22	4.7	52	86	OVER	OVER		11.1	10.0	42
**25**	**2.5**	**Lab09**	25	5.8	62	101				9.8	11	42
**25**	**2.5**	**Lab10**	21	4.3	51	85	20	19	77	11	11	43
**25**	**2.5**	**Lab11**	25	4.6	60	102	25	24	97	11.8	10.7	45
		**av**	**21**	**4.0**	**50**	**84**	_**>**_ ^h^	**21**		**10**	**9.8**	**40**
		**stdev**	**3.1**	**1.3**	**9**	**13**		**5.5**		**2.3**	**1.7**	**8**
		**%CV**	**15**	**32**	**17**	**15**		**26**		**23**	**18**	**19**
**100**	**10**	**Lab01**	56	11	33	56	OVER	OVER	34		30	32
**100**	**10**	**Lab02**	OVER	18			OVER	OVER	44		45	44
**100**	**10**	**Lab03**	89	12	50	89	OVER	OVER	36		37	36
**100**	**10**	**Lab04**	90	19	54	90	OVER	OVER	44		41	42
**100**	**10**	**Lab05**	91	12	51	91	OVER	OVER	45		36	41
**100**	**10**	**Lab06**	80	5	42	80	OVER	OVER	33		29	31
**100**	**10**	**Lab07**	87	16	52	87	OVER	OVER	50		45	47
**100**	**10**	**Lab08**	88	18	53	88	OVER	OVER	40		40	40
**100**	**10**	**Lab09**	OVER	23					41		43	42
**100**	**10**	**Lab10**	OVER	17			OVER	OVER	38		41	40
**100**	**10**	**Lab11**	OVER	17			OVER	OVER	43		43	43
		**av**	**>**	**15**		**83**	**>**	**>**	**41**		**39**	**40**
		**stdev**		**4.9**		**13**			**5.1**		**5.7**	**5**
		**%CV**		**32**		**15**			**13**		**15**	**13**
**μg / gram food** ^b^	**Lowest Level** ^c^	**Lab number**	**μg / gram food** ^b^	**Lowest Level** ^c^	**Lab number**	**HAZELNUT** ^d^			**PEANUT**			
						**-29**	**-30**	**av % rec** ^e^	**-37**	**-38**	**av % rec**	
**1**	**0.1**	**Lab01**	**1**	**0.2**	**Lab01**	under	0.2		0.17	0.14	15	
**1**	**0.1**	**Lab02**	**1**	**0.2**	**Lab02**	under	0.4		0.1	0.0	5	
**1**	**0.1**	**Lab03**	**1**	**0.2**	**Lab03**	under	under		under	under		
**1**	**0.1**	**Lab04**	**1**	**0.2**	**Lab04**	0.50	0.47	49	under	under		
**1**	**0.1**	**Lab05** ^f^	**1**	**0.2**	**Lab05**	2.2	2.0	210	1.2	under		
**1**	**0.1**	**Lab06**	**1**	**0.2**	**Lab06**	under	under		under	under		
**1**	**0.1**	**Lab07**	**1**	**0.2**	**Lab07**	0.5	0.3	40	under	under		
**1**	**0.1**	**Lab08**	**1**	**0.2**	**Lab08**	under	under		under	under		
**1**	**0.1**	**Lab09** ^f^	**1**	**0.2**	**Lab09**	under	under		under	under		
**1**	**0.1**	**Lab10**	**1**	**0.2**	**Lab10**	0.9	0.6	79	0.1	under		
**1**	**0.1**	**Lab11**	**1**	**0.2**	**Lab11**	3.0	under		0.9	under		
		**av** ^g^			**av** ^g^	_**<**_ ^h^	**<**		**<**	**<**		
		**stdev** ^g^			**stdev** ^g^							
		**%CV** ^g^			**%CV** ^g^							
**2.5**	**0.25**	**Lab01**	**2.5**	**0.5**	**Lab01**	0.7	0.6	25	0.1	0.1	4	
**2.5**	**0.25**	**Lab02**	**2.5**	**0.5**	**Lab02**	1.3	1.0	47	0.2	0.1	6	
**2.5**	**0.25**	**Lab03**	**2.5**	**0.5**	**Lab03**	under	under		under	under		
**2.5**	**0.25**	**Lab04**	**2.5**	**0.5**	**Lab04**	1.6	1.4	61	0.2	under		
**2.5**	**0.25**	**Lab05**	**2.5**	**0.5**	**Lab04**	2.8	2.7	109	1.2	under		
**2.5**	**0.25**	**Lab06**	**2.5**	**0.5**	**Lab06**	under	0.9		under	under		
**2.5**	**0.25**	**Lab07**	**2.5**	**0.5**	**Lab07**	1.0	0.6	32	under	under		
**2.5**	**0.25**	**Lab08**	**2.5**	**0.5**	**Lab08**	under	under		under	under		
**2.5**	**0.25**	**Lab09**	**2.5**	**0.5**	**Lab09**	under	under		under	under		
**2.5**	**0.25**	**Lab10**	**2.5**	**0.5**	**Lab10**	2.5	1.8	84	0.3	under		
**2.5**	**0.25**	**Lab11**	**2.5**	**0.5**	**Lab11**	1.9	under		under	under		
		**av**			**av**	**<**	**<**		**<**	**<**		
		**stdev**			**stdev**							
		**%CV**			**%CV**							
**10**	**1**	**Lab01**	**10**	**2**	**Lab01**	3.4	2.8	31	0.7	0.5	6	
**10**	**1**	**Lab02**	**10**	**2**	**Lab02**	6.0	4.6	53	0.8	0.4	6	
**10**	**1**	**Lab03**	**10**	**2**	**Lab03**	8.9	7.9	84	0.6	under		
**10**	**1**	**Lab04**	**10**	**2**	**Lab04**	5.8	4.8	53	0.7	0.5	6	
**10**	**1**	**Lab05**	**10**	**2**	**Lab04**	5.7	5.8	58	1.3	under		
**10**	**1**	**Lab06**	**10**	**2**	**Lab06**	6.6	4.9	58	1.1	0.5	8	
**10**	**1**	**Lab07**	**10**	**2**	**Lab07**	4.2	2.5	34	0.5	0.3	4	
**10**	**1**	**Lab08**	**10**	**2**	**Lab08**	5.3	2.8	40	under	under		
**10**	**1**	**Lab09**	**10**	**2**	**Lab09**	1.7	1.3	15	under	under		
**10**	**1**	**Lab10**	**10**	**2**	**Lab10**	8.2	5.6	69	1.4	1.0	12	
**10**	**1**	**Lab11**	**10**	**2**	**Lab11**	8.5	5.7	71	2.3	under		
		**av**			**av**	**5.8**	**4.4**	**51**	**1.0**	**<**		
		**stdev**			**stdev**	**2.2**	**1.9**	**20**	**0.6**			
		**%CV**			**%CV**	**38**	**43**	**39**	**55**			
**25**	**2.5**	**Lab01**	**25**	**5**	**Lab01**	9.3	7.9	34	1.7	1.2	6	
**25**	**2.5**	**Lab02**	**25**	**5**	**Lab02**	14	12	53	2.0	1.0	6	
**25**	**2.5**	**Lab03**	**25**	**5**	**Lab03**	23	22	91	1.5	1.1	5	
**25**	**2.5**	**Lab04**	**25**	**5**	**Lab04**	18	16	67	2.2	1.5	7	
**25**	**2.5**	**Lab05**	**25**	**5**	**Lab04**	17	18	71	1.6	1.3	6	
**25**	**2.5**	**Lab06**	**25**	**5**	**Lab06**	16	13	57	1.9	1.2	6	
**25**	**2.5**	**Lab07**	**25**	**5**	**Lab07**	11	7.0	36	1.2	0.8	4	
**25**	**2.5**	**Lab08**	**25**	**5**	**Lab08**	12	7.9	40	5.9	5.2	22	
**25**	**2.5**	**Lab09**	**25**	**5**	**Lab09**	4.0	3.0	14	under	under		
**25**	**2.5**	**Lab10**	**25**	**5**	**Lab10**	17	13	61	2.9	2.1	10	
**25**	**2.5**	**Lab11**	**25**	**5**	**Lab11**	16	12	56	3.7	2.4	12	
		**av**			**av**	**14**	**12**	**53**	**2.5**	**1.8**	**8**	
		**stdev**			**stdev**	**5.1**	**5.5**	**21**	**1.4**	**1.3**	**5**	
		**%CV**			**%CV**	**35**	**46**	**40**	**57**	**72**	**63**	
**100**	**10**	**Lab01**	**100**	**20**	**Lab01**	42	50	46	7.1	5.9	7	
**100**	**10**	**Lab02**	**100**	**20**	**Lab02**	OVER	OVER		6.1	4.1	5	
**100**	**10**	**Lab03**	**100**	**20**	**Lab03**	OVER	OVER		7.6	6.8	7	
**100**	**10**	**Lab04**	**100**	**20**	**Lab04**	OVER	OVER		8.7	5.9	7	
**100**	**10**	**Lab05**	**100**	**20**	**Lab04**	OVER	OVER		4.8	2.0	3	
**100**	**10**	**Lab06**	**100**	**20**	**Lab06**	OVER	OVER		7.0	4.5	6	
**100**	**10**	**Lab07**	**100**	**20**	**Lab07**	54	38	46	5.6	4.0	5	
**100**	**10**	**Lab08**	**100**	**20**	**Lab08**	48	32	40	5.7	3.8	5	
**100**	**10**	**Lab09**	**100**	**20**	**Lab09**	16	15	15	2.1	2.0	2	
**100**	**10**	**Lab10**	**100**	**20**	**Lab10**	OVER	55		9.3	5.9	8	
**100**	**10**	**Lab11**	**100**	**20**	**Lab11**	56	43	49	9.5	6.0	8	
		**av**			**av**	_**>**_ ^h^	**>**		**6.7**	**4.6**	**6**	
		**stdev**			**stdev**				**2.2**	**1.6**	**2**	
		**%CV**			**%CV**				**32**	**35**	**33**	

^*a*^ Interpolated ppm using the calibration standards (S1, S2, S5, S7) in a stepwise, linear analysis of the measured MFI (after subtracting background). S7 defines the upper limit of the dynamic range and 10‐times the standard deviation (10D) of the background (S0) defines the lower limit of quantitation. ppm calculated by converting the interpolated ng extractable protein/mL derived from the calibration standards, to ppm whole allergenic food using the protein content as referenced in Table 1. 'under' and 'OVER' indicate that the measured MFI was either less than or greater than the defined limits of the dynamic range (10D and S7).

^*b*^ μg incurred per gram of food to generate the test portion.

^*c*^ 'Lowest Level' refers to the lowest concentration of analyte that would generate the same analytical sample; by eliminating the optional dilution following extraction.

^*d*^ Incurred analyte and associated bead sets.

^*e*^ *'*av % rec' is the average percent recovery by both complementary bead sets; if either is 'under' or 'OVER', no average is reported. '‐12%' is the percent recovery calcualted based on Almond‐12; only for Orange Juice samples.

^*f*^ Lab05 did not analyze meat samples due to shipping / import problems. Lab09 failed to monitor bed sets ‐25, ‐26, ‐35, and ‐36 for egg and milk.

^*g*^ 'Over' or 'under' entries are ignored when calculating averages, standard deviations, and %CV values. No entry indicates data insufficient to support calculation

^*h*^ '<', '>' indicate MFI responses by more than 3 labs either too low ('under') or too high ('over'). 'na' indicates more than 3 labs generating conflicting, out of range data with at leat 2 'under' and 2'over'.

**Table 14:** Interpolated analyte recovered from incurred food samples: Baked muffins^a^.

μg / gram food ^b^	Lowest Level ^c^	Lab number	Coconut ^d^			EGG			GLUTEN			MILK			WALNUT		
			‐20	‐21	av % rec ^e^	‐25	‐26	av % rec	‐27	‐28	av % rec	‐35	‐36	av % rec	‐47	‐48	av % rec
**1**	**0.1**	**Lab01**	under	under		0.1	under		under	under		0.0	under		0.8	under	
**1**	**0.1**	**Lab02**	under	under		under	under		under	under		under	under		under	under	
**1**	**0.1**	**Lab03**	under	1.2		under	under		under	under		under	under		under	0.26	
**1**	**0.1**	**Lab04**	under	under		under	under		under	under		under	under		under	under	
**1**	**0.1**	**Lab05** ^f^	under	under		under	under		under	2.4		under	under		under	under	
**1**	**0.1**	**Lab06**	under	under		under	under		under	under		0.7	0.7	69	under	under	
**1**	**0.1**	**Lab07**	0.1	11.7	592	1.4	3.5	247	3.7	3.3	354	OVER	OVER		1.6	2.1	186
**1**	**0.1**	**Lab08**	under	under		under	under		under	under		under	under		under	under	
**1**	**0.1**	**Lab09** ^f^	under	under					under	under					under	under	
**1**	**0.1**	**Lab10**	under	under		under	under		under	under		under	under		under	under	
**1**	**0.1**	**Lab11**	under	under		under	under		under	under		under	under		under	under	
		**av** ^g^	_**<**_ ^h^	**<**		**<**	**<**		**<**	**<**		**<**	**<**		**<**	**<**	
		**stdev** ^g^															
		**%CV** ^g^															
**2.5**	**0.25**	**Lab01**	0.0	under		0.2	under		0.2	under		0.1	0.1	5	1.2	under	
**2.5**	**0.25**	**Lab02**	under	under		under	under		under	under		0.1	0.1	4	0.2	under	
**2.5**	**0.25**	**Lab03**	0.0	under		under	under		under	under		under	under		under	0.3	
**2.5**	**0.25**	**Lab04**	0.0	under		under	under		1.0	1.2	45	under	under		under	under	
**2.5**	**0.25**	**Lab05**	under	under		under	under		5.0	12.2	344	4.5	6.1	213	under	under	
**2.5**	**0.25**	**Lab06**	under	under		under	under		0.3	under		0.0	under		under	under	
**2.5**	**0.25**	**Lab07**	0.2	under		1.4	2.6	78	1.6	under		OVER	OVER		1.8	under	
**2.5**	**0.25**	**Lab08**	under	under		under	under		under	under		3.6	3.6	145	under	0.5	
**2.5**	**0.25**	**Lab09**	under	under					under	under					under	under	
**2.5**	**0.25**	**Lab10**	under	under		under	under		2.5	under		0.7	0.7		under	under	
**2.5**	**0.25**	**Lab11**	under	under		under	under		under	under		0.1	under		under	under	
		**av**	**<**	**<**		**<**	**<**		**<**	**<**		**1.3**	**<**		**<**	**<**	
		**stdev**										**1.9**					
		**%CV**										**145**					
**10**	**1**	**Lab01**	0.1	under		0.6	0.5	6	0.6	0.3	5	0.6	0.6	6	3.4	0.7	21
**10**	**1**	**Lab02**	0.1	under		0.8	0.4	6	under	0.2		0.6	0.6	6	1.1	0.5	8
**10**	**1**	**Lab03**	0.1	1.1	6	0.4	under		1.1	under		0.2	0.3	2	1.1	0.7	9
**10**	**1**	**Lab04**	0.1	under		under	under		under	under		0.3	0.3	3	1.0	0.7	9
**10**	**1**	**Lab05**	under	under		under	under		under	5.4		under	under		under	under	
**10**	**1**	**Lab06**	0.2	1.1	6	under	under		2.4	1.0	17	0.2	0.3	2	under	under	
**10**	**1**	**Lab07**	0.6	15	77	3.3	3.5	34	7.1	1.3	42	OVER	OVER		7.6	6.4	70
**10**	**1**	**Lab08**	0.2	3.1	16	under	under		under	under		6.1	6.3	62	1.0	1.0	10
**10**	**1**	**Lab09**	under	under					under	under					under	under	
**10**	**1**	**Lab10**	0.1	under		under	under		1.6	under		0.5	0.4	5	under	under	
**10**	**1**	**Lab11**	0.1	under		under	under		under	under		0.7	0.7	7	under	under	
		**av**	**0.2**	**<**		**<**	**<**		**<**	**<**		**1.2**	**1.2**	**12**	**<**	**<**	
		**stdev**	**0.2**									**2.0**	**2.1**	**20**			
		**%CV**	**94**									**173**	**177**	**175**			
**25**	**2.5**	**Lab01**	0.4	1.5	4	1.9	2.2	8	5.5	1.6	14	1.4	1.4	6	7.6	3.0	21
**25**	**2.5**	**Lab02**	0.3	3.5	8	4.1	1.0	10	0.5	0.7	2	1.3	1.4	5	2.7	1.4	8
**25**	**2.5**	**Lab03**	0.2	2.0	5	1.0	under		1.5	under		0.9	0.9	4	2.0	1.3	7
**25**	**2.5**	**Lab04**	0.2	under		1.2	1.2	5	2.6	1.3	8	1.0	1.0	4	1.8	1.3	6
**25**	**2.5**	**Lab05**	0.2	under		under	under		under	5.2		2.7	3.5	12	1.9	under	
**25**	**2.5**	**Lab06**	0.3	0.9	2	under	under		3.4	1.0	9	0.6	0.7	3	under	under	
**25**	**2.5**	**Lab07**	1.8	under		16	18	68	16	6.0	44	OVER	OVER		19	9	57
**25**	**2.5**	**Lab08**	0.4	2.7	6	under	under		3.5	1.4	10	1.5	1.4	6	3.4	2.1	11
**25**	**2.5**	**Lab09**	0.4	3.2	7				1.2	under					9.5	under	
**25**	**2.5**	**Lab10**	0.3	under		2.0	3.5	11	under	under		1.0	1.0	4	2.0	under	
**25**	**2.5**	**Lab11**	0.3	under		2.0	2.7	9	3.1	0.9	8	1.9	2.2	8	2.7	1.3	8
		**av**	**0.4**	**<**		**4.0**	**<**		**4.2**	**2.2**	**14**	**1.4**	**1.5**	**6**	**5.3**	**<**	
		**stdev**	**0.5**			**5.3**			**4.7**	**2.1**	**14**	**0.6**	**0.9**	**3**	**5.6**		
		**%CV**	**107**			**133**			**114**	**93**	**103**	**47**	**57**	**52**	**106**		
**100**	**10**	**Lab01**	1.8	5.5	4	8.7	12	11	OVER	8.5		3.6	4.5	4	31	12	22
**100**	**10**	**Lab02**	1.3	4.5	3	18.6	6.1	12	0.3	1.2	1	4.3	5.0	5	9	5	7
**100**	**10**	**Lab03**	1.3	4.1	3	10	15	13	12	4.4	8	3.8	4.6	4	9	6	7
**100**	**10**	**Lab04**	1.6	6.1	4	12	20	16	28	7.6	18	4.3	5.2	5	9	5	7
**100**	**10**	**Lab05**	1.3	5.2	3	13.3	18	16	3.5	7.8	6	6.2	8.3	7	8	5	7
**100**	**10**	**Lab06**	1.6	5.6	4	8.5	10	9	OVER	8.3		0.6	0.7	1	17	under	
**100**	**10**	**Lab07**	9.6	3.0	6	41	54	48	OVER	31		OVER	OVER		67	47	57
**100**	**10**	**Lab08**	1.2	2.3	2	6.4	9	8	8.4	4.0	6	6.0	6.7	6	8	4	6
**100**	**10**	**Lab09**	1.4	2.1	2				6.3	3.5	5				11	under	
**100**	**10**	**Lab10**	1.6	under		17.6	37	27	13.8	1.5	8	4.5	5.7	5	9	6	7
**100**	**10**	**Lab11**	1.3	under		8.5	14	11	11	4.1	8	5.4	6.9	6	7	4	6
		**av**	**2.2**	**4.3**	**3**	**14**	**20**	**17**	**10**	**7.4**	**7**	**4.3**	**5.3**	**5**	**17**	**11**	**14**
		**stdev**	**2.5**	**1.5**	**1**	**10**	**15**	**12**	**8.4**	**8.2**	**5**	**1.7**	**2.1**	**2**	**18**	**14**	**17**
		**%CV**	**112**	**35**	**41**	**71**	**76**	**71**	**81**	**110**	**66**	**39**	**40**	**39**	**106**	**133**	**120**

^*a*^ Interpolated ppm using the calibration standards (S1, S2, S5, S7) in a stepwise, linear analysis of the measured MFI (after subtracting background). S7 defines the upper limit of the dynamic range and 10‐times the standard deviation (10D) of the background (S0) defines the lower limit of quantitation. ppm calculated by converting the interpolated ng extractable protein/mL derived from the calibration standards, to ppm whole allergenic food using the protein content as referenced in Table 1. 'under' and 'OVER' indicate that the measured MFI was either less than or greater than the defined limits of the dynamic range (10D and S7).

^*b*^ μg incurred per gram of food to generate the test portion.

^*c*^ 'Lowest Level' refers to the lowest concentration of analyte that would generate the same analytical sample; by eliminating the optional dilution following extraction.

^*d*^ Incurred analyte and associated bead sets.

^*e*^ 'av % rec' is the average percent recovery by both complementary bead sets; if either is 'under' or 'OVER', no average is reported.

^*f*^ Lab05 did not analyze meat samples due to shipping / import problems. Lab09 failed to monitor bed sets ‐25, ‐26, ‐35, and ‐36 for egg and milk.

^*g*^ 'Over' or 'under' entries are ignored when calculating averages, standard deviations, and %CV values. No entry indicates data insufficient to support calculation.

^*h*^ '<', '>' indicate MFI responses by more than 3 labs either too low ('under') or too high ('over'). 'na' indicates more than 3 labs generating conflicting, out of range data with at leat 2 'under' and 2 'over'.

Further, since a goal of this validation was to appraise xMAP FADA utility when performed by analysts of diverse expertise, poor performing laboratories were not statistically dropped from the analyses. Indeed, to accentuate notation of potential problem areas, if within any sample group (e.g., 1 ppm egg in meat) more than three labs generated MFI outside the dynamic range, no average was calculated and instead noted with either ‘<‘or ‘>‘for the bead set.

In addition, tabulated are the averages of the complementary bead sets. If two complementary bead sets generated comparable ppm levels, it indicates that both bead sets were equally affected. Consistent with the ratio analyses, the interpolated amount of almond in the orange juice samples interpolated to different concentrations with the almond-13 values considerably less than either the almond-12 values or the incurred concentrations of allergenic food. Table 15 tabulates the average calculated concentrations of allergenic food across the 11 participating laboratories, except for when data was not submitted (e.g., Lab 05 did not receive meat samples for analysis, Lab 09 did not collect MFI data for either the egg or milk bead sets). Extending the approach employed in Tables 12–14, when more than three labs generated average MFI outside the dynamic range defined by S0+10D and S7, the fraction of labs outside the dynamic range were indicated with ‘<‘or ‘>‘in place of an average. For example, the detection of 1 ppm egg in meat generated average MFI below the dynamic range for 6 of the 9 labs and therefore the notation ‘6/9<‘in the table. Similarly, 100 ppm milk incurred in orange juice generated for all 11 labs average MFI exceeding S7 and is noted in the table as >11/11. In addition, all data that exceeded the lower limit of the dynamic range, defined by S0+10D, are highlighted in yellow to help illustrate the utility of the xMAP FADA to detect the analytes. As expected, with increasing concentration, the %CV values decreased. Since, several of the 100 μg/g incurred samples of meat, orange juice, and dark chocolate exceeded the dynamic range, the %CV (RSD_R_) values of the 25 μg/g samples were compared. Half of the meat, all orange juice, and one of the dark chocolate bead sets displayed %CV values ≤ 35%. The allergenic foods incurred at 100 μg/g into the baked muffins displayed %CV values ≥ 50% for 7 of the 10 bead sets. The order for worsening %CV of orange juice, meat, dark chocolate, and baked muffins is consistent with the increasing food sample processing and manipulation. Preparation of the orange juice and meat samples entailed only melting the supplied samples and adding extraction buffer, though pipetting of the centrifuged meat extract required avoiding any fat layer. The dark chocolate samples required adding pre-warmed buffer to dissolve the dark chocolate to conduct the extraction. In contrast, the muffin samples entailed baking the incurred allergenic foods and subsequently the analysts had to remove the mini muffins and dice them with a clean razor blade before adding extraction buffer. It is therefore possible that the %CV associated with the recovery of the allergenic foods relate to the degree of processing of the food samples and /or proficiency in analyst handling of the food samples.

**Table 15:** Average interpolated allergenic food concentrations incurred in food samples^a^.

**MEAT**		**EGG** ^c^, ^d^				**GLUTEN**				**MILK**			
		**‐25**		**‐26**		**‐27**		**‐28**		**‐35**		**‐36**	
**μg/g** ^b^	**equiv** ^b^	**ppm**	**%CV**	**ppm**	**%CV**	**ppm**	**%CV**	**ppm**	**%CV**	**ppm**	**%CV**	**ppm**	**%CV**
**1**	**0.1**	**6/9 <**	na	**7/9 <**	na	**2.5**	91	**2.4**	49	**2.8**	108	**3.2**	115
**2.5**	**0.25**	**2.2**	45	**7/9 <**	na	**5.2**	49	**5.6**	41	**2**	134	**4.5**	164
**10**	**1**	**9**	32	**5**	43	**28**	9	**25**	16	**7.2**	103	**6.4**	118
**25**	**2.5**	**22**	32	**27**	53	**> 10/10**	na	**> 10/10**	na	**9**	31	**7**	22
**100**	**10**	**> 4/9**	na	**> 7/9**	na	**> 10/10**	na	**> 10/10**	na	**> 4/9**	na	**15**	67
**ORANGE JUICE**		**ALMOND**				**MILK**				**SOY**			
**μg/g**	**equiv**	**‐12**		**‐13**		**‐35**		**‐36**		**‐45**		**‐46**	
		**ppm**	**%CV**	**ppm**	**%CV**	**ppm**	**%CV**	**ppm**	**%CV**	**ppm**	**%CV**	**ppm**	**%CV**
**1**	**0.1**	**0.7**	20	**5/11 <**	na	**1.6**	181	**4.9**	166	**8/11 <**	na	**10/11 <**	na
**2.5**	**0.25**	**1.6**	19	**0.5**	18	**3.1**	149	**3.8**	156	**4/10 <**	na	**9/11 <**	na
**10**	**1**	**8.7**	41	**2.1**	44	**9.5**	74	**9.6**	81	**6**	107	**6.6**	89
**25**	**2.5**	**21**	15	**4**	32	**> 4/11**	na	**21**	26	**10**	23	**9.8**	18
**100**	**10**	**> 4/11**	na	**15**	32	**> 11/11**	na	**> 11/11**	na	**41**	13	**39**	15
**BAKED MUFFIN**		**COCONUT**				**EGG**				**GLUTEN**			
**μg/g**	**equiv**	**‐20**		**‐21**		**‐25**		**‐26**		**‐27**		**‐28**	
		**ppm**	**%CV**	**ppm**	**%CV**	**ppm**	**%CV**	**ppm**	**%CV**	**ppm**	**%CV**	**ppm**	**%CV**
**1**	**0.1**	**10/11 <**	na	**9/11 <**	na	**8/10 <**	na	**9/10 <**	na	**10/11 <**	na	**9/11 <**	na
**2.5**	**0.25**	**7/11 <**	na	**11/11 <**	na	**8/10 <**	na	**9/10 <**	na	**5/11 <**	na	**9/11 <**	na
**10**	**1**	**0.2**	94	**7/11 <**	na	**6/10 <**	na	**7/10 <**	na	**6/11 <**	na	**6/11 <**	na
**25**	**2.5**	**0.4**	107	**5/11 <**	na	**4**	133	**4/10 <**	na	**4.2**	114	**2.2**	93
**100**	**10**	**2.2**	112	**4.3**	35	**14**	71	**15**	76	**10**	81	**7.4**	110
**DARK CHOCOLATE**		**HAZELNUT**				**PEANUT**							
**μg/g**	**equiv**	**‐29**		**‐30**		**‐37**		**‐38**					
		**ppm**	**%CV**	**ppm**	**%CV**	**ppm**	**%CV**	**ppm**	**%CV**				
**1**	**0.2**	**6/11 <**	na	**5/11 <**	na	**6/11 <**	na	**9/11 <**	na				
**2.5**	**0.5**	**4/11 <**	na	**4/11 <**	na	**6/11 <**	na	**9/11 <**	na				
**10**	**2**	**5.8**	38	**4.4**	43	**1**	55	**0.5**	47				
**25**	**2.5**	**14**	35	**12**	46	**2.5**	57	**1.8**	72				
**100**	**20**	**> 6/11**	na	**> 5/11**	na	**6.7**	32	**4.6**	35				
**MEAT**		**SOY**											
		**‐45**		**‐46**									
**μg/g** ^b^	**equiv** ^b^	**ppm**	**%CV**	**ppm**	**%CV**								
**1**	**0.1**	**0.7**	123	**1.3**	65								
**2.5**	**0.25**	**1.1**	50	**1.1**	68								
**10**	**1**	**4.5**	56	**3.6**	53								
**25**	**2.5**	**11**	56	**9**	60								
**100**	**10**	**51**	88	**41**	90								
**ORANGE JUICE**													
**μg/g**	**equiv**												
**1**	**0.1**												
**2.5**	**0.25**												
**10**	**1**												
**25**	**2.5**												
**100**	**10**												
**BAKED MUFFIN**		**MILK**	**WALNUT**										
**μg/g**	**equiv**	**‐35**		**‐36**		**‐47**		**‐48**					
		**ppm**	**%CV**	**ppm**	**%CV**	**ppm**	**%CV**	**ppm**	**%CV**				
**1**	**0.1**	**7/9 <**	na	**8/10 <**	na	**9/11 <**	na	**9/11 <**	na				
**2.5**	**0.25**	**1.3**	145	**2.1**	125	**8/11 <**	na	**9/11 <**	na				
**10**	**1**	**1.2**	173	**1.2**	177	**5/11 <**	na	**5/11 <**	na				
**25**	**2.5**	**1.4**	47	**1.5**	57	**5.3**	106	**4/11 <**	na				
**100**	**10**	**4.3**	39	**5.3**	40	**17**	106	**11**	133				
**DARK CHOCOLATE**													
**μg/g**	**equiv**												
**1**	**0.2**												
**2.5**	**0.5**												
**10**	**2**												
**25**	**2.5**												
**100**	**20**												

^*a*^ Concentrations (ppm) derived from ng protein/mL calculated from calibration standards S0, S1, S2, S5, and S7 in PBST (UD Buffer for chocolate) assuming linearity between the different calibrants and using 10‐times the standard deviation of the background (S0+10D) as a lower limit of quantitation. Conversion from interpolated ng protein/mL based on analyte protein content with the calculated ppm averaged across participating laboratories. All samples were extracted in triplicate and the MFI, after subtraction of the background (S0), to calculate ppm of analyte.

^*b*^ 'μg/g' incurred into a gram of food, 'equiv' is comparable concentration of analyte that would generate similar responses by omitting optional dilution of extract (10X for PBST and 5X for UD Buffer).

^*c*^ If more than three of the contributing laboratories generated responses outside the dynamic range, the fraction of such labs is reported. Specifically, '6/9 <' indicates 6 of 9 below 10‐time the standard deviation of the background (10D) and '> 4/9' indicates 4 of 9 above S7.

^*d*^ Light yellow highlight indicates detection of analyte more than the lower limit S0+10D.

The percent recoveries are presented in Table 16 with those instances where more than three labs were outside the dynamic range indicated by the fraction outside the dynamic range. The average percent recoveries were generally consistent with what was observed in the single lab validation and typically observed with ELISAs employing buffered-detergent extraction protocols. The baked muffins displayed the lowest levels of recovery, followed by the chocolate samples, the orange juice samples, and lastly the meat samples. The baked muffins averaged 10% for incurred levels of 10, 25, and 100 μg/g. This percent recovery was approximately one-third the level observed in the single lab validation for baked muffins (33%). This may be due to a combination of less experienced analysts and differences in preparing the muffins for extraction (e.g., degree of dicing). The chocolate samples displayed average recoveries for hazelnut greater than peanut of 57% and 9%, respectively. In the single lab validation, the two analytes behaved comparably with an average percent recovery for all analytes of approximately 12% for the chocolate incurred samples relative to the allergenic foods extracted from PBST samples. The reproducibly higher recovery of hazelnut versus peanut in the MLV may reflect a feature of this particular sample since a similar elevated recovery was not observed in the single lab validation [11]. Recoveries were excellent for the meat and orange samples except for almond-13 which were 15–20%; about one-quarter the recoveries observed with almond-12 and consistent with the change in the almond-13/almond-12 ratio.

**Table 16:** Percent allergenic food recovered from food samples^a^.

**MEAT**		**EGG** ^c^, ^d^		**GLUTEN**		**MILK**		**SOY**			
		**‐25**	**‐26**	**‐27**	**‐28**	**‐35**	**‐36**	**‐45**	**‐46**		
**μg/g** ^b^	**equiv** ^b^	**% Recovery**	**% Recovery**	**% Recovery**	**% Recovery**	**% Recovery**	**% Recovery**	**% Recovery**	**% Recovery**		
**1**	**0.1**	**6/9 <**	**7/9 <**	**250**	**240**	**280**	**320**	**70**	**130**		
**2.5**	**0.25**	**88**	**7/9 <**	**208**	**224**	**80**	**180**	**44**	**44**		
**10**	**1**	**90**	**50**	**280**	**250**	**72**	**64**	**45**	**36**		
**25**	**2.5**	**88**	**108**	**> 10/10**	**> 10/10**	**36**	**28**	**44**	**36**		
**100**	**10**	**> 4/9**	**> 7/9**	**> 10/10**	**> 10/10**	**> 4/9**	**15**	**51**	**41**		
**ORANGE JUICE**		**ALMOND**		**MILK**		**SOY**					
		**‐12**	**‐13**	**‐35**	**‐36**	**‐45**	**‐46**				
**μg/g**	**equiv**	**% Recovery**	**% Recovery**	**% Recovery**	**% Recovery**	**% Recovery**	**% Recovery**				
**1**	**0.1**	**70**	**5/11 <**	**160**	**490**	**8/11 <**	**10/11 <**				
**2.5**	**0.25**	**64**	**20**	**124**	**152**	**4/10 <**	**9/11 <**				
**10**	**1**	**87**	**21**	**95**	**96**	**60**	**66**				
**25**	**2.5**	**84**	**16**	**> 4/11**	**84**	**40**	**39**				
**100**	**10**	**> 4/11**	**15**	**> 11/11**	**> 11/11**	**41**	**39**				
**BAKED MUFFIN**		**COCONUT**		**EGG**		**GLUTEN**		**MILK**		**WALNUT**	
		**‐20**	**‐21**	**‐25**	**‐26**	**‐27**	**‐28**	**‐35**	**‐36**	**‐47**	**‐48**
**μg/g**	**equiv**	**% Recovery**	**% Recovery**	**% Recovery**	**% Recovery**	**% Recovery**	**% Recovery**	**% Recovery**	**% Recovery**	**% Recovery**	**% Recovery**
**1**	**0.1**	**10/11 <**	**9/11 <**	**8/10 <**	**9/10 <**	**10/11 <**	**9/11 <**	**7/9 <**	**8/10 <**	**9/11 <**	**9/11 <**
**2.5**	**0.25**	**7/11 <**	**11/11 <**	**8/10 <**	**9/10 <**	**5/11 <**	**9/11 <**	**52**	**84**	**8/11 <**	**9/11 <**
**10**	**1**	**2**	**7/11 <**	**6/10 <**	**7/10 <**	**6/11 <**	**6/11 <**	**12**	**12**	**5/11 <**	**5/11 <**
**25**	**2.5**	**2**	**5/11 <**	**16**	**4/10 <**	**17**	**9**	**6**	**6**	**21**	**4/11 <**
**100**	**10**	**2**	**4**	**14**	**15**	**10**	**7**	**4**	**5**	**17**	**11**
**DARK CHOCOLATE**		**HAZELNUT**		**PEANUT**							
		**‐29**	**‐30**	**‐37**	**‐38**						
**μg/g**	**equiv**	**% Recovery**	**% Recovery**	**% Recovery**	**% Recovery**						
**1**	**0.2**	**6/11 <**	**5/11 <**	**6/11 <**	**9/11 <**						
**2.5**	**0.5**	**4/11 <**	**4/11 <**	**6/11 <**	**9/11 <**						
**10**	**2**	**58**	**44**	**10**	**5**						
**25**	**2.5**	**56**	**48**	**10**	**7**						
**100**	**20**	**> 6/11**	**> 5/11**	**7**	**5**						

^*a*^ Percent recoveries based on calculated ppm divided by the amount of allergenic food incurred in the food samples.

^*b*^ 'μg/g' incurred into a gram of food, 'equiv' is comparable concentration of analyte that would generate similar responses by omitting optional dilution of extract (10X for PBST and 5X for UD Buffer).

^*c*^ If more than three of the contributing laboratories generated responses outside the dynamic range, the fraction of such labs is reported. Specifically, '6/9 <' indicates 6 of 9 below 10‐time the standard deviation of the background (10D) and '> 4/9' indicates 4 of 9 above S7.

^*d*^ Light yellow highlight indicates detection of analyte more than the lower limit S0+10D.

### Direct comparison controls (DCCs)

Direct comparison controls (DCCs) provide a second measure of analyst proficiency, assay performance, as well as a direct tool for ascertaining whether a food sample contains a target analyte at amounts exceeding a specified level. The DCCs are prepared on the day of analysis by spiking the specified allergenic food into analyte-free samples and analyzing the samples alongside. As such, DCCs avoid any assumptions and conversion factors associated with interpolating analyte concentrations from the calibration standards. Specifically, the DCCs can be prepared by spiking the analyte-free food with the allergenic food as may be inadvertently present in the food samples. This is exemplified by using suspensions of ground raw hazelnut, not a protein extract as employed in the calibration standards. Further, since the detection of spiked allergenic food typically displays higher levels of recovery versus incurred analyte, the observation of MFI characteristic of the analyte and greater than the DCC indicates a definitively greater amount of analyte in the food sample.

The DCCs are handled and extracted alongside the food samples. As such, DCCs also provide a measure of the analyst’s ability to perform the extraction protocol. DCCs also serve as controls for matrix effects, supplementing the roles played by the built-in redundancy, multiplex design, and AssayChex^™^ bead sets included in the bead set cocktail. Tables 4 and 5 present the MFI and S/N-1 data for DCCs containing 20 ppm gluten (G20), 10 ppm peanut (P10), and 50 ppm NFDM (M50) analyzed using reagents from Lot 5. Though 20 ppm intact gluten is a regulatory threshold used to define ‘gluten-free’ to safeguard consumers with Celiac Disease, in the USA thresholds have not been established for food allergens. It is envisioned that if threshold levels are adopted, such would influence the amounts of allergenic food used to make the DCCs. Further, the DCCs employed in an experiment would be chosen to represent the allergenic foods of interest; for example, if testing for tree nuts, DCC’s of almond, hazelnut, and walnut might be employed.

The DCC MFI data tabulated in Tables 4 and 5 are the average of DCCs prepared in four different foods, each in triplicate. The meat, orange juice, and baked muffin samples were extracted (1:20) using the PBST Buffered-detergent Protocol and diluted 10-fold prior to mixing with the bead cocktail (net, 200-folddilution). The dark chocolate samples were extracted (1:40) according to the UD Buffer Protocol and diluted 5-fold with UD Buffer prior to analysis (net, 200-fold). Inasmuch as UD Buffer contains milk, the dark chocolate samples were not included in the M50 data sets. As expected, the gluten, peanut, and milk DCCs generated intense responses with the gluten, peanut, and milk bead sets, respectively with virtually no significant responses with the other bead sets. As a result, the S/N-1 data in Table 5 are zeros for all bead sets except the spiked analyte. Not surprisingly, the milk DCC displayed the largest S/N-1 consistent with its 50 ppm concentration being considerably greater than the concentration of gluten (20 ppm) and peanut (10 ppm) in the other two types of DCC with differences between the complementary bead sets as previously observed (see section on ratio analysis above and discussions regarding analyte, bead-specific sensitivities in Cho et al. 2015 [5]).

The MFI observed for the various food samples compared to the MFI response generated by the appropriate DCC are depicted in a series of figures; for gluten bead sets -27 and -28 compared to G20 (S2 Fig), milk bead sets -35 and -36 compared to M50 (S3 Fig), and peanut bead sets -37 and -38 compared to P10 (S4 Fig). The data for each participant is plotted on separate graphs with the results obtained with the meat (red), orange juice (blue), baked muffin (green) and dark chocolate (purple) presented as the difference after subtracting the MFI generated by the DCC. The only exceptions were omission of the dark chocolate samples with bead sets -35 and -36 due to the presence of milk in the UD extraction buffer, meat analyses were not conducted by Lab 05 and Lab 09 did not collect data for bead sets -35 and -36. The detection of an analyte is indicated by a positive slope with crossing of the abscissa expected for samples incurred at levels greater than the amount spiked into the food (by the analyst) to make the DCCs.

S2 Fig depicts the MFI by gluten bead sets -27 and -28 minus the responses generated by the 20 ppm gluten DCCs (G20). All participants correctly detected the presence of gluten in the meat and baked muffin samples with all, but one of the labs, correctly displaying flat lines for the orange juice and dark chocolate samples; ten of the dark chocolate data points properly overlapped the abscissa for gluten-27. Further, eight of the 10 labs displayed appropriate transitions over the abscissa for the meat samples with gluten-27 and six correctly crossed the abscissa for gluten-28, another two being shifted. Reduced recovery has been extensively documented with baked food samples. It was therefore not surprising that the baked muffin samples displayed less intense MFI and only crossed the abscissa appropriately six times with gluten-27 and was greatly shifted in the gluten-28 plots.

Milk was incurred into all samples, but since the dark chocolate was extracted using UD buffer, it was not included in the plots for milk bead sets -35 and -36. Also, one of the labs inadvertently failed to monitor bead sets -35 and -36; therefore, there were only 10 participants for these analyses. Seven of the 10 labs correctly indicated the presence of milk using bead set -35; eight correctly indicated the presence of milk using bead set -36 (S3 Fig). Only three labs displayed results with the meat and orange juice samples appropriately crossing the abscissa. Not surprisingly, none of the baked muffin samples crossed the abscissa and for eight of the ten labs, the baked muffin samples displayed poorer performance than the meat or orange juice samples, with the meat and orange juice data overlapping six times.

Peanut was only incurred into the dark chocolate samples. All 11 of the laboratories correctly detected the presence of peanut in the dark chocolate with seven appropriately crossing the abscissa for peanut bead sets -37 and -38 (S4 Fig). Interestingly, three of the labs displayed slight increases for either the orange juice or baked muffin samples. Otherwise, the eight remaining labs displayed no significant responses for the other food samples, with four of the labs properly displaying indistinguishable flat lines for the meat, orange juice, and baked muffin samples with bead set -37, five labs with bead set -38.

Overall the analysts were able to successfully perform the DCC part of the MLV with only a few minor errors, possibly related to the complexity associated with preparing the DCCs (see INSTR file, pages on DCC preparation). A question critical for the end-user to address is the level of proficiency desired which relates to quantitative variance. The lower quantitative performance of the baked muffin samples is in agreement with the reduced recovery typically observed with baked products using buffered-detergent extraction protocols as observed for the xMAP FADA (above, single lab validation) and commercial ELISA test kits. Despite this variability, the DCCs provided an excellent tool for direct qualitative detection and ascertaining whether a sample contained analyte in excess of a specified target level.

### Overview

The xMAP FADA displayed excellent performance reliability despite the MLV entailing 9 out of 11 participants not experienced in the assay. Absolute differences in MFI values were observed but intra-lab variability was low and comparable to ELISA analysis. Further, despite sometimes high levels of variance in the inter-lab data, the strong signal-to-noise enabled reliable qualitative detection and avoidance of false negatives. Specifically, the excellent signal-to-noise at the start of the dynamic range and its large increase across the dynamic range for each bead set made erroneous false negatives due to variance in the data less likely. Further, ratio analyses (i.e., ratios between complementary bead sets and multi-antibody profiling) distinguished between target analytes and cross-reactive homologues. Inter-lab variances of the ratio analyses varied with the type of incurred food samples. The worst (largest) variances were observed with the baked muffin samples. The baked muffin samples containing 100 μg/g of the allergenic foods averaged %CV (RSD_R_) values of 26% in the ratio analyses and displayed an average recovery of 9% ±5% (n = 10 bead sets). In contrast, the allergenic foods incurred at 100 μg/g in the meat, orange juice, and chocolate samples displayed %CV values in the ratio analyses of 15%, 15%, and 17%, respectively. Recovery patterns were comparable to those published in the single lab validation and basically comparable to what has been typically observed with ELISA test kits that employ buffered-detergent extraction protocols [11, 17]. The baked muffins displayed the poorest recoveries and though only approximately 10%, still generated highly reproducible MFI. Thus, the five incurred allergenic foods were reliably detected except for two labs generating questionable results for gluten and two labs not observing confirmatory responses with coconut-21 and walnut-48. The only potentially serious concern was the inability of the labs to generate MFI in excess of the S1 calibration standard for both walnut bead sets (primarily-48); however, the MFI did exceed the S0+10D in all but two instances for walnut-48, and displayed an appropriate increase in intensity with concentration. It is not clear whether the poor sensitivity in detecting walnut was entirely due to baking or in part related to the preparation of the walnut stock and subsequent sub-stock solutions and the inability to generate a ground powder to generate uniform suspensions. Lastly, preparation of the samples included an optional dilution of the food extracts. Thus, the concentration of analyte in the analytical samples could also be generated from samples containing 0, 0.1, 0.25, 1, 2.5, and 10 μg/g (0.2, 0.5, 2, 5, and 20 μg/g for the chocolate samples) with minimal changes in matrix carry over. As such, the data collected in the MLV represents worse case scenarios since it is expected that typically samples exceeding 10 ppm will be the focus of any analyses and the optional dilution step can be omitted.

The xMAP FADA is currently the only commercial antibody-based method capable of reliably detecting and distinguishing between allergenic foods and cross-reactive homologues for seven of the ‘big+ eight’ classes of food allergens regulated in the USA and many other countries. The xMAP FADA was also multi-lab validated for its ability to quantify detected allergenic foods and using DCCs can provide a second measure of whether an allergenic food was present at concentrations exceeding a chosen target level. Variability in performance with the DCCs was greater than typically observed with experienced analysts and probably reflects the complexity in preparing the DCCs which basically doubles the assay time of < 4 hrs. Despite this variability, the xMAP was successfully performed by all participants despite the lack of expertise. This does not mean that it is not necessary to maximize the proficiency of analysts performing the xMAP FADA, instead all effort should be made to maximize proficiency and hence assay capability. Lastly, by virtue of its modular design the xMAP FADA is compatible to changes in the repertoire to meet special and future needs.

## Supporting information

S1 Fig Lab specific average calibration curves. (PDF)

S2 Fig Comparisons between the MFI generated by the incurred food samples and the 20 ppm gluten DCCs (G20). (PDF)

S3 Fig Comparisons between the MFI generated by the incurred food samples and the 50 ppm milk DCCs (M50). (PDF)

S4 Fig Comparisons between the MFI generated by the incurred food samples and the 10 ppm peanut DCCs (P10). (PDF)

S1 Appendix Instructional items supplied to the laboratories. (PDF)
