# Supplementary material for: Multi-laboratory validation of the xMAP—Food Allergen Detection Assay: A multiplex, antibody-based assay for the simultaneous detection of food allergens
Source: PLoS One. 2020 Jul 9;15(7):e0234899. doi: 10.1371/journal.pone.0234899 (PMC7347184; doi:10.1371/journal.pone.0234899)
Supplement: S1 Fig — (PDF) [file pone.0234899.s001.pdf]

S1 FIGURE

S1 Figure. Lab Specific Average Calibration Curves. Plots of the averages of 4 experiments (3 PBST and 1 UD buffer) for each participating laboratory. A – K represent Labs 01 - 11, respectively. The calibration curves are grouped by complementary antibody bead sets with the lower numerical bead set represented by a solid line and the higher numbered bead set by a dashed line. Each data point represents the average of 4* sets of triplicate analyses. Error bars represent one standard deviation. *Lab 05 based on three data sets (2 PBST and 1 UD buffer), Lab 09 did not collect data for egg (-25, -26) or milk (-35, -36) bead sets.

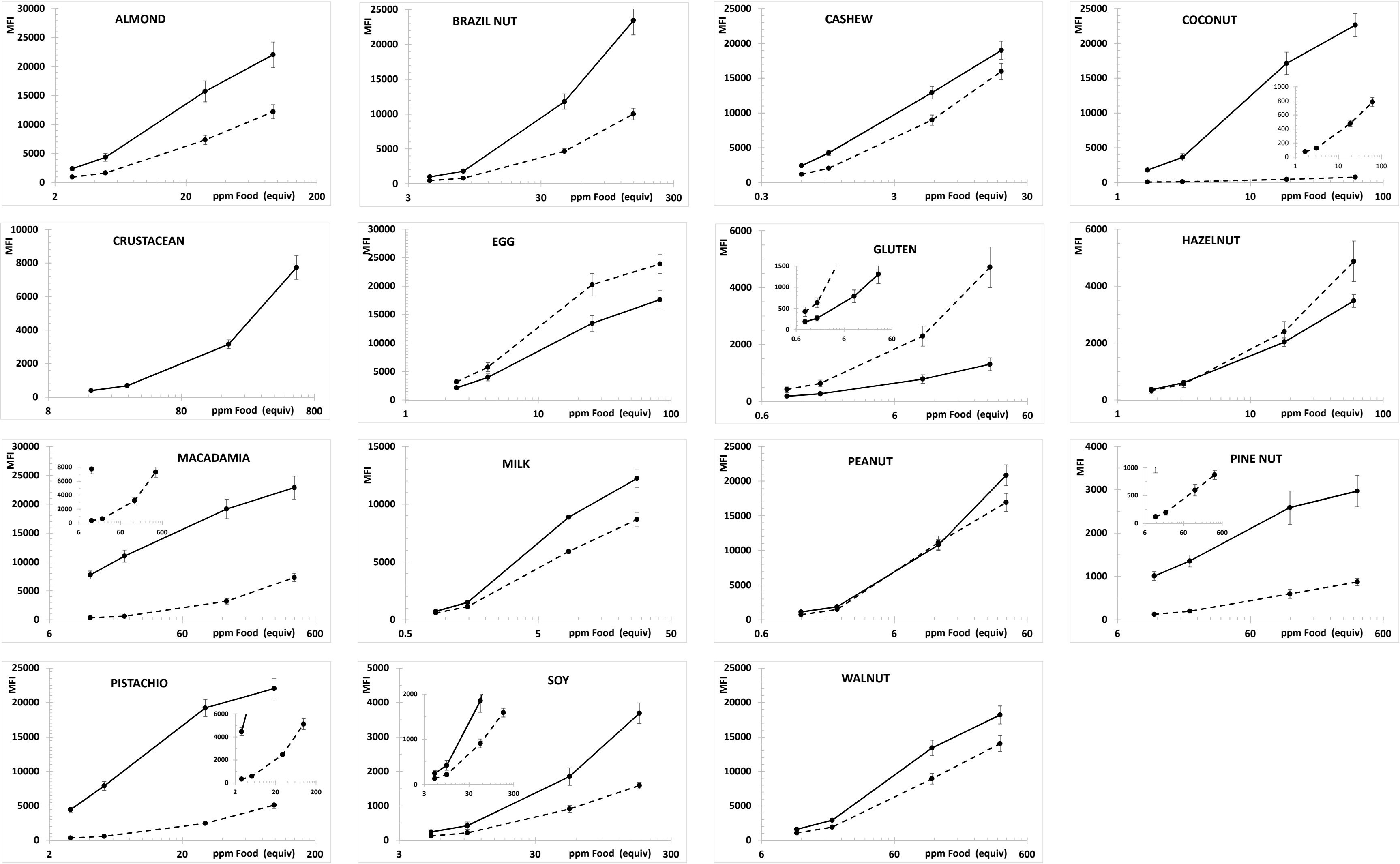
ALMOND
BRAZIL NUT
CASHEW
COCONUT
CRUSTACEAN
EGG
GLUTEN
HAZELNUT
MACADAMIA
MILK
PEANUT
PINE NUT
PISTACHIO
SOY
WALNUT
MFI
ppm Food (equiv)

Figure A

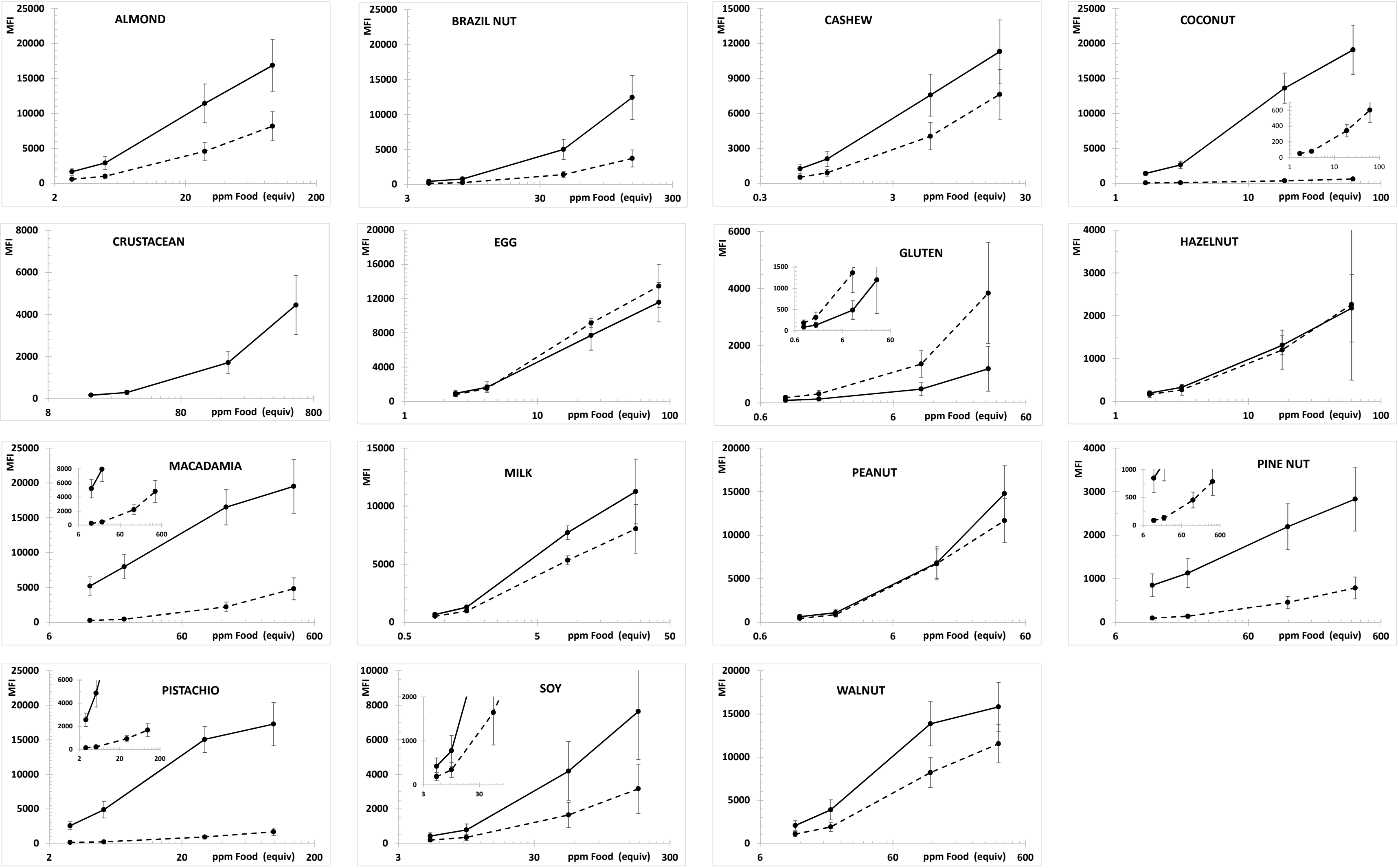
ALMOND
BRAZIL NUT
CASHEW
COCONUT
CRUSTACEAN
EGG
GLUTEN
HAZELNUT
MACADAMIA
MILK
PEANUT
PINE NUT
PISTACHIO
SOY
WALNUT
MFI
ppm Food (equiv)

Figure B

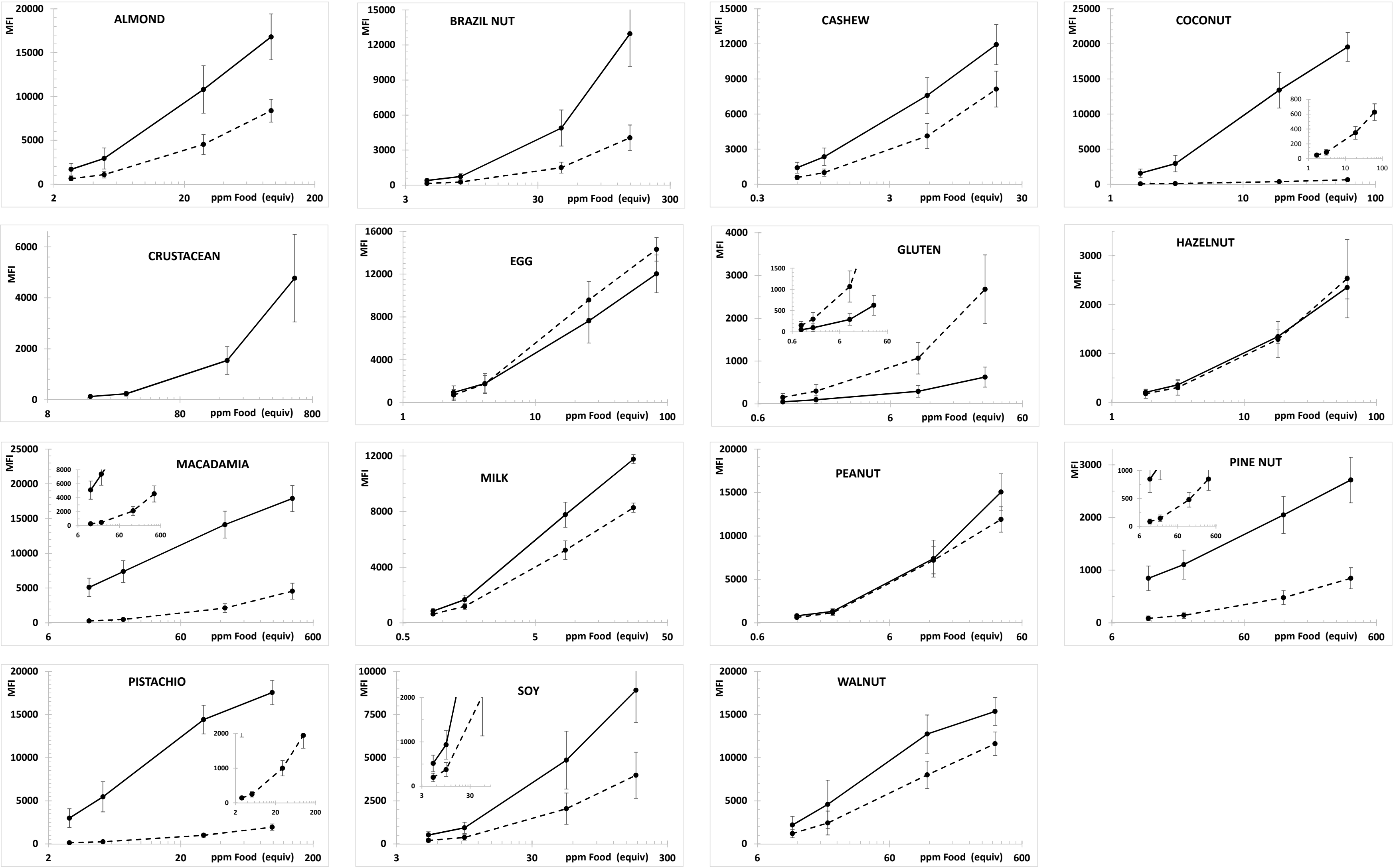
ALMOND
BRAZIL NUT
CASHEW
COCONUT
CRUSTACEAN
EGG
GLUTEN
HAZELNUT
MACADAMIA
MILK
PEANUT
PINE NUT
PISTACHIO
SOY
WALNUT
MFI
ppm Food (equiv)

Figure C

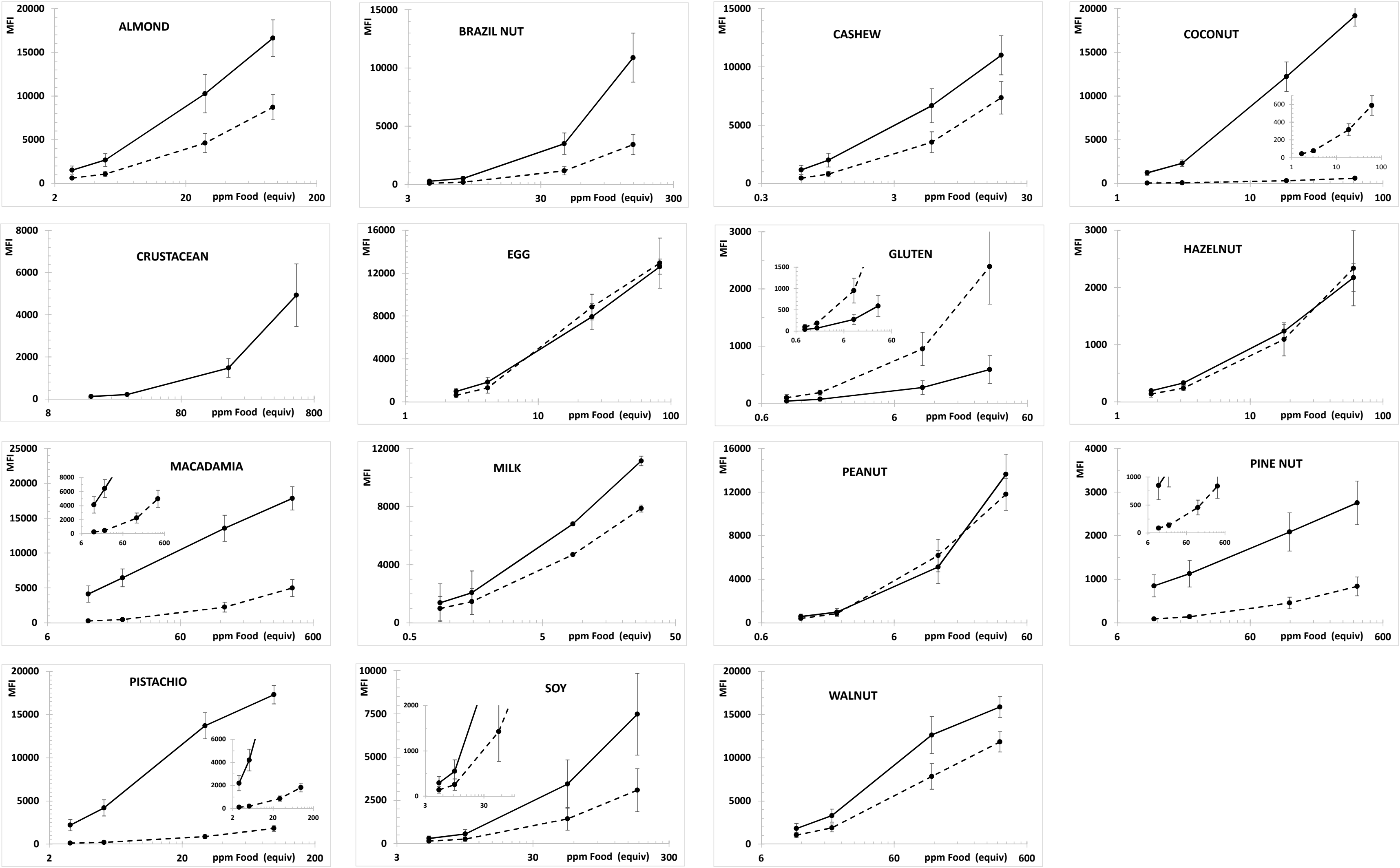

ALMOND
BRAZIL NUT
CASHEW
COCONUT
CRUSTACEAN
EGG
GLUTEN
HAZELNUT
MACADAMIA
MILK
PEANUT
PINE NUT
PISTACHIO
SOY
WALNUT
MFI
ppm Food (equiv)

Figure D

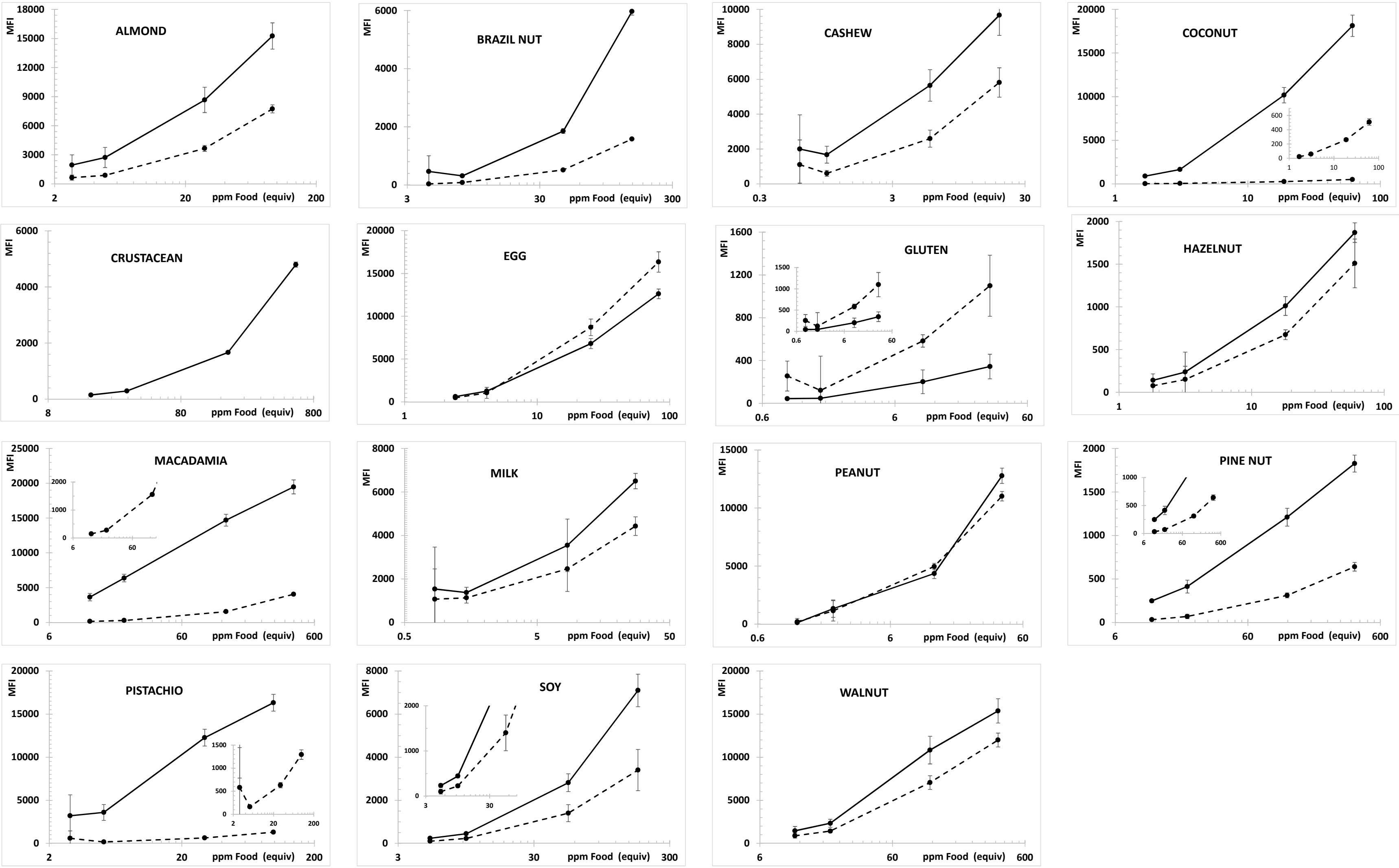
ALMOND
BRAZIL NUT
CASHEW
COCONUT
CRUSTACEAN
EGG
GLUTEN
HAZELNUT
MACADAMIA
MILK
PEANUT
PINE NUT
PISTACHIO
SOY
WALNUT
MFI
ppm Food (equiv)

Figure E

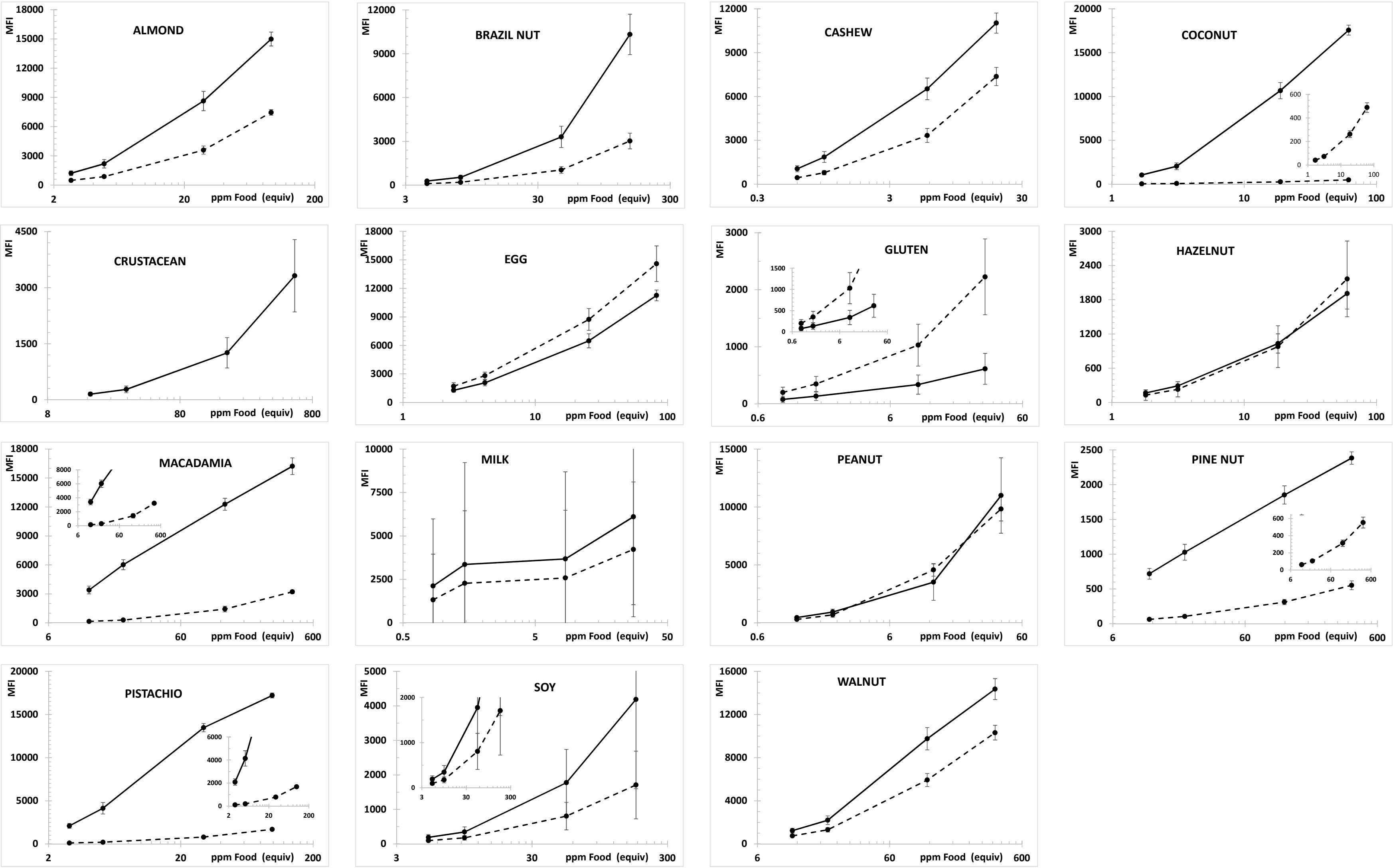
ALMOND
MFI
ppm Food (equiv)
BRAZIL NUT
CASHEW
COCONUT
CRUSTACEAN
EGG
GLUTEN
HAZELNUT
MACADAMIA
MILK
PEANUT
PINE NUT
PISTACHIO
SOY
WALNUT

Figure F

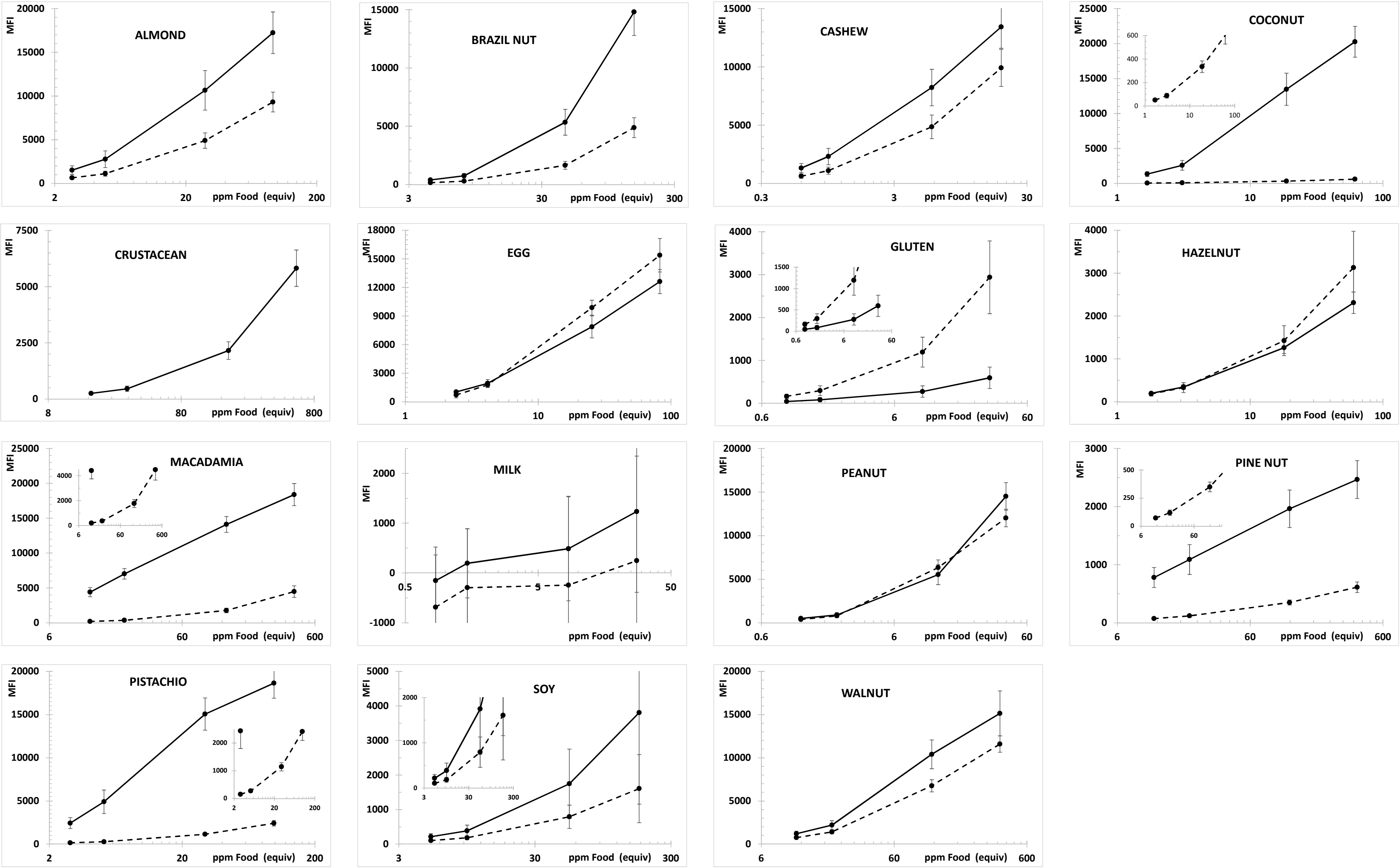

ALMOND
BRAZIL NUT
CASHEW
COCONUT
CRUSTACEAN
EGG
GLUTEN
HAZELNUT
MACADAMIA
MILK
PEANUT
PINE NUT
PISTACHIO
SOY
WALNUT
MFI
ppm Food (equiv)

Figure G

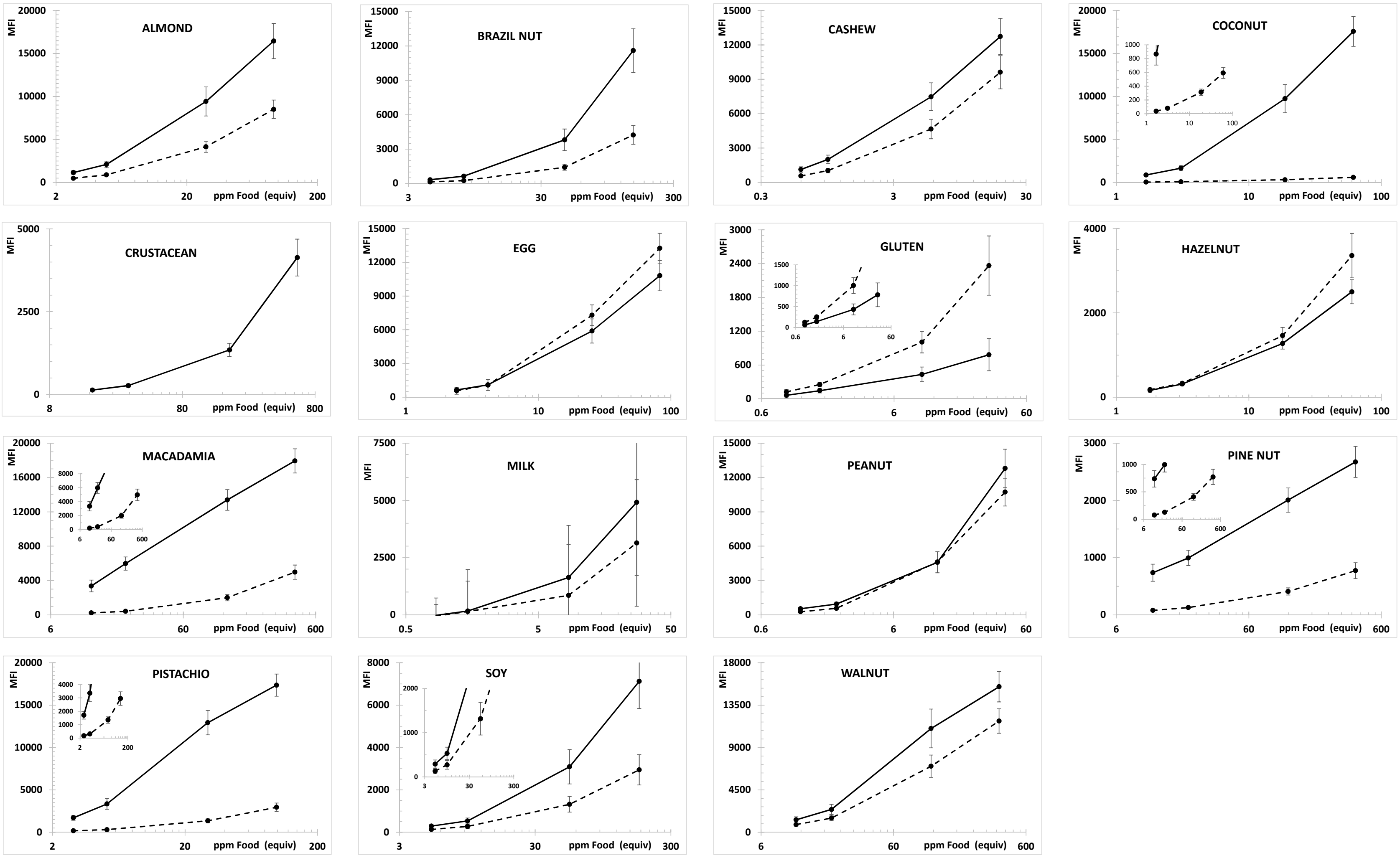
ALMOND
MFI
20000 15000 10000 5000 0
2 20 ppm Food (equiv) 200
BRAZIL NUT
MFI
15000 12000 9000 6000 3000 0
3 30 ppm Food (equiv) 300
CASHEW
MFI
15000 12000 9000 6000 3000 0
0.3 3 ppm Food (equiv) 30
COCONUT
MFI
20000 15000 10000 5000 0
1 10 ppm Food (equiv) 100
CRUSTACEAN
MFI
5000 2500 0
8 80 ppm Food (equiv) 800
EGG
MFI
15000 12000 9000 6000 3000 0
1 10 ppm Food (equiv) 100
GLUTEN
MFI
3000 2400 1800 1200 600 0
0.6 6 ppm Food (equiv) 60
HAZELNUT
MFI
4000 2000 0
1 10 ppm Food (equiv) 100
MACADAMIA
MFI
20000 16000 12000 8000 4000 0
6 60 ppm Food (equiv) 600
MILK
MFI
7500 5000 2500 0
0.5 5 ppm Food (equiv) 50
PEANUT
MFI
15000 12000 9000 6000 3000 0
0.6 6 ppm Food (equiv) 60
PINE NUT
MFI
3000 2000 1000 0
6 60 ppm Food (equiv) 600
PISTACHIO
MFI
20000 15000 10000 5000 0
2 20 ppm Food (equiv) 200
SOY
MFI
8000 6000 4000 2000 0
3 30 ppm Food (equiv) 300
WALNUT
MFI
18000 13500 9000 4500 0
6 60 ppm Food (equiv) 600

Figure H

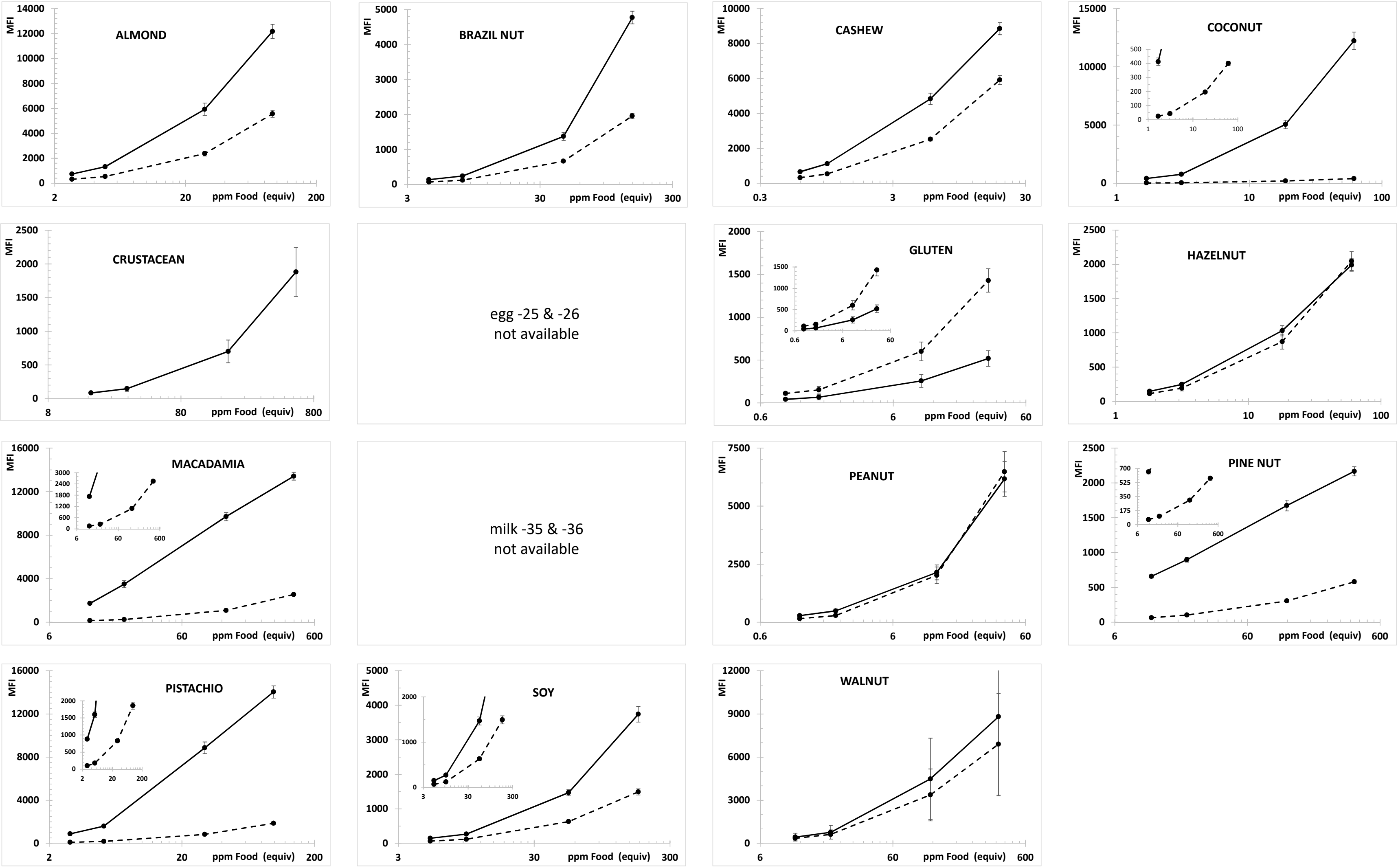
ALMOND
BRAZIL NUT
CASHEW
COCONUT
CRUSTACEAN
egg -25 & -26
not available
GLUTEN
HAZELNUT
MACADAMIA
milk -35 & -36
not available
PEANUT
PINE NUT
PISTACHIO
SOY
WALNUT
MFI
ppm Food (equiv)

Figure I

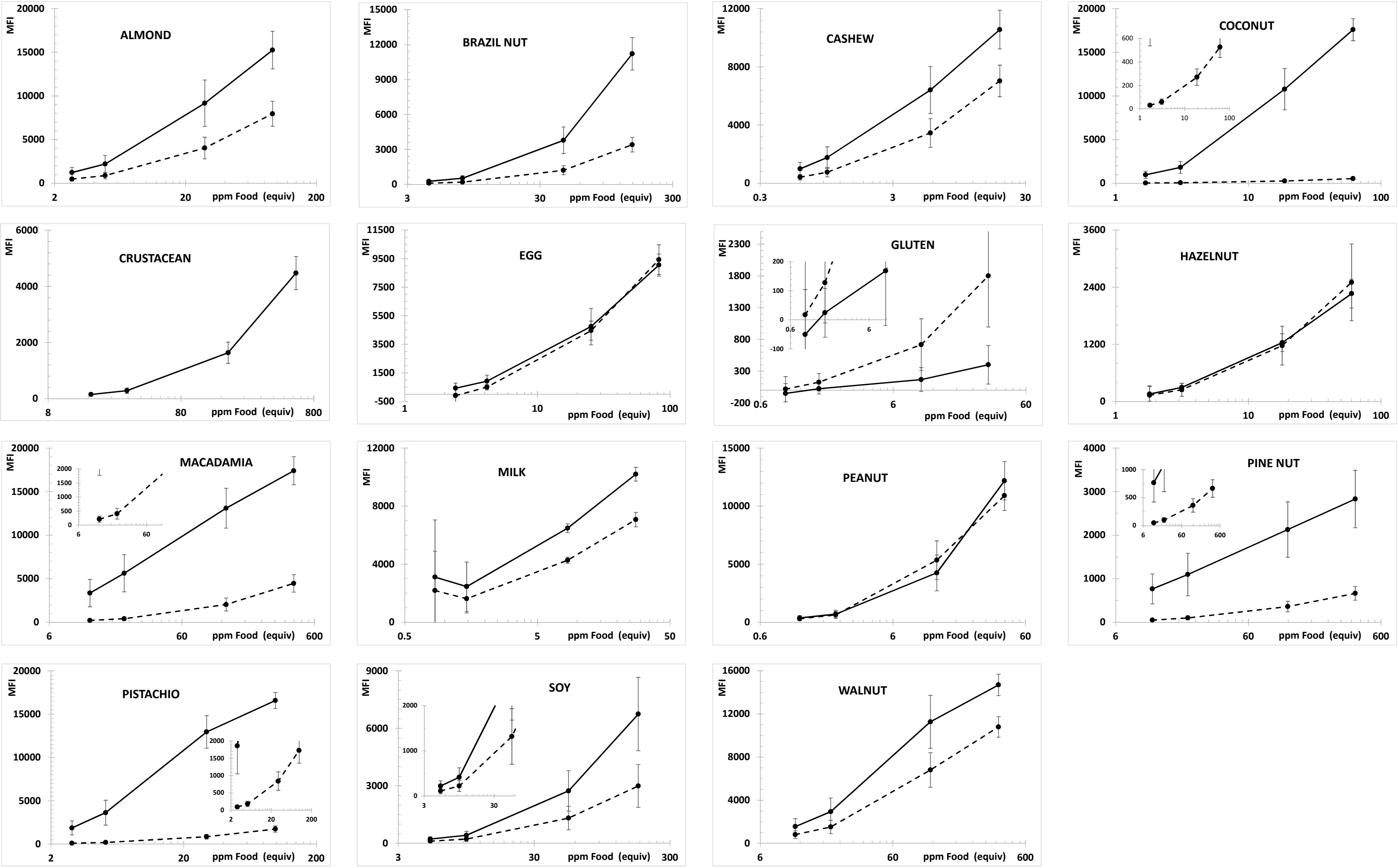
ALMOND
MFI
20000
15000
10000
5000
0
2
20
200
ppm Food (equiv)
BRAZIL NUT
MFI
15000
12000
9000
6000
3000
0
3
30
300
ppm Food (equiv)
CASHEW
MFI
12000
8000
4000
0
0.3
3
30
ppm Food (equiv)
COCONUT
MFI
20000
15000
10000
5000
0
1
10
100
ppm Food (equiv)
CRUSTACEAN
MFI
6000
4000
2000
0
8
80
800
ppm Food (equiv)
EGG
MFI
11500
9500
7500
5500
3500
1500
-500
1
10
100
ppm Food (equiv)
GLUTEN
MFI
2300
1800
1300
800
300
-200
0.6
6
60
ppm Food (equiv)
HAZELNUT
MFI
3600
2400
1200
0
1
10
100
ppm Food (equiv)
MACADAMIA
MFI
20000
15000
10000
5000
0
6
60
600
ppm Food (equiv)
MILK
MFI
12000
8000
4000
0
0.5
5
50
ppm Food (equiv)
PEANUT
MFI
15000
10000
5000
0
0.6
6
60
ppm Food (equiv)
PINE NUT
MFI
4000
3000
2000
1000
0
6
60
600
ppm Food (equiv)
PISTACHIO
MFI
20000
15000
10000
5000
0
2
20
200
ppm Food (equiv)
SOY
MFI
9000
6000
3000
0
3
30
300
ppm Food (equiv)
WALNUT
MFI
16000
12000
8000
4000
0
6
60
600
ppm Food (equiv)

Figure J

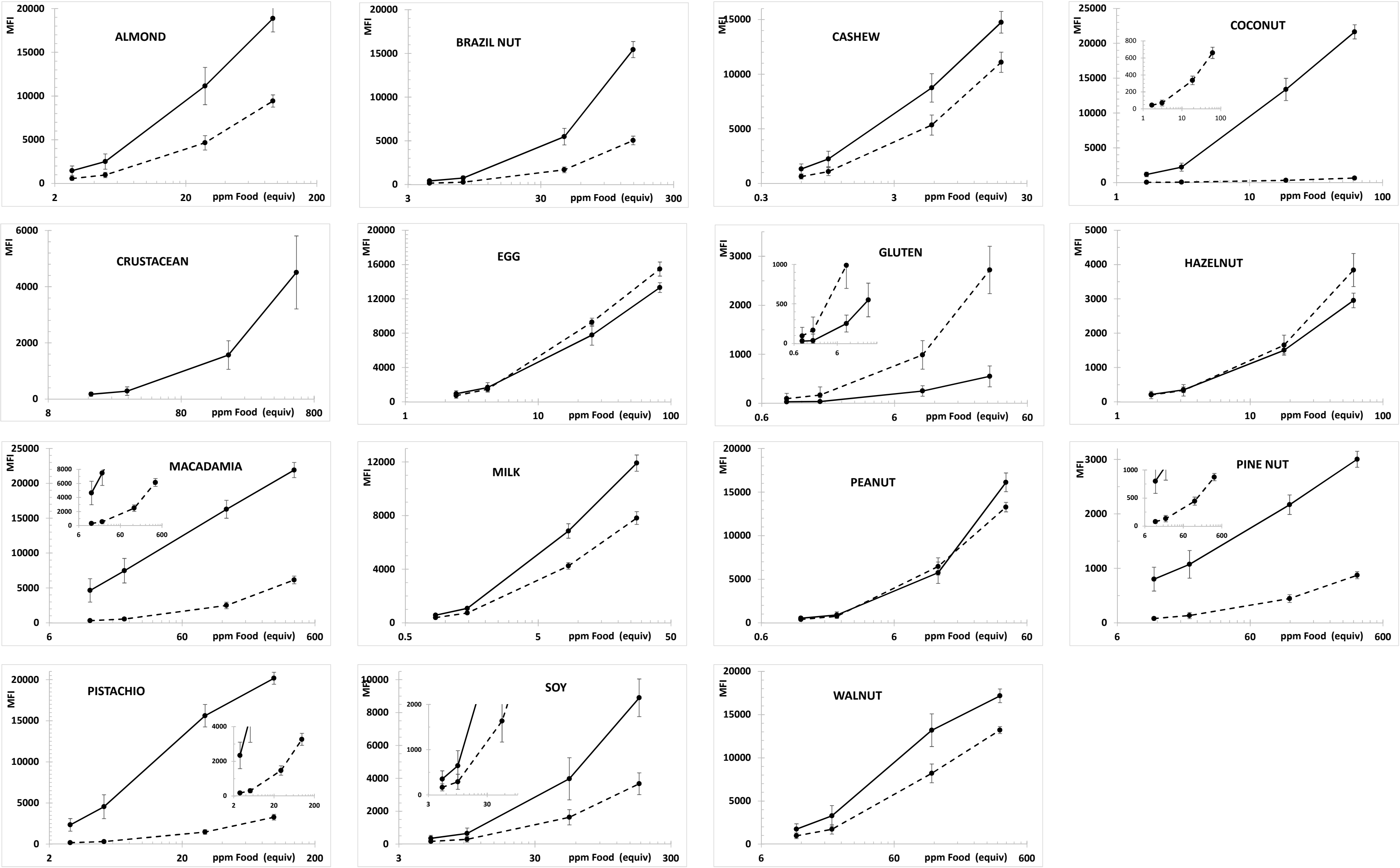
ALMOND
BRAZIL NUT
CASHEW
COCONUT
CRUSTACEAN
EGG
GLUTEN
HAZELNUT
MACADAMIA
MILK
PEANUT
PINE NUT
PISTACHIO
SOY
WALNUT
MFI
ppm Food (equiv)

Figure K
