# Supplementary material for: Multi-laboratory validation of the xMAP—Food Allergen Detection Assay: A multiplex, antibody-based assay for the simultaneous detection of food allergens
Source: PLoS One. 2020 Jul 9;15(7):e0234899. doi: 10.1371/journal.pone.0234899 (PMC7347184; doi:10.1371/journal.pone.0234899)
Supplement: S2 Fig — (PDF) [file pone.0234899.s002.pdf]

S2 FIGURE

S2 Figure. Comparisons between the MFI generated by the incurred food samples and the 20 ppm gluten DCCs (G20). The MFI generated by the gluten bead sets in the analyses of meat (red), orange juice (blue), baked muffin (green), and dark chocolate (purple) food samples minus the MFI of the comparable bead sets in the analysis of the DCCs plotted as a function of incurred allergenic food concentration. A- Gluten bead set-27; B- Gluten bead set-28. Plotted separately are the results for each laboratory participating in the MLV (Labs 01-11). Lab 05 did not analyze meat samples. The DCCs were prepared in triplicate by spiking analyte-free food samples with 20 µg/g gluten (G20) and performing the extraction - preparation (including the optional 10-fold dilution with PBST, 5-fold dilution with UD buffer for the dark chocolate samples) alongside the incurred food samples.

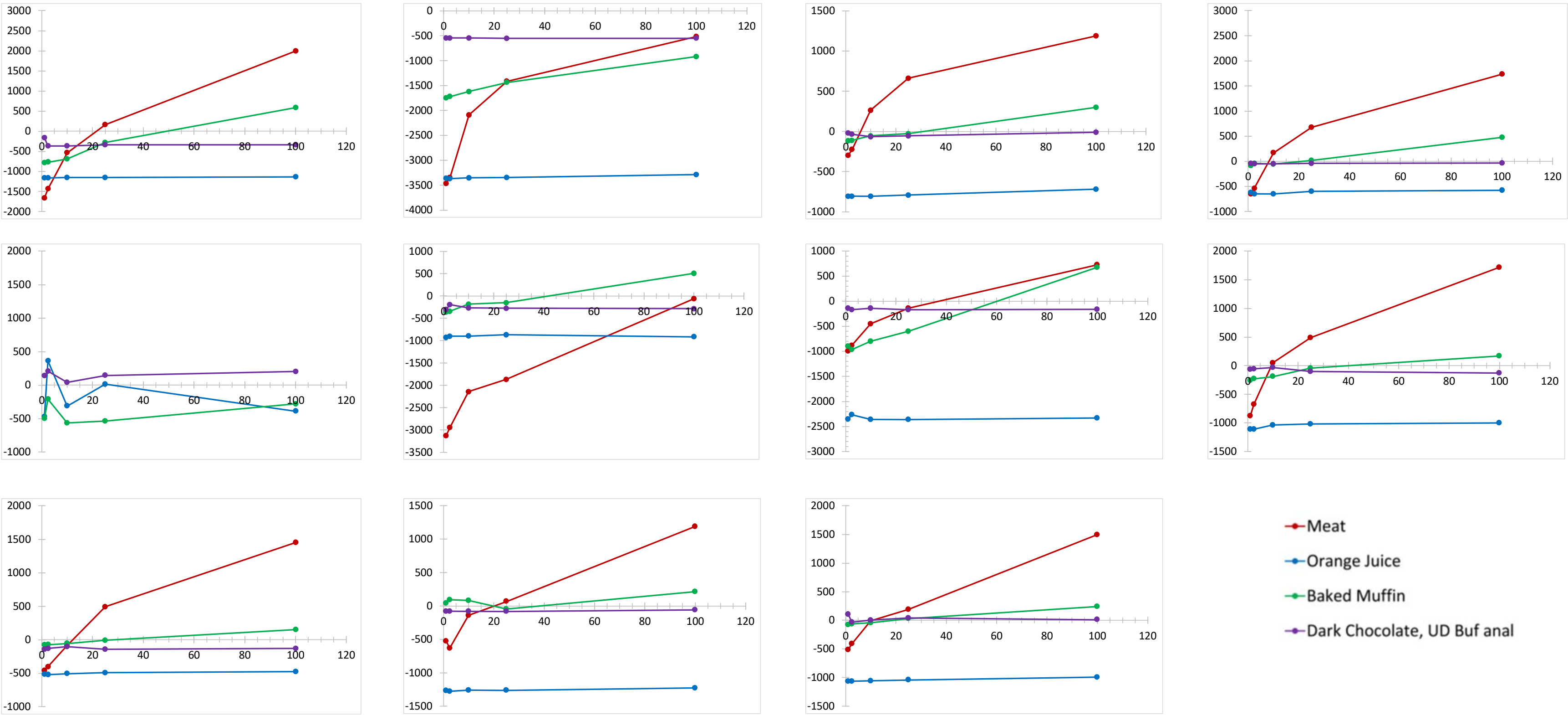

A

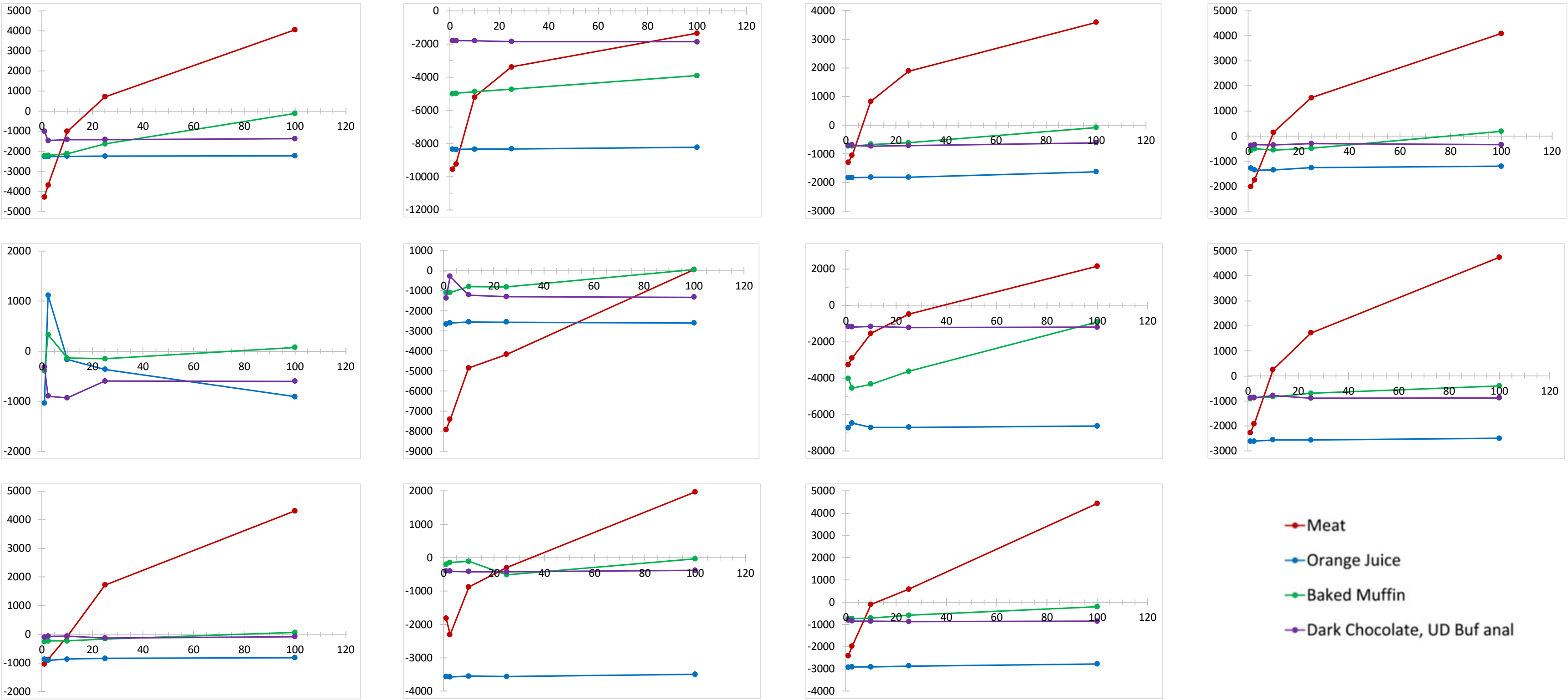

B
