# Supplementary material for: Multi-laboratory validation of the xMAP—Food Allergen Detection Assay: A multiplex, antibody-based assay for the simultaneous detection of food allergens
Source: PLoS One. 2020 Jul 9;15(7):e0234899. doi: 10.1371/journal.pone.0234899 (PMC7347184; doi:10.1371/journal.pone.0234899)
Supplement: S3 Fig — (PDF) [file pone.0234899.s003.pdf]

S3 FIGURE

S3 Figure. Comparisons between the MFI generated by the incurred food samples and the 50 ppm milk DCCs (M50). The MFI generated by the milk bead sets in the analyses of meat (red), orange juice (blue), baked muffin (green), and dark chocolate (purple) food samples minus the MFI of the comparable bead sets in the analysis of the DCCs plotted as a function of incurred allergenic food concentration. A- Milk bead set-35; B- Milk bead set-36. Plotted separately are the results for each laboratory participating in the MLV (Labs 01-11). Lab 05 did not analyze meat samples. The DCCs were prepared in triplicate by spiking analyte-free food samples with 50 µg/g milk (M50) and performing the extraction - preparation (including the optional 10-fold dilution with PBST) alongside the incurred food samples. Not included are Lab 09 (no milk data available) or data from the dark chocolate samples (analyzed using UD buffer).

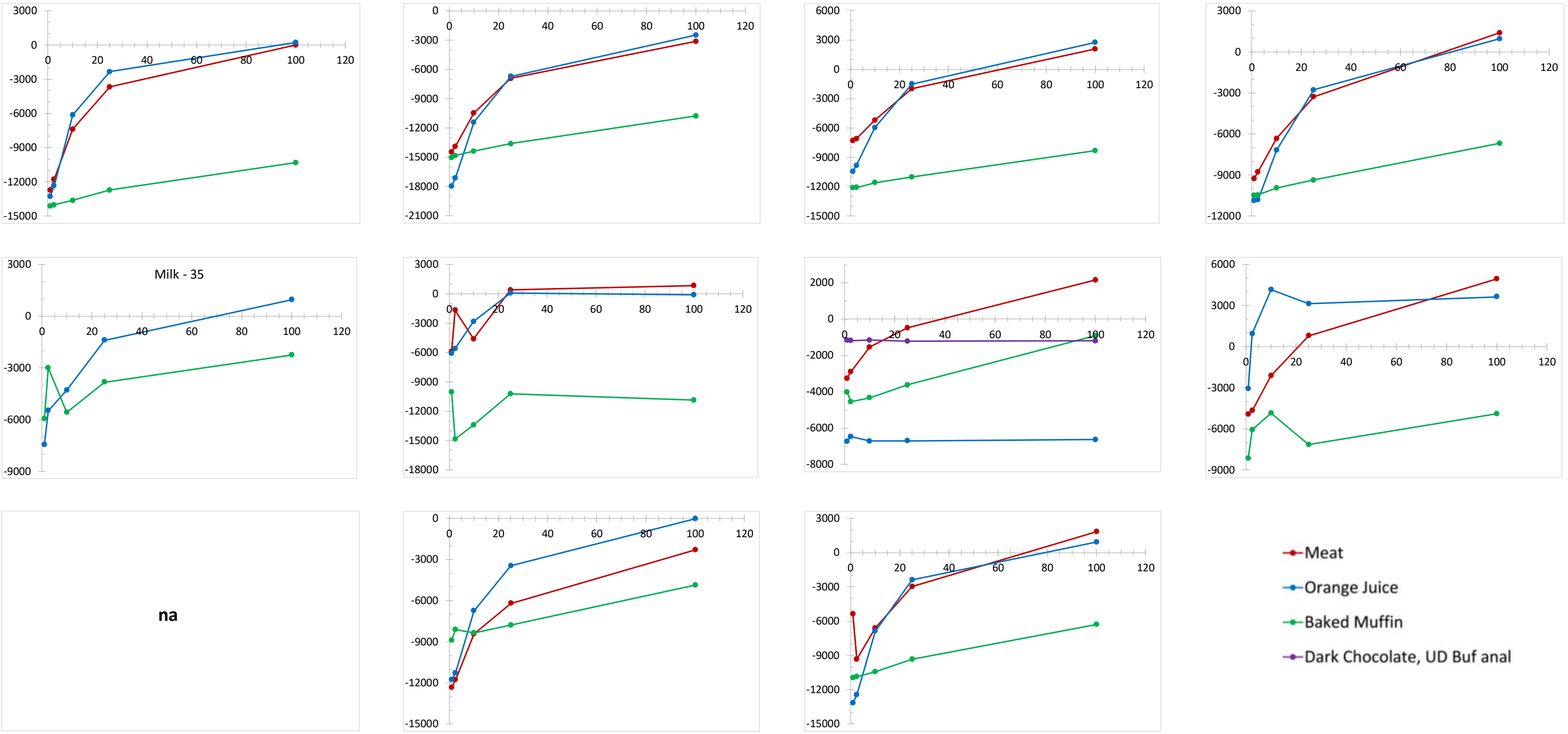
Milk - 35
na
Meat
Orange Juice
Baked Muffin
Dark Chocolate, UD Buf anal

A

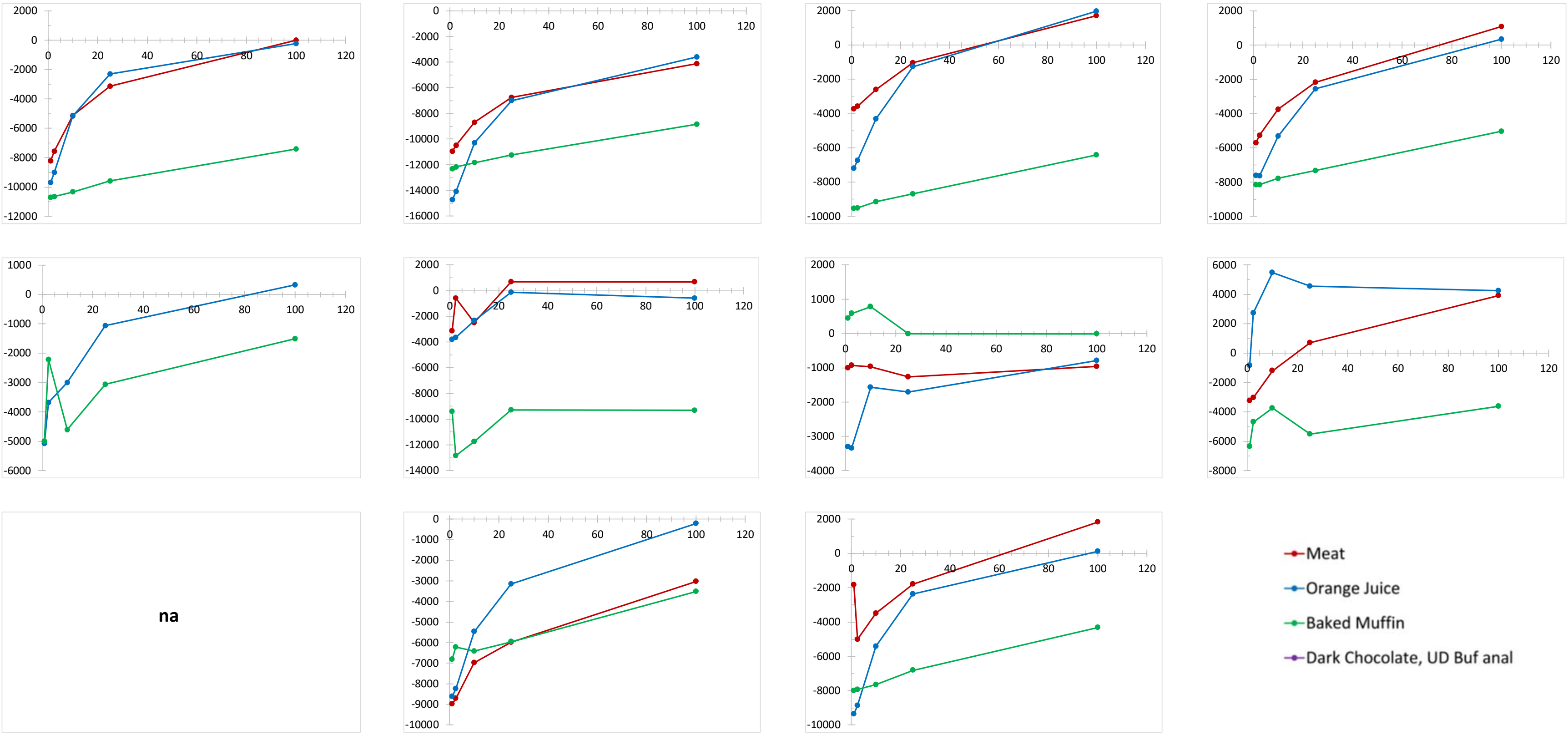
na
Meat
Orange Juice
Baked Muffin
Dark Chocolate, UD Buf anal

B
