# Supplementary material for: Multi-laboratory validation of the xMAP—Food Allergen Detection Assay: A multiplex, antibody-based assay for the simultaneous detection of food allergens
Source: PLoS One. 2020 Jul 9;15(7):e0234899. doi: 10.1371/journal.pone.0234899 (PMC7347184; doi:10.1371/journal.pone.0234899)
Supplement: S1 Appendix — (PDF) [file pone.0234899.s005.pdf]

S1 APPENDIX

S1 Appendix.
Instructional items supplied to the laboratories

xMAP FADA MULTI-LABORATORY VALIDATION

OVERVIEW

As described in the product insert, the xMAP Food Allergen Detection Assay (xMAP FADA) consists of two parts, an analysis based on a buffered-detergent extraction protocol (non-denatured, PBST or UD-Buffer based) and an analysis based on a reduced-denatured extraction protocol (denatured, SDS/β-mercaptoethanol based).

Each participating laboratory should receive initially 6 boxes, one for each of the four food sets and two for the xMAP FADA kits. A 7th box containing bead sets and detector antibodies for the reduced-denatured analyses will follow. The items shipped on dry ice (one of kit components, OJ, and sausage samples) should be immediately placed into a freezer (– 20 °C is acceptable if a – 80 °C is unavailable). The bead sets and other reagents of the xMAP FADA are supplied in vials, each containing the equivalent of 5 test kits.

Two sets of the food samples are included in each box. One set for analysis using the buffered-detergent extraction protocol and one set using the reduced denatured protocol. In addition, 9 analyte-free samples are included for preparation of 3 Direct Comparison Controls (DCC; G20, P10, M50) for each part plus 3 extra. Thus, each box should contain 6 of each coded sample (total of 36) plus 9 analyte-free samples, total of 45 food samples. The food samples are supplied pre-weighed (muffins weighed prior to baking).

Also included in the shipment are gluten, peanut, and nonfat milk powder reference materials for preparation of the Direct Comparison Controls (see attached instruction sheet).

The MLV is conducted in two phases, each given 4* weeks for completion.

**If more time is needed, please contact us. We greatly appreciate your kindly donating your time and wish to accommodate any needs.*

- In the first phase, the buffered-detergent analyses with the results due within 1 month
- In the second phase, the reduced-denatured analyses with the results due within 2 months

It is recommended that the analyses of each of the four different foods be performed on separate days.

To facilitate data processing, the samples should be analyzed according to the template below:

	1	2	3	4	5	6	7	8	9	10	11	12
**A**	S0		S0		S0		M19i		M19ii		M19iii	
**B**	S1		S1		S1		M28i		M28ii		M28iii	
**C**	S2		S2		S2		M37i		M37ii		M37iii	
**D**	S5		S5		S5		M46i		M46ii		M46iii	
**E**	S7		S7		S7		M55i		M55ii		M55iii	
**F**	G20		G20		G20		M64i		M64ii		M64iii	
**G**	P10		P10		P10							
**H**	M50		M50		M50							

I, ii, & iii refer to the three replicates, each prepared using a different sample tube (supplied).

The samples are placed in alternating columns to minimize cross contamination.

Two round bottom plates, one for each phase, are supplied per kit (10 total) for manual washing. If using an automatic magnetic bead plate washer, please contact us if clear flat-bottom plates are needed.

The MFI measurements and bead counts should be submitted as exported EXCEL spreadsheets.

COMMENTS & MODIFICATIONS

- Frozen samples (OJ & sausage) should be thawed to room temperature before adding extraction buffer

- Remove muffins from paper, then dice, and put back into the tubes for mixing with extraction buffer

- Chocolates analyzed using buffered-detergent must follow the UD Buffer option as modified below

  Normally chocolate samples are diced, but due to the incurring procedure dicing is not possible. Instead, dissolution is achieved using pre-heated buffer. Thus,

  -<u>For the buffered-detergent analyses of chocolate, the UD Buffer must be preheated to 60 °C</u>

  -<u>For the reduced-denatured analyses of chocolate, the PBST must be preheated to 60 °C</u>

- DCC are prepared per the attached instructions and extracted alongside the comparable coded food.

- As such, 21 extractions (3 of each of the 6 coded samples, plus the 3 DCC (one of each G20, P10, & M50) are required for each food in each phase of the assay.

- After performing the buffered-detergent extraction and centrifugation, the centrifugate (coded & DCC) <u>should be further diluted 10-fold with PBST or for the chocolate samples diluted 5-fold with UD Buffer</u>.

- PBST refers to PBS/0.1% Tween-20 (e.g., 1L PBS Sigma cat # P3813 + 1 mL Tween-20). Alternatively, Sigma cat # P3563 can be used. If Sigma P3563 PBST is used to make UD buffer (1:1 mixture with 200 mM NaPi + 2.5 g NFDM per 100 mL) then add 25 µL Tween-20 per 100 mL (0.25 mL per liter) to achieve 0.05%.

- Initially vortex the bead set mixture (15-20 sec.) to re-suspend, and briefly (1-2 sec) before pipetting. Use the appropriate mixture for the analysis being performed.

- The S0 calibration standards for the reduced-denatured analyses require that the extraction buffer (1:1 mixture of PBST & SDS/β-mercaptoethanol) be diluted the same 100X with PBST as the samples; analogous to SDS/β-mercaptoethanol diluted 200-fold with PBST.

- When a phase is completed, please e-mail the EXCEL data files with the workbooks appropriately titled to identify the food series being analyzed and the name of the analyst / analytical lab.

- Please feel free to contact us with any questions.

THANK YOU FOR MAKING THIS VALIDATION POSSIBLE.

PREPARATION OF DIRECT-COMPARISON CONTROLS (DCC– CMS)

Single tube of each DCC-CMS is prepared and loaded 3 times on the assay plate per template.

Often being able to make direct comparisons directly between a measured response generated by a food sample with a control of the same food matrix spiked with the target analyte at a defined critical concentration is useful. Other than gluten, the FDA is currently evaluating target analyte content on a case-by-case basis. For this MLV, Direct Comparison Controls (DCC) of gluten at 20 ppm (G20), peanut at 10 ppm (P10), and milk at 50 ppm (M50) are used. The DCC are prepared alongside the food samples, undergoing identical extraction and dilutions. The only difference is that the analyte of interest has been spiked into a comparable food at a known concentration. This then supports the statement '*The sample generated a response comparable/exceeding the response generated by X ppm in the presence of the food matrix*', it does not address any effects of food processing. To accommodate the inclusion of these DCCs, the calibration standards S3, S4, and S6 are omitted from the analyses (see template).

DCC- prepared on the day of use from either frozen stock solutions or powdered reference material.
The DCC-CMS are extracted and analyzed alongside the coded samples of the same food matrix.
Wheat gluten solutions must be used the day of preparation and cannot be stored frozen.
Transfers should be made using filtered, wide-bore pipette tips immediately after mixing.

Included in the shipment of samples should be
Bag with three vials of reference materials (non-fat dried milk, peanut, wheat gluten powder)
Bag with 9 tubes of pre-aliquoted, analyte-free food samples for each of the four foods
-3 tubes are used for each of the 2 extraction protocols with 3 tubes extra
-each tube contains 1 g (e.g., muffin mass prior to baking) of pre-aliquoted sample
-muffins must be removed from any attached paper, diced, and returned to vial for use

Gluten-20 (G20) : 20 ppm DCC-CMS
Prepare wheat gluten (provided) at 1 mg/mL (1,000 ppm) suspended in PBST on day of use,
powder extensively vortexed with PBST & let sit 2 hr at room temperature with periodic mixing
Spike 20 µL of 1,000 ppm gluten into one tube of analyte-free food, let sit at least 15 min
Prepare per protocol (extraction, centrifugation, & dilution), then load 50 µL into 3 wells per template.

Peanut-10 (P10): 10 ppm DCC-CMS
Prepare peanut reference material (provided) as a 1 % w/v (10,000 ppm) suspension in PBST
Divide into 100 µL aliquots for storage at -20 °C until needed.
Immediately before use, dilute 10,000 ppm peanut 50-fold with PBST (net 200 ppm)
Spike 50 µL 200 ppm peanut into an analyte-free food; let sit at least 15 min.
Prepare per protocol (extraction, centrifugation, & dilution), then load 50 µL into 3 well per template.

Milk-50 (M50): 50 ppm DCC-CMS
Prepare milk reference material (provided) at 10% w/v (100,000 ppm) in PBST
Divide into 100 µL aliquots for storage at -20 °C until needed.
Immediately before use, dilute 100,000 ppm milk 50-fold with PBST (net 2,000 ppm)
Spike 25 µL 2,000 ppm milk into an analyte-free food, let sit for at least 15 min.
Prepare per protocol (extraction, centrifugation, & dilution), then load 50 µL into 3 wells per template.

xMAP Food Allergen Detection Assay

Non-Denatured (Buffered-Detergent) PBST & UD-Buffer Protocols

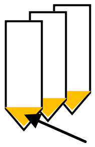

1. Pre-weighed coded food samples (in triplicate) and Direct Comparison Controls (prepared per instructions) in 50 mL conical tubes. Muffins should be removed from any paper and diced.

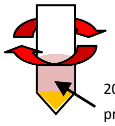

2. To each tube add either 20 mL PBST or for the <u>chocolate samples add 40 mL of UD Buffer pre-heated to 60 °C</u>. Vortex extensively to maximize dissolution.

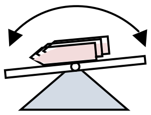

3. Rock (mix) for 2 hrs at room temperature.

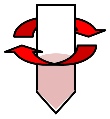

4. Vortex.

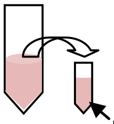

5. Transfer approx. 1 mL to a microfuge tube (e.g., 1.5 mL into a 2 mL microfuge tube).

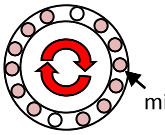

6. Centrifuge for 5 min at 14,000 x *rcf*.

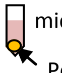

7. Dilute PBST samples 10X (e.g., 300 µL + 2.7 mL PBST). Dilute <u>UD Buffer samples 5X</u> (e.g., 300 µL + 1.2 mL UD Buffer).

Though <u>not necessary</u> for the current Multi-Laboratory Validation, any additional dilutions would be performed using either PBST or UD Buffer.

Denatured (Reduced-Denatured) Protocol

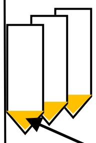

1. Pre-weighed coded food sqmples (in triplicate) and Direct Comparison Controls (prepared per instructions) in 50 mL conical tubes. Muffins should be removed from any paper and diced.

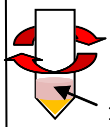

2. For each tube, add 10 mL PBST buffer or for the <u>chocolate samples add 10 mL of PBST pre-heated to 60 °C</u>. Vortex extensively to maximize dissolution.

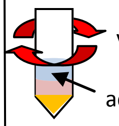

3. Add 10 mL SDS/β-mercaptoethanol and vortex.

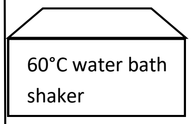

4. Place tubes in a 60°C water bath shaker for 30 min at 150 rpm.

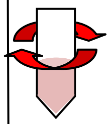

5. Vortex immediately after removing from water bath

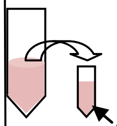

6. Transfer approx. 1 mL to a microfuge tube (e.g., 1.5 mL into a 2 mL microfuge tube).

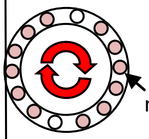

7. Centrifuge for 5 min at 14,000 x *rcf*.

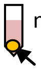

8. Dilute samples 100X (e.g., 100 µL + 9.9 mL PBST).

Though <u>not necessary</u> for the current Multi-Laboratory Validation, any additional dilutions would be performed using Diluent (SDS/β-mercaptoethanol diluted 200X with PBST).

Preparation of Calibration Standards

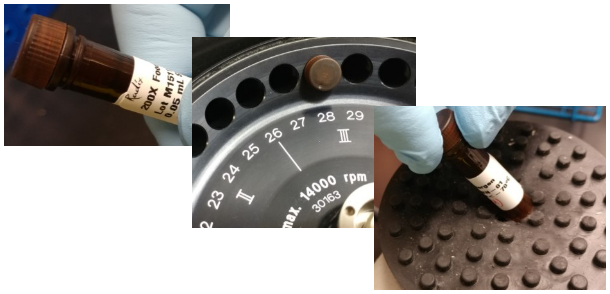

1. Remove and fully thaw a tube of 200X Reference Material - Calibrant Solution to prepare tet kit calibration standards (S1 - S7). This is different from the material supplied to prepare the Direct Comparison Controls.
2. Centrifuge the tube briefly (1-2 sec).
3. Vortex (1-2 sec) before removing an alliquot for subsequent dilution.

Non-Denatured (Buffered-Detergent) Assay

4. Pipette 5 µL of well mixed 200X calibrant solution and transfer to STD7 (S7) tube containing 995 µL PBST (or UD Buffer for the choclate samples).

5. Continue with serial dilutions as shown in Table 1

.

Table 1. Non-Denatured Calibration Standards

Calibrator Tube	Dilution	µL of Reference Material or Source Tube	µL of PBST (or UD Buffer)
**STD 7**	**200X**	5	995
**STD 6**	360	500 of STD 7	400
**STD 5**	648	500 of STD 6	400
**STD 4**	1166	500 of STD 5	400
**STD 3**	3000	500 of STD 4	400
**STD 2**	3779	500 of STD 3	400
**STD 1**	6802	500 of STD 2	400
**STD 0**	1	0	400

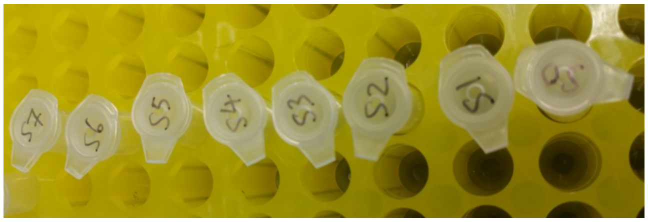

Denatured (Reduced-Denatured) Assay

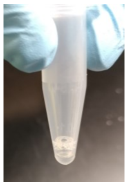

4. Pipette 10 µL of well mixed 200X calibrant solution into an Eppendorf Tube. Pipette 10 µL of β-mercaptoethanol (BME)/SDS Extraction buffer into the same tube and mix well.

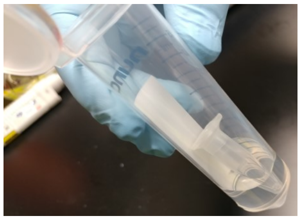

5. Place approx. 10 mL distilled water into a 50 mL conical tube. Place the tube from Step 4 into the 50 mL tube and cap.

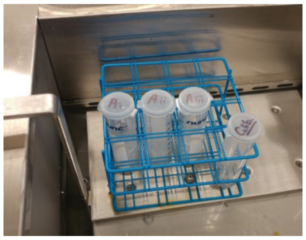

6. Incubate the tube assembly from step 5 in a 60 C water bath shaker for 30 min.

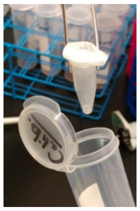

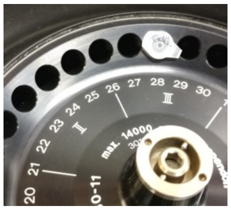

7. Centrifuge briefly (1-2 sec) to bring down condensate. Then mix well.

8. Transfer 10 µL into an tube containing 990 µL of PBST for the 100-fold dilution to generate STD7 (S7). Serially dilute with Diluent (200-fold dilution of SDS/β-mercaptoethanol with PBST) according to Table 2, below.

Table 2. Denatured Calibration Standards

Calibrator Tube	Dilution	µL of Reference Material or Source Tube	µL of Buffer	
**STD 7**	**100X**	10	990	**PBST**
**STD 6**	360	500 of STD 7	400	Diluent
**STD 5**	648	500 of STD 6	400	Diluent
**STD 4**	1166	500 of STD 5	400	Diluent
**STD 3**	3000	500 of STD 4	400	Diluent
**STD 2**	3779	500 of STD 3	400	Diluent
**STD 1**	6802	500 of STD 2	400	Diluent
**STD 0**	1	0	400	Diluent

Manual Washing

Materials needed:

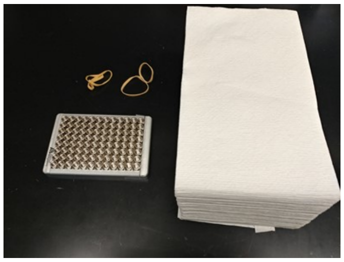

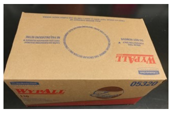

Magnetic-Ring Stand (96 well) (Ambion, Cat#AM10050)

Rubber bands, size 33 (3/12” x 1/8 “) or similar

WYPALL (L10) or similar paper towels

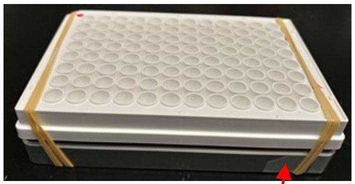

Plate—Magnetic-Ring Stand assembly

Clear tape to cover the gaps on the stand

1. Remove the plate seal
2. Assemble the assay plate on top of the Magnetic-Ring stand
3. Secure with rubber bands at both ends
4. **Wait at least 2 mins** to allow the beads to be pulled down to the bottom of the plate.
5. Hold firmly onto both plate and magnetic stand
6. Remove liquid by flipping the assembly up-side down with a downward motion in the sink.
7. **Still inverted,** tap gently the plate assembly on paper toweling (e.g., WYPALL L10) to remove excess liquid. Repeat several times on clean areas of the toweling.

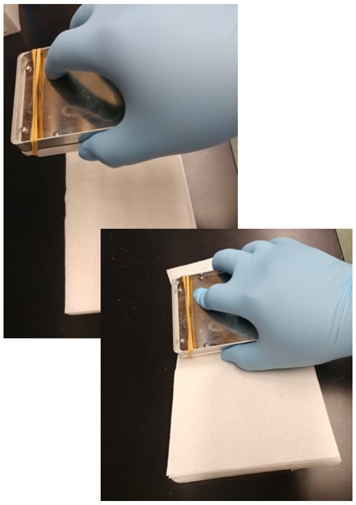

Gentle tapping is sufficient to remove excess liquid. Use stack of paper towels.

First Wash:

8. Place assembly right-side up.
9. Add 200 µL of wash buffer (PBST) per well using a multi-channel pipette.
10. **Wait at least 2 mins** to allow the beads to pulled down to the bottom of the plate.
11. Remove liquid by flipping the assembly up-side down with a downward motion in the sink.
12. **Still inverted**, tap gently the plate assembly on paper toweling (e.g., WYPALL L10) to remove excess liquid. Repeat several times on clean areas of the toweling.

Second and Third Washes:

13. Repeat above steps 8 to 12 for the second and third washes.
    Make sure to wait at least 2 mins to allow the beads to settle to the bottom of the plate.
14. After the third wash, proceed to the next step in the assay protocol.

Food Allergen Detection Assay Protocol 5 – Pack (48 Well)

For the rapid detection of Crustacean, Egg, Milk, Peanut, Soy, Tree Nuts (Almond, Brazil Nut, Cashew, Coconut, Hazelnut, Macadamia, Pine Nut, Pistachio, Walnut) and Gluten

www.radixbiosolutions.com
512.869.8000

Food Allergen -Kit Contents:

2-8°C Storage:

1) 250X Bead sets for the non-Denatured Assay	22 µL/kit
2) 250X Bead sets for the Denatured Assay (shipped separately)	22 µL/kit
3) 100X AssayCheX	105 µL/kit
4) 250X Detection Antibodies for non-Denatured Assay	22 µL/kit
5) 250X Detection Antibodies for Denatured Assay (shipped separately)	22 µL/kit
6) 333X SAPE Reporter	20 µL/kit
7) 96-well Plate (suitable for some plate washers)	1 per kit

-20°C (-80°C preferred) Storage:

8) 200X Reference Material	90 µL/kit

A two-kit package would contain 44 µL of each of the bead set cocktails, 210 µL of the AssayChex™, 44 µL of each of the detection mix antibody mixes, 40 µL of the SAPE reporter, two microtiter plates, and 20 µL of the reference material. Similarly, a five-kit package would contain 110 µL of each of the bead set cocktails, 525 µL of the AssayChex™, 110 µL of each of the detection mix antibody mixes, 100 µL of the SAPE reporter, five microtiter plates, and 50 µL of the reference material.

TABLE OF CONTENTS

Contents	Page
Introduction	4
Principle of the Assay	4
Reagent Components	5
Other Supplies Required	6
Storage	7
Buffers	7
Technical Notes	7
Instrumental Setup	8
Reagent Preparation	8
Preparation of Calibrators	8
Extraction and Preparation of Samples	10
Preparation of Bead Cocktail Mixes	11
Preparation of Detection Antibody Mixes	12
Assay Procedure	14
Data Analysis	15
References	16
Sample Notes	17
Plate Layouts	18

MANUFACTURED AND DISTRIBUTED BY:

Radix BioSolutions Ltd.
111 W Cooperative Way, Suite 120
Georgetown, TX, USA 78626
Phone: 512.869.8000
Fax: 512.868.9040
Email: info@radixbiosolutions.com

INTRODUCTION

The xMAP® Food Allergen Detection Assay (xMAP® FADA) kit is intended for the rapid detection of seven of the Big Eight food allergens that account for 90% of food allergies plus gluten, which causes Celiac sprue. The kit detects 14 food allergens (crustacean, egg, milk, peanut, soy, wheat, and the tree nuts almond, Brazil nut, cashew, coconut, hazelnut, macadamia, pine nut, pistachio, and walnut) plus gluten. Using two extraction protocols, buffered-detergent and reducing-denaturing conditions, and two bead sets for all analytes, except crustacean and egg, the kit provides built-in confirmation.

PRINCIPLE OF THE ASSAY

The xMAP® Food Allergen Detection Assay (xMAP® FADA) employs antibodies already established in commercial ELISA test kits for their high specificity and reliability. The antibodies are covalently coupled to different color-coded microspheres (beads). The assay contains a total of 30 individual antibody-conjugated beads for the detection of 15 different analytes. The assay was developed with redundancy, where possible. Two capture/detector antibodies pairs are present for all allergens with the exception of crustacean and egg. AssayChex™ Process Controls are included to assure that all assay reagents are performing optimally.

xMAP® technology offers the benefits of liquid reaction kinetics and through the ability to magnetically remove the microspheres, the advantages of surface chemistry. The food allergens and gluten are extracted using the two extraction protocols routinely employed in ELISA test kits, dilution into buffered-detergent (non-Denatured) and SDS/β-mercaptoethanol (Denatured). The extracted samples are mixed with a cocktail of the antibody-conjugated bead sets where the antibodies bind to their respective target analytes, if present. The mixture is washed to remove unbound sample and a biotinylated secondary detection antibody is added that binds the complex. Use of target-specific detection antibodies provide the same high-specificity associated with sandwich ELISAs. Addition of reporter spreptavidin phycoerythrin (SAPE) generates a signal that is detected by a Luminex instrument (e.g., MagPix®, Luminex 100/200TM, Bio-Plex®). Reference material containing all targeted allergens is provided to prepare calibration curves for quantitation

and to facilitate comparison between plates and different lots of test kits.

Each kit contains enough reagent to analyze 48 samples, controls, and calibrants using the buffered-detergent and reduced-denatured extraction protocols. Though all analyses can be performed using a single microtiter plate, it is recommended that the two extraction protocols be performed separately and that alternating columns of the microtiter plate be used to minimize cross-contact. A second microtiter plate suitable for use with some plate washers is not supplied but can be purchased from Greiner Bio-One, Cat#655090-96-well clear bottom flat plate.

REAGENT COMPONENTS

250X Antibody Coupled Beads– antibody conjugated magnetic microspheres in a buffered solution

250X Detection Antibody – biotin-conjugated antibodies in a buffered solution

100X AssayCheX - AssayCheX™ process control microspheres in a buffered solution

333X Reporter – streptavidin phycoerythrin (1 mg/mL) in a buffered solution

200X Reference Material-Calibrant Solution – A mixture of all 14 allergens plus gluten prepared in buffered solution. When serially diluted 1:1.8 calibration curves may be prepared for all allergens with a range spanning the lower limit of detection (LLoD) to the upper limit of detection (ULoD); see table under Preparation of Calibrators.

Besides providing calibration curves, the reference material-calibrant solution facilitates comparison between results generated using different plates, test kits from different lots, and data generated by different analysts. A slight distortion in the calculated amount of an analyte is possible due to cross-reactivity (Anal Bioanal Chem 2015, 407:4195-206) and food processing. To improve the accuracy of allergen or gluten quantitation, it is recommended that incurred reference materials spanning the analytical range of interest be prepared using the same processing

as the samples and analyzed alongside the samples and the test kit calibrants.

Notes- The diluted calibrant solutions can be stored at 4 ^{0}C for one week. Multiple refreezing of the diluted calibrants in buffered-detergent is not recommended.

A denaturing treatment is required for the denatured assay (e.g., 1:1 mixture with SDS/β-mercaptoethanol solution, 30 min 60 ºC, 100-fold dilution with PBST followed by 1:1.8 serial dilution with diluent using LoBinding tubes, see sample preparation).

ADDITIONAL REAGENTS REQUIRED

PBS (Sigma-Aldrich, Cat#P3813-10PAK)
PBST 1 L PBS + 1 mL Tween-20 or Sigma-Aldrich Cat# P3563
Beta-mercaptoethanol (Sigma-Aldrich, Cat#M6250 or similar)
Sodium Dodecyl Sulfate (Sigma-Aldrich, Cat#L6026 or similar)
Tween-20 (Sigma-Aldrich Cat#P9416)
Sodium Phosphate, Monobasic Monohydrate (FW 137.99)
Sodium Phosphate, Dibasic (FW 141.96)
Skim Milk Powder (BD Cat#232100 or Fisher Cat# DF0032-17-3)

ADDITIONAL EQUIPMENT REQUIRED

xMAP system (e.g., MagPix, Bio-Plex, or Luminex 100/200)
Magnetic Plate (e.g., Ambion Cat#AM10050) or plate washer
Microtiter Plate Shaking Incubator (e.g., EchoTherm™ SC25 Orbital w/round & flat bottom SmartBlocks for 96-well plates, & lid)
Shaking/Rocking Incubator for Non-Denatured (ambient temp.) and Denatured (60 ºC) extraction of samples.
96-well round bottom plates for capture, detector, & label binding incubation steps and is compatible with Magnetic Plate (e.g., Corning Costar 3789 or low binding polypropylene plates).
Microcentrifuge tubes for preparation of serially diluted calibrants (e.g., USA Scientific CAT#1415-250 or Eppendorf LoBind Cat#022431081).
Various pipettes / pipettors – recommend filtered tips and wide-bore tips for pipetting samples and bead sets.
Centrifuge or microfuge for centrifuging food extracts at 14,000xg.
Disposable nitrile gloves
Vortex mixer

STORAGE

Store kits (bead sets) in the dark at 2-8°C.
Store reference materials at -20°C (-80°C preferred).
Store sample extracts at -20°C.

BUFFERS

Wash Buffer – PBST
Assay Buffers – *depends on the assay*
Non-Denature (buffered-detergent extract analysis)
PBST– *general use extraction, dilution and assay buffer*
UD Buffer– *alternative to PBST, recommended for pure plant products or when more stringent binding conditions preferred*. Prepared as a 1:1 mixture of PBST with 200 mM sodium phosphate*, non-fat dried milk is added to 2.5%. If using Sigma Cat# P3563 PBST, add 25 µL Tween-20 per 100 mL to achieve 0.05% Tween-20. *prepared as 1:1 molar ratio of monobasic : dibasic phosphate.

Denature (reduced-denatured extract analysis)
Extraction solution- 0.5% SDS/2% β-mercaptoethanol in PBS
Diluent- *for serial dilution of calibrants and additional dilution of samples exceeding the upper limit of the assay*. Extraction buffer diluted 1:200 with PBST

TECHNICAL NOTES

- Use good laboratory practices (e.g., change pipette tips between additions and using unique reservoirs for reagents).
- Avoid foaming of solutions by mixing gently.
- Protect the Bead Mix and Reporter from light.
- Protect the plate from light once reagents are added.
- Do not mix lots or substitute reagents in the assay.
- Assay Buffer (PBST, UD Buffer, or Diluent) may be used as negative controls.
- Always run the instrument wash command between runs.
- AssayCheX™ internal controls are included in this assay, which includes an instrument control, an antibody control, a fluorescent reporter control, and a negative control.
- Be sure all incubators are allowed to equilibrate to temperature (e.g., 37 ºC and 60 ºC) prior to use.
- Lot-Lot variability may be observed.

INSTRUMENTAL SETUP:

1. Perform start-up and calibration procedures according to the instrument manuals and laboratory policies.
2. Run the wash command immediately prior to analysis.
3. Use the default instrument settings for MagPix analysis.
4. Use the following settings for Bio-Plex or Luminex 100/200
   a. Doublet Discriminator gate – (5,000- 25,000)
   b. PMT setting – Default (Low)
   c. Number of microspheres collected – 100 per region
   d. Specify MagPlex beads for monitoring

REAGENT PREPARATION:

Prepare enough of each reagent for one day.
Thaw a new vial of calibrator daily.
Discard any remaining calibrator, diluted calibrators and leftover diluted bead mix, diluted detection antibody and diluted SAPE reagents at the end of each day.
UD before should be prepared fresh daily.
Filter sterilized PBS, PBST, and sodium phosphate buffer are stable indefinitely with proper handling.

1. PREPARATION OF CALIBRATORS

Non-Denatured Assay: Reference Materials-Calibrant Solution is supplied as a 200-fold concentrate, which when diluted with PBST (e.g., 5 µL + 995 µL PBST) generates the STD7 calibration solution with the 15 analytes at the following concentrations in ng/mL (upper limit of quantitation, ULOQ):

Reference Material	200X (ng/mL)	ULOQ (ng/mL)
Almond	19750	99
Brazil Nut	19750	99

Reference Material	200X (ng/mL)	ULOQ (ng/mL)
Macadamia Nut	32917	165
Milk	9880	49

Reference Material	200X (ng/mL)	ULOQ (ng/mL)
Cashew	3130	16
Coconut	4339	22
Crustacean	105000	525
Egg	39500	198
Gluten	25000	125
Hazelnut	7900	40

Reference Material	200X (ng/mL)	ULOQ (ng/mL)
Peanut	10500	52.5
Pine Nut	54861	274
Pistachio	19750	99
Soy	62500	313
Walnut	62500	313

Additional, 1.8-fold serial dilution of STD7 generates STD6 – STD1. The blank (STD0) is prepared using PBST.

Calibrator	Dilution	µL of Reference Material	µL of PBST
STD 7	200X	5	995
STD 6	360	500 of STD 7	400
STD 5	648	500 of STD 6	400
STD 4	1166	500 of STD 5	400
STD 3	3000	500 of STD 4	400
STD 2	3779	500 of STD 3	400
STD 1	6802	500 of STD 2	400
BLANK	1	0	400

The resulting calibrator concentrations are (ng/mL):

Reference Material	STD 7 (ULOQ)	STD 6	STD 5	STD 4	STD 3	STD 2	STD 1 (LLOQ)
Almond	99	55	30	17	9.4	5.2	2.9
Brazil Nut	99	55	30	17	9.4	5.2	2.9
Cashew	16	8.7	4.8	2.7	1.5	0.8	0.5
Coconut	22	12	6.7	3.7	2.1	1.1	0.6
Crustacean	525	292	162	90	50	28	15
Egg	198	110	61	34	19	10	5.8
Gluten	125	69	39	21	12	6.6	3.7
Hazelnut	40	22	12	7	3.8	2.1	1.2
Macadamia Nut	165	91	51	28	16	8.7	4.8
Milk	49	27	15	8	4.7	2.6	1.5
Peanut	52.5	29.2	16.2	9	5	2.8	1.5

Reference Material	STD 7 (ULOQ)	STD 6	STD 5	STD 4	STD 3	STD 2	STD 1 (LLOQ)
Pine Nut	274	152	85	47	26	15	8.1
Pistachio	99	55	30	17	9.4	5.2	2.9
Soy	313	174	96	54	30	17	9.2
Walnut	313	174	96	54	30	17	9.2

More stringent binding conditions, if required, replace PBST with UD buffer (recommended for pure plant products).

Denatured Assay: Mix the 200X Reference Materials-Calibrant Solution 1:1 with extraction buffer (e.g., 10 µL + 10 µL SDS/β-mercaptoethanol in PBS, see Buffers), incubate 30 min at 60 ºC, then dilute 100-fold with PBST (e.g., 10 µL + 990 µL PBST) to generate STD7. Serially dilute 1.8-fold with Diluent (see Buffers) to generate STD6–STD1. Use Diluent for the blank (STD0). Serial dilutions should be performed using the low binding Eppendorf tubes listed in Additional Equipment Required.

2. EXTRACTION AND PREPARATION OF SAMPLES

Non-Denatured Assay:

Food samples are mixed 1 g with 20 mL PBST for two hours with shaking at room temperature, then centrifuged at 14,000xg prior to analysis.

For more stringent binding conditions (e.g., for pure plant products), 1 g is mixed with 40 mL UD buffer for two hours with shaking at room temperature, then centrifuged at 14,000xg prior to analysis.

If expect the sample is above the ULOQ, the sample should be diluted with either PBST or UD, as appropriate.

Denatured Assay:

Food samples are mixed 1 g with 10 mL PBST, then mixed 1:1 with 10 mL 0.5% SD/2% β-mercaptoethanol in PBS (extraction buffer) for 30 min at 60 ºC with shaking (150 rpm), centrifuged at 14,000xg, then diluted 100-fold with PBST.

If expect the sample is above the ULOQ, the sample should be diluted with Diluent (see Buffers), as appropriate.

To simplify sample handling, use the plate map provided at the end of the protocol to map the positions for calibratnts and samples. It is recommended that the Non-Denatured and Denatured

Assays be ran on separate plates with alternating columns left empty; this requires a second microtiter plate.

3. PREPARATION OF BEAD COCKTAIL MIXES

Beads sets are provided as 250X concentrates. Beads should be diluted immediately before use to a 1X concentrate in PBST.

Vortex well before dilution to ensure a homogenous mixture.

Prepare beads for Non-Denatured & Denatured assays separately.

1- Calculate the required volume of bead mix needed. 50 µL is required for each well. It is recommended that 55 µL of bead mix be prepared for each well to account for potential loss during mixing and reagent addition.

Example: If analyzing for a single analyte in 16 wells, the following formula may be used to prepare the bead mix:
16 wells X 55 µL per well = 880 µL. Therefore, (880 µL) X (1X beads per mL) = (250X beads per mL) X (Y µL), or Y = 3.52 µL of concentrated beads added to 876.5 µL of PBST. When multiplexing, for each subsequent bead set, add 3.52 µL of beads and 3.52 µL less PBST.

If AssayCheX controls are being used, they are supplied at 100X. Calculate the required volume needed for the assay based upon the number of wells. Reduce the Assay Buffer diluent accordingly.

a. Non-Denatured Assay Bead Mix - Dilute beads 1:250 into PBST. The magnetic regions for the non-denatured system are:

Allergen	Bead ID
Almond	12
	13
Brazil Nut	14
	15
Cashew	18
	19
Coconut	20

Allergen	Bead ID
Egg	25
	26
Gluten	27
	28
Hazelnut	29
	30
Macadamia	33

Allergen	Bead ID
Peanut	37
	38
Pine Nut	39
	42
Pistachio	43
	44
Soy	45

Allergen	Bead ID
	21
**Crustacean**	22

Allergen	Bead ID
**Nut**	34
**Milk**	35
	36

Allergen	Bead ID
	46
**Walnut**	47
	48

b. Denatured Assay Bead Mix - Dilute beads 1:250 into PBST. The magnetic regions for the non-denatured system are:

Allergen	Bead ID
**Egg**	65
**Gluten**	73
**Milk**	66
	67
**Peanut**	72

AssayCheX™ Process control microspheres are included in the kit to assure that the system and reagents are performing optimally.

Bead 75	IC	Monitors the xMAP analyzer reporter fluorescence measurement
Bead 76	FC	Monitors the addition of the fluorescent reporter streptavidin phycoerythrin (SAPE) reagent to the well
Bead 77	AC	Monitors the addition of the biotin-labeled detection antibody reagent to the well
Bead 78	NC	Monitors the non-specific binding (if any) due to the sample matrix

4. PREPARATION OF DETECTION ANTIBODY MIXES

Detection antibodies provided individually as 250X Concentrates.

Detection antibodies should be diluted immediately before use to a 1X concentrate in PBST.

Prepare detection antibody mixes for Non-Denatured & Denatured assays separately.

There are two separate soy detection reagents. Use both reagents when detecting soy allergens.

1- Calculate the required volume of detection antibody needed. 50 µL is required for each well. It is recommended that 55 µL of bead mix be prepared for each well to account for potential loss during mixing and reagent addition.

Example Dilution: If analyzing for a single analyte in 16 wells, the following formula may be used to prepare the mix:
16 wells X 55 µL per well = 880 µL Therefore, (880 µL) X (1X detection antibody per mL) = (250X detection antibody per mL) X (Y µL), or Y = 3.52 µL of detection antibody added to 876.5 µL of PBST. When multiplexing, for each subsequent allergen, add 3.52 µL of the appropriate detection antibody and 3.52 µL less PBST.

a. Non-Denatured Assay Detection Antibodies - Dilute detection antibody 1:250 into PBST. The biotinylated antibodies for the non-denatured assay are:

Allergen	Description
Almond	goat anti-almond
Brazil	chicken anti-brazil nut
Cashew	chicken anti-cashew
Coconut	chicken anti-coconut
Crustacean	chicken anti-crustacean
Egg	goat anti-chicken ovomucoid
Gluten	chicken anti-gluten
Hazelnut	chicken anti-hazelnut

Allergen	Description
Macadamia Nut	chicken anti-macadamia nut
Milk	rabbit anti-bovine casein
Peanut	goat anti-peanut
Pine Nut	chicken anti-pine nut
Pistachio	chicken anti-pistachio
Soy (1)	goat anti-soy IgG
Soy (2)	anti-soy IgG
Walnut	goat anti-walnut

b. Denatured Assay Detection Antibodies - Dilute detection antibody mix 1:250 into Assay Buffer. The biotinylated antibodies for the denatured system are:

Allergen	Description
Egg	Polyclonal anti-(denatured) Egg
Milk	Polyclonal anti-(denatured) beta lactoglobulin
Milk	Polyclonal anti-(denatured) Casein
Gluten	Polyclonal anti-(denatured) Wheat
Peanut	Polyclonal anti-(denatured) Peanut

ASSAY PROCEDURE:

Note: Ensure the appropriate use of reagents for Non-Denatured and Denatured assays. It is recommended that this information is identified on the plate layout.

1. Add 50 µL of Calibrators/Samples to each well. See Pages 8 -10 for preparation of calibrants and samples.
2. Add 50 µL of bead mix to each well according to the plate layout.
3. Incubate for 30 minutes in the dark at 37°C, with shaking at 600 - 700 rpm.
4. At the end of the incubation, wash each well with 200 µL of PBST three times. Washes may be performed either by hand or with a magnetic plate washer.
5. Add 50 µL of detection antibody mix to each well according to the plate layout.
6. Incubate for 30 minutes in the dark at 37°C, with shaking at 600 - 700 rpm.
7. At the end of the incubation, wash each well with 200 µL of PBST three times. Washes may be performed either by hand or with a magnetic plate washer.

8. Add 50 µL of 1X Reporter (SAPE) to each well according to the plate layout.
9. Incubate for 15 minutes in the dark at 37°C, with shaking at 600 - 700 rpm.
10. At the end of the incubation, wash each well with 200 µL of PBST three times. Washes may be performed either by hand or with a magnetic plate washer.
11. Add 100 µL of PBST to each well. Analyze the assay on an instrument that is capable of reading MagPlex beads (e.g., MagPix, Bio-Plex, Luminex 100/200).
12. Each laboratory should follow the protocols in keeping with their QC/QA guidelines for certification and accreditation. This may require the inclusion of controls and additional replicates not necessarily described above.

DATA ANALYSIS

1. Calculate the mean MFI value for the negative control samples.
2. Determine the cut-off value by calculating 3 standard deviations above the mean negative control value.
3. Samples above the cut-off value may be positive. However, unless the sample exceeds the limit of quantition or STD1 it should be reported as '<LOQ' and either concentrated prior to retesting or analyzed using a more sensitive method.

Optimal curve fits for each analyte class were determined to be:

Allergen	Bead ID/<br>Curve Fit
**Almond**	12<br>5-PL
	13<br>5-PL
**Brazil Nut**	14<br>5-PL
	15<br>5-PL
**Cashew**	18<br>5-PL
	19<br>5-PL
**Coconut**	20<br>5-PL
	21<br>5-PL
**Crustacean**	22<br>5-PL

Allergen	Bead ID/<br>Curve Fit
**Egg**	25, 26<br>5-PL
	65<br>5-PL
**Gluten**	27, 28<br>5-PL
	73<br>5-PL
**Hazelnut**	29<br>5-PL
	30<br>5-PL
**Macadamia Nut**	33<br>5-PL
	34<br>5-PL
**Milk**	35, 36<br>5-PL
	66, 67<br>5-PL

Allergen	Bead ID/<br>Curve Fit
**Pine Nut**	39<br>5-PL
	42<br>5-PL
**Pistachio**	43<br>5-PL
	44<br>5-PL
**Peanut**	37, 38<br>5-PL
	72<br>5-PL
**Soy**	45<br>5-PL
	46<br>5-PL
**Walnut**	47<br>5-PL
	48<br>5-PL

5-PL = 5-Parameter Logistic

Though 5-Parameter Logistic plots generated excellent trendlines, it does not mean that other curve fitting equations may not generate comparable, or possibly superior, trendlines. It is the analyst's responsibility to ascertain the suitability of the approach used.

REFERENCE

Please refer to xMAP® User's Manual that corresponds to your instrument for further detail on use and maintenance of your xMAP® system.

Luminex® and xMAP® are registered trademarks of Luminex Corporation. Radix BioSolutions® and AssayCheX™ are trademarks of Radix BioSolutions, Ltd.

SAMPLE PREPARATION NOTES:

PLATE LAYOUT 1

	1	2	3	4	5	6	7	8	9	10	11	12
A												
B												
C												
D												
E												
F												
G												
H												

NOTES:

PLATE LAYOUT 2

	1	2	3	4	5	6	7	8	9	10	11	12
A												
B												
C												
D												
E												
F												
G												
H												

NOTES:

www.radixbiosolutions.com

111 W Cooperative Way, Suite 120
Georgetown, TX, USA 78626
Phone: 512.869.8000
Fax: 512.868.9040
info@radixbiosolutions.com

ORA LABORATORY MLV QUESTIONS / CONCERNS

- **Is the data for the entire plate still viable if the instrument aborts a batch mid-run but the analysis is restarted right away?** **This is a very important question. Some versions of the software have a problem that randomly affects performance (believed to be communication). It is not common to all instruments. Though two instruments may both be using xPONENT® version 4.2, whether one is actually running 4.2.1.3.5 versus 4.2.1.3.7 may make a difference (numbers randomly chosen for illustration purposes). Also, though one instrument may display the problem, another produced alongside it and running the same version-subversion of xPONENT® may be fine. As such, if you have been having these problems, contact the Luminex Service Engineer - tech support (512-381-4397) with the instrument's serial number and xPONENT® version (all numbers). They will decide if the latest version will solve your problem. Doing such will probably require that you download the latest version, erase prior versions, and re-do the calibration / verification beads etc. If you have not been having any problems, do not worry.**

  If the problem occurs during the MLV, resume right away, though you may lose the batch depending how the resume is initiated and everything handled (Luminex may be able to provide guidance); thus, yes to your answer if done correctly. Per adding buffer and redoing the plate, this has potential problems. The dilution will change the concentration of free analyte but as long as all the samples (including standards and controls) are diluted the exact same amount, the shifting along the titration curve could have minimal effect on the final results. The primary concern is whether all wells have exactly 70 µL removed. The precision in the removal of 70 µL is not ideal and thus any differential dilutions would generate misleading data. Further, the 'noise' and degree of variance may change depending on where the 'diluted' samples fall on the titration curve of antigen - antibody binding. Not diluting, raises the problem of air bubbles because the remaining 30 µL is insufficient for another analysis. The option of removing less than 70 µL decreases the assay's robustness and is therefore counter-productive.

  IF during the MLV you experience 'aborts', do the following 3:
  First, ascertain if you can save the batch's data (note such on the file)
  Second, add the exact same amount of buffer to each well (e.g., 70 µL), mix each well using a multichannel pipettor - changing tips between wells, read the plate and note what happened on the file.
  Third, contact CFSAN about receiving new (replacement) samples.

- **If the plate ends up sitting in the instrument for a time due to an aborted run, when are the samples no longer viable for analysis?** **Though the samples are technically still valid, it is important that a plate not sit for more than 2 hours (preferably no sitting). This is to avoid extensive bead precipitation and what might result in sub-population selection. This is one reason we do not let plates run indefinitely to achieve the necessary number of beads (but rely more on a statistical approach in the chosen cut-off parameters, see insert supplied with test kits). Interestingly, by not using a 96-well assay format and having decent bead counts, the idea of prolonged sitting has virtually been eliminated. If**

still prolonged sitting cannot be avoided, the samples in each well would have to be well mixed and CFSAN contacted since it might still be necessary to get replacement samples and re-do the analyses.

- **Can the final volume in the wells be increased so that if issues are encountered, there is enough sample left in the wells to re-run the entire plate without having to redo the assay part? (currently, final volume is 100 µL, and 70 µL is used for analysis). Excellent question. The simple answer is no. The volumes were all chosen to optimize the ruggedness of the assay from numerous perspectives. To sacrifice the heightened ruggedness to compensate for a rare occurrence is counter-productive.**

- **For the MLV, is it acceptable to complete the assay, cover the plate and refrigerate, and do the analysis the next morning to avoid a time crunch between resolving issues and analyzing the plate? No. One of the goals of the training and exercises was to get the analyst comfortable with the assay so such 'time crunches' do not occur. The assay was deliberately divided the assay into 2 phases with the work for each kit divided over two days to reduce any one day's workload. As such, the time it takes to do a single phase for one food is comparable to the time it takes to run a typical ELISA.**

- **Any suggestions on how to break up large bead clumps other than vortexing for many minutes? This should not be a problem. The kits you will be getting will be 'freshly made' and validated for their performance. Thus, the clumping that occurs over time should be virtually non-existent. The training test kits were the worse you should ever see in terms of clumping.** ***An approach taken by some end-users, is to put 50 µL of the bead cocktail (as soon as it is prepared) into each of two wells of the calibration plate or a spare plate (not supplied), each is mixed with 50 µL of PBST, and the two samples counted. This is to confirm the presence and status of the beads in the cocktail. The MFI values should be approximately background and the bead counts should confirm the composition of the cocktail. This is useful when preparing complex cocktails from numerous bead sets. Since, we are not using all 48 wells in an assay, the volume of beads prepared to generate the cocktail (enough for 48 wells) should be sufficient for this test. This is not currently a formal part of the assay (under discussion) and not (currently) required.***

- **Why have the spiking standards for the DCC-CMS and the final sample dilution protocols been changed? These have not been changed from the original plans for the xMAP FADA for use by the FDA. The product insert is a generic set of instructions for all possible end-users. The MLV is designed to validate the method as will be employed by the FDA. The DCC are designed to reflect the agency's focus on critical target levels and potential thresholds. The ELISA protocols were originally focused on cases-for-cause (established health risk) with the controls designed to rule-out false positives and false negatives. Over time, 'special workbooks' incorporating mathematics employing a DCC perspective have become common. Further, the xMAP FADA does not require controls for false positives and false negatives due to the multitude of built-in controls associated with the multiplex design.**

The guideline's specifications of dilutions to be employed for the PBST extracts (10X) and the UD extracts (5X) are consistent with the product insert except the dilutions to use are specified rather than requiring serial dilutions. This is because one focus of the MLV is to ascertain the reliability of the assay when performed by multiple analysts to measure amounts of analyte spanning what have been considered regions of interest while maximizing 'strain' across the dynamic range of the assay. Thus, the fixing of the dilutions versus letting the analysts optimize the dilutions around the center of the dynamic range (inflection point).

Lastly, greater / larger dilutions are manageable with the xMAP FADA than with the ELISAs thanks to the sensitivity, dynamic range, and multiple built-in confirmatory endpoints. This should decrease workload while increasing throughput. There are multiple other advantages as discussed in the training and various presentations.

- **Why must the muffins be diced if they are already pre-weighed? This is / has always been standard protocol when analyzing baked goods. Extensive studies have demonstrated an inverse correlation between particle size (maximizing surface area:volume) and analyte mobilization, extraction, and recovery.**

- **Concerning---Why was the UD buffer variation of the method not integrated into the practice samples but is now included in the MLV? Time and logistics. It was also felt that making UD Buffer was not a demanding task, especially if the procedure of mixing 1:1 200 mM NaPi with PBST plus adding NFDM (not supplied). If using the Sigma pre-packaged PBST, add 25 µL of Tween -20 to compensate for the lower content of the Sigma product*.**

  *** the change from 0.1 % to 0.05 % Tween with the Sigma PBST did not affect its utility. However, a change from 0.05 % to 0.025 % Tween in the UD Buffer (due to the 1:1 dilution with NaPi) was not acceptable.**

- **To clarify, "PBST" only refers to PBS/0.1% Tween-20 and is now used for everything (i.e. spiking standard prep, extractions, dilutions, etc.)? No more using the PBST packets (PBS + 0.05% Tween 20) for extractions and dilutions for the nondenatured method? No, please see prior comment and product insert. Experimentation was conducted to demonstrate that using 0.1 % and 0.05 % was acceptable for the standard uses of PBST but not in the production of UD Buffer (classically, Tween in PBST is 0.1 %).**

  FYI, PBS is also a misnomer, we use 10 mM PBS (10 mM Pi). Sigma also sells a 50 mM Pi PBS. Thus, in all publications, either a detailed description of the PBS or the catalogue number is included.

- **Please clarify the point listed third from the end in the "COMMENTS & MODIFICATIONS" section beginning with "-The S0 calibration standards…" In performing the buffered-detergent protocol, the extraction buffer and the dilution buffer are the same (e.g., extract 1 gram with 10 mL PBST 2hr, centrifuge, and dilute 10X with PBST or extract 1 gram with 40 mL UD Buffer 2 hr, centrifuge, and dilute 5X with UD**

buffer). When performing the reduced denatured extraction: 1 gram is 'dissolved' into 10 mL PBST, mixed with 10 mL SDS/β-mercaptoethanol for 30 min at 60 °C, centrifuged, and then diluted 100X with PBST. Thus, the final solvent of the sample added to the microtiter plate well is (effectively) the SDS/β-mer buffer diluted 200X with PBST. The use of SDS/β-mer diluted in half with PBST is necessary to maximize sample dissolution; the additional 100X dilution with PBST is necessary to accommodate the binding properties of the various antibodies (a net 200X dilution of the SDS/β-mer).

***gentle reminder, UD-Buffer is used for the chocolate samples for the buffered-detergent analyses, UD Buffer is <u>never</u> used in the reduced-denatured analyses.**

- **How critical are the LoBind tubes when preparing the calibrators? Can other microcentrifuge tubes be used? For the MLV you are not being specifically told beyond the phraseology in the product insert regarding the use of low binding tubes (USA Scientific CAT#1415-250 or Eppendorf LoBind). One reason PBST is used is to reduce the loss (as a percentage) of protein due to 'sticking' to the plastic when working with non-complex (e.g., buffer) samples containing very low concentrations of protein. When the lab was set-up, such tubes should have been acquired as instructed in the training and mentioned in the product insert. It is worth the cost.**

- **The reduced denatured method: like the samples, are the CMS and blank samples also diluted 1:100 as well? The method/tip sheets never address this. The method/tip sheet was never designed to repeat or replace the product insert; only to help clarify any points that may be confusing. In the MLV, there are no samples called 'blanks' as was previously used in the ELISA analyses (e.g., CMS R0) or in exercises. This does not mean that one of the coded samples is not lacking a particular analyte. As detailed in the product insert on page 10, for the non-zero (S1-S7) calibration standards, the stock supplied with the test kit is mixed 1:1 with SDS/β-mer (e.g., 10 µL + 10 µL), incubated for 30 min at 60 °C, centrifuged and diluted 100X with PBST (e.g., 10 uL of aqueous supernatant + 990 uL PBST) to generate S7. This is then serially diluted using Diluent (SDS/β-mer diluted 200X with PBST). Diluent is also used to generate S0. This assures that the calibration standards have been processed and the solvent composition is identical to the final solvent milieu of the coded samples (6, each in triplicate) and the DCC (3) samples.**
